# Changes in Gut Microbial Diversity and Correlation with Clinical Outcome in Children with Acute Myeloid Leukemia Receiving Induction Chemotherapy

**DOI:** 10.3390/children12091176

**Published:** 2025-09-03

**Authors:** Mai Adel, Reham Abdelaziz Khedr, Ahmed A. Sayed, Lobna Shalaby, Aya A. Diab, Abdelrahman Yahia, Mervat Elanany, Leslie E. Lehmann, Sonia Ahmed, Ramy K. Aziz, Alaa Elhaddad

**Affiliations:** 1 Department of Pediatric Hematology and Oncology, Children’s Cancer Hospital Egypt 57357, Cairo 11617, Egypt; lobna.shalaby@57357.org (L.S.); sonia.mahmoud@57357.org (S.A.); alaa.hadad@57357.org (A.E.); 2 Department of Pediatric Hematology and Oncology, National Cancer Institute, Cairo University, Cairo 11562, Egypt; 3 Genomics and Epigenomics Research Program, Department of Basic Research, Children’s Cancer Hospital Egypt 57357, Cairo 11617, Egypt; ahmad.sayed@57357.org (A.A.S.); aya.a.diab@outlook.com (A.A.D.); abdelrhmanahmedyahia@gmail.com (A.Y.); 4 Department of Clinical Pathology and Microbiology, Children’s Cancer Hospital Egypt 57357, Cairo 11617, Egypt; mervat.elanany@57357.org; 5 Boston Children’s Hospital, Dana Farber Cancer Institute, Boston, MA 02215, USA; leslie_lehmann@dfci.harvard.edu; 6 Microbiology and Immunology Research Program, Children’s Cancer Hospital Egypt 57357, Cairo 11617, Egypt; 7 Department of Microbiology and Immunology, Faculty of Pharmacy, Cairo University, Cairo 11562, Egypt; ramy.aziz@pharma.cu.edu.eg

**Keywords:** AML, gut microbiota, pediatric oncology, chemotherapy, bioinformatics

## Abstract

**Background:** The gut microbiome affects human health, and patients with cancer are no exception. In those patients, intensive chemotherapy impairs gut barrier integrity, causing dysbiosis, bacterial translocation, and higher infection risk. **Objectives:** This prospective study, conducted at Children’s Cancer Hospital in Egypt, profiles the microbiome of 29 pediatric patients with AML, and examines how induction chemotherapy and antibiotics affect their microbiome. **Methods:** Gut microbiome changes were evaluated before treatment (T1), then 7 (T2) and 21–28 days (T3) from induction start. Microbial DNA, extracted from rectal swabs or stool samples, was subjected to 16S rRNA amplicon sequencing, followed by bioinformatics and statistical analyses. **Results:** Treatment significantly decreased the richness and Shannon diversity of the gut microbiome and caused dysbiosis that was only partially restored at T3. Whereas Firmicutes remained the most abundant phylum throughout, Actinobacteria significantly decreased in abundance after treatment. Proteobacteria had their lowest abundance at T3, while Verrucomicrobacteria were relatively abundant at T1 but undetectable by T3. The abundance of *Enterococcus* and *Klebsiella* was associated with stool culture results, and the Proteobacteria-to-Firmicutes ratio was associated with treatment. **Conclusions:** Gut microbial diversity declined in patients during induction chemotherapy, with a strong association of microbial composition with stool culture results but not with bacteremia.

## 1. Introduction

Acute myeloid leukemia (AML) accounts for 20% of childhood malignancies. It has been historically difficult to cure, but outcomes have improved over the past years, with event-free survival now reaching up to 70% [1,2,3]. This is due to both advances in treatment and improvements in supportive care. Treatment involves intensive cytotoxic chemotherapy (cytarabine and anthracyclines), which compromises the immune function and mucosal barrier integrity, leaving patients vulnerable to infections [4,5].

The intestinal barrier has a critical role in preventing the trillions of bacteria residing in the GI tract from entering the bloodstream, and its dysfunction results in an increased risk of bloodstream infection. Microbial cultures from the blood detect bloodstream infections; however, they have several limitations, including difficulty in obtaining cultures, contamination from skin microbiota, and time to culture positivity [6].

In the past two decades, the human microbiome has been recognized as a key player in a variety of physiological and pathological processes [7,8]. The gut microbiome, in particular, is one of the largest and most complex ecosystems and is influenced by genetic, environmental, and lifestyle factors. Commensal members of the gut microbiome have a significant beneficial effect on human health and well-being through their role in biosynthesis of vitamins and growth factors, digestion of fibers and oligosaccharides, support of human immunity against external pathogens, and finally detoxification of several xenobiotics, including some toxic drug metabolites. On the other hand, some resident microbes are involved in some inflammatory diseases, colorectal cancer, and drug inactivation. Advances in understanding the gut microbiome have been made possible by high-throughput sequencing technologies, including 16S rRNA amplicon sequencing and shotgun metagenomics, which allow the profiling of multiple microbial communities. Sequencing-based taxonomic profiling is superior to culture-based technologies, as it offers an unbiased, more comprehensive, and more quantitative representation of microbial communities [9,10].

The pediatric gut microbiome changes over the course of childhood and is characterized by high variability between individuals and, over time, intra-individual diversity [11,12]. Previous studies assessing gut microbiome modifications during chemotherapy for childhood acute leukemia have shown that antibiotics, chemotherapy and resulting immunosuppression, dietary changes, and direct toxicity are factors that contribute to alterations in the intestinal ecosystem. The microbiome diversity significantly decreases after intensive induction and reinduction chemotherapy, with a slight rebound in the period after recovery from induction [13].

Because of the importance of the gut microbiome and its potential as a source for bloodstream infections (BSIs), it is critical to understand how its composition changes during therapy. The aim of the current study was to describe the fecal microbiome profile of pediatric patients with AML treated at a large children’s cancer hospital and to assess the impact of treatment (chemotherapy and antibiotics) on changes in the microbiome profile during the period of induction.

## 2. Materials and Methods

### 2.1. Ethical Approval

All protocols were approved by the Internal Review Board (IRB) of Children’s Cancer Hospital Egypt 57357, under approval # 61/2019. Written informed consent was obtained from all subjects or their legal guardians.

### 2.2. Study Design

This was a prospective study including 29 consecutive pediatric patients with newly diagnosed AML treated at the Children’s Cancer Hospital Egypt (CCHE-57357) from December 2019 to April 2020. Patients were treated according to the CCHE AML protocol adopted from the modified COG protocol AAML1031 [14] (details of the protocol are provided in Supplementary Diagram S1). All patients were initiated on prophylactic antimicrobial therapy, with levofloxacin as an antibacterial agent and micafungin as an antifungal. Once febrile neutropenia occurred, blood cultures were drawn, and carbapenem/aminoglycoside was given empirically as per the CCHE clinical care pathway for high-risk fever and neutropenia (HR FN). De-escalation was then guided by the general clinical condition of the patient and results of blood cultures. This study was designed to compare the changes in gut microbiota at three time points: **T1**—pre-treatment, **T2**—D7 from start of induction, and **T3**—D21–D28 from start of induction.

### 2.3. Clinical Definitions

Fever was defined as a single oral temperature of 38.3 °C or more or 38 °C over 1 h in the absence of an obvious cause. Neutropenia was defined according to NCCN guidelines as either (1) an absolute neutrophil count (ANC) less than 500/mcL or (2) an ANC less than 1000/mcL predicted to decline to 500/mcL or less over the next 48 h [15].

High-risk patients were those with anticipated prolonged (>7 days’ duration) and profound neutropenia (absolute neutrophilic count (ANC) < 100 cells/mm3 following cytotoxic chemotherapy) and/or significant medical co-morbid conditions, including hypotension, pneumonia, new onset abdominal pain, or neurologic changes [15].

Enterocolitis is the most common cause of intestinal complications that develops during treatment of AML and results from inflammation, hemorrhage, and/or ulceration of the lower intestinal tract. It is diagnosed clinically, supported by radiologic imaging [13].

### 2.4. Sample Collection

Stool samples were collected with a standard stool kit, including a sterile plastic cup and a plastic bag with a zip lock to seal the specimens. Rectal swabs were inserted approximately 1–1.5 inches beyond the anal sphincter and gently rotated to sample the anal crypts. Feces had to be visible on the swab. The sample was transported within 2 h and processed according to standards. Two samples (rectal swabs or stool samples) were collected for each patient at three time points: one sample was sent to the microbiological lab and the other one to the genomics lab.

### 2.5. 16S rRNA Amplicon Sequencing

Microbial DNA was extracted from both rectal swabs and stool samples by the PureLink Microbiome DNA Purification Kit [16] (Invitrogen, ThermoFisher Scientific, Waltham, MA, USA) according to the manufacturer’s instructions. The extracted DNA was quantified in a DeNovix Fluorometer (DeNovix, Wilmington, DE, USA). DNA samples were diluted and prepared according to the Illumina 16S rRNA Protocol. The amplification of the V3–V4 regions was performed by the following commercially available primers: Forward: 5′ TCGTCGGCAGCGTCAGATGTGTATAAGAGACAGCCTACGGGNGGCWGCAG and Reverse: 5′ GTCTCGTGGGCTCGGAGATGTGTATAAGAGACAGGACTACHVGGGTATCTAATCC.

Both the first and second PCRs, called Amplicon PCR and Index PCR, respectively, used Q5^®^ Hot Start High-Fidelity DNA Polymerase and dNTPs solution mix, both from NEB, Ipswich, MA, USA. The amplification conditions of the amplicon PCR mainly consist of an initial denaturation for 30 s at 98 °C, followed by 25 cycles of 98 °C for 30 s, 55 °C for 30 s, and 72 °C for 30 s, followed by the final extension at 72 °C for 5 min. Samples were indexed by the Nextera XT indexes (Illumina, San Diego, CA, USA). After each PCR, the targets were cleaned up by AMPure XP beads (Agencourt AMPure XP, Beckman Coulter, Brea, CA, USA); then, the final amplicons were purified by AMPure XP beads (Agencourt AMPure XP, Beckman Coulter, USA). The libraries were pooled and diluted to the final concentration of 6 pM. Paired-end sequencing was performed on the Illumina MiSeq System with a read length of 300 [17,18].

### 2.6. Bioinformatics Analysis

#### 2.6.1. Amplicon Processing (16S rRNA)

An amplicon sequence variant (ASV) table was created by the DADA2 workflow in R. The raw fastq file was trimmed with Trimmomatic. Trimmed reads were then filtered by DADA2 filterAndTrim with maxEE 8 and 9, truncLen 290 and 270. Filtered reads were merged, and ASVs were then generated with DADA2 default parameters. Sequences and frequency tables were imported into Quantitative Insights in Microbial Ecology 2 (QIIME2), and taxonomy was assigned to the ASVs by a Naïve Bayes classifier compared against the SILVA v.138 reference database.

#### 2.6.2. Definitions

**Differentially abundant bacteria** were identified by DESeq2, and geometric means of un-rarefied ASVs were used for normalization using the estimate size factors function in DESeq2. Low-abundance ASVs and taxa (represented by <2 counts and in no more than 10 samples) were removed to reduce the number of multiple hypotheses for false discovery rate adjustment. **Relative abundance** was defined as the percent composition of an organism of a particular taxon relative to the total number of organisms in the area. **Alpha diversity** was defined as the microbial diversity within the same sample (expressed as richness, evenness, or both). **Beta diversity** was defined as the relative diversity of taxa from one sample to another.

### 2.7. Statistical Analysis

Microbiome data were processed and visualized in R 4.04. Phyloseq was used to calculate alpha and beta diversity. The Kruskal–Wallis test, followed by non-parametric post hoc tests, was performed to test the significance of the treatment’s effect on the diversity among samples with the “ggpubr” R package (version 0.4.0). The Friedman test was used for related samples (repeated measures from the same patients). Statistical differences of the average bacterial community between groups were tested using the adonis function from the “vegan” package (version 2.6–4) to perform permutational multivariate analysis of variance (PERMANOVA).

Other statistical data were analyzed by IBM SPSS Advanced Statistics (Statistical Package for Social Sciences), version 24 (SPSS Inc., Chicago, IL, USA). Numerical data were described as mean (±standard deviation), median, and range. Normality was tested by the Kolmogrov–Smirnov test and the Shapiro–Wilk test. When the variables were not normally distributed, group effect was tested by the Kruskal–Wallis test, followed by the post hoc Dunn test; for time/treatment effect, the Friedman test was used, followed by the Wilcoxon signed-rank test. The *p*-values were adjusted for hyperinflation by the Bonferroni or Benjamini–Hochberg corrections. A *p*-value ≤ 0.05 was considered statistically significant. All tests were two-tailed.

For linear model (LM) and linear mixed model (LMM) analysis, the following R packages were used: “lme4 (1.1–28)”, “lmerTest (3.1–3)”, “broom.mixed (0.7.12)”, and “DHARMa (0.4.7)”. Outcome variables modeled were Shannon diversity, richness, evenness, Firmicutes-to-Bacteroides ratio, and Proteobacteria-to-Firmicutes ratio. Most of the independent variables in the study, including age, sampling time, enterocolitis, different antibiotics, and inter-patient variability, were tested in different models.

## 3. Results

### 3.1. Patient Clinical Characteristics

The median age for the entire cohort of patients with pediatric AML (n = 29) was 7 years (range 0–18). Twenty-seven patients were diagnosed with AML and received induction chemotherapy consisting of ADE (Adriamycin, Ara-C, and Etoposide), while two patients were diagnosed with acute promyelocytic leukemia (AML M3) and received idarubicin, Ara-C, and all-trans retinoic acid (ATRA, Supplementary Diagram S1). Eleven patients (38%) developed enterocolitis (eight of whom had documented radiological findings). Nine patients developed Gram-negative septicemia caused by multi-drug-resistant (MDR) organisms. Four of the nine were rectally colonized by the same organisms (documented with stool cultures) prior to septicemia. Five patients (17.2%) were transferred to the ICU with septic shock, four (13.8%) of whom died (Table 1).

### 3.2. Changes in Diversity of the Gut Microbiota Among Patients with AML

#### 3.2.1. Alpha Diversity

**Figure 1:**
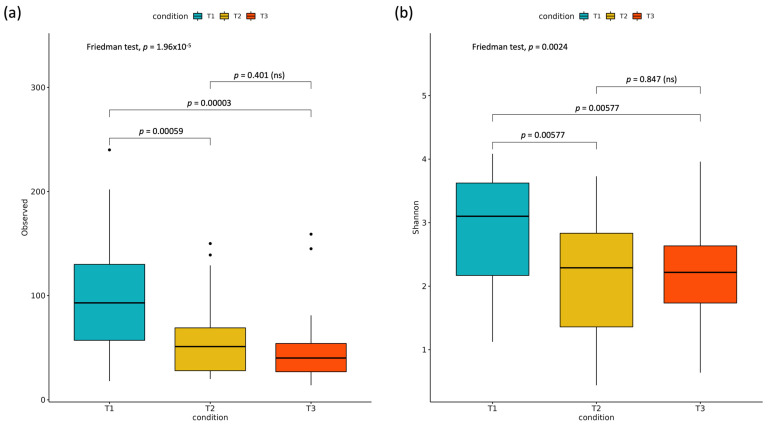
Differences of alpha diversity, expressed as observed OTUs (**a**) or Shannon diversity index (**b**), between the three time points. Statistical significance was assessed by the Friedman test, followed by the Dunn post hoc test with the Benjamini–Hochberg correction. Corrected *p*-values are shown for pairwise comparisons.

The Shannon index for alpha diversity was used for comparing the different microbiomes at the three time points. A significant reduction was found in bacterial diversity at T2 and T3 in comparison to the pre-treatment samples (Figure 1).

#### 3.2.2. Beta Diversity and Signs of Dysbiosis

**Figure 2:**
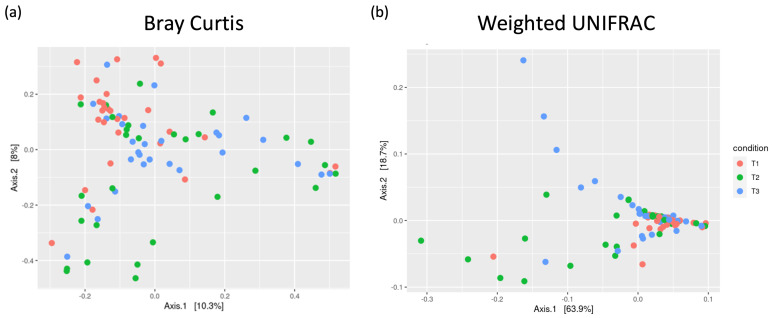
Beta diversity of all samples, estimated by the Bray–Curtis Distance (**a**) or the weighted UNIFRAC metric (**b**). Each circle represents a sample, color-coded by the different conditions/time points.

Principal coordinate analysis (PCoA) of the dissimilarity between different samples was characterized by a high compositional variability between individuals and over time (at the three time points), suggesting (i) a high inter-individual variability among patients that was more pronounced on Day 7 than pre-induction and (ii) a microbial composition in AML patients that was significantly altered at T2 with partial rebound in the period after recovery (T3), with a composition more similar to T1 (*p* = 0.002) (Figure 2). These changes were clearly expressed by the distances to centroids of the Bray–Curtis dissimilarity matrix (Figure 2a) and even more pronounced when the weighted UNIFRAC measure of diversity was used (Figure 2b).

### 3.3. Distribution of Bacterial Phyla in the Gut Microbiota Among Pediatric Patients with AML

**Figure 3:**
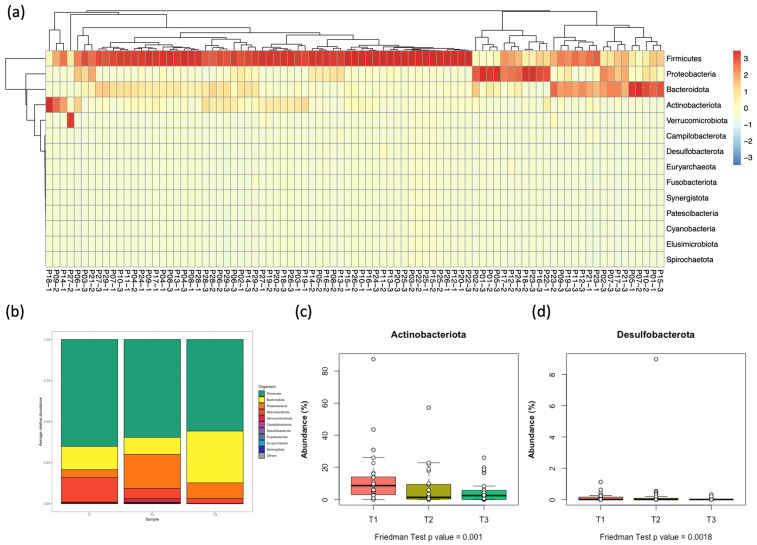
Microbial composition differences at the phylum level. (**a**) A column-normalized heatmap representing the relative abundance of the top 14 phyla in the microbial community of all samples. Rows represent the major phyla, sorted by overall abundance. Columns represent different samples, clustered by pattern. The heatmap highlights four major microbiome types (“enterotypes”), the largest of which is dominated by Firmicutes. (**b**) A stacked bar plot representing the relative abundance and distribution of major phyla for each of the three groups (time points). (**c**,**d**) Relative abundance (%) of the two phyla ((**c**): Actinobacteria and (**d**): Desulfobacterota) whose differences are statistically significant among groups (Friedman test *p*-value ≤ 0.05). Exact *p*-values are shown.

In the 29 patients, four bacterial phyla with the greatest abundance were identified (Table 2). The most common was Firmicutes (58.5%), followed by Bacteroidota (18.4%) and Proteobacteria (15.6%), while Actinobacteriota was the least abundant (7.7%) of the four (Figure 3, Figures S1 and S2). A significant decrease in the relative abundance of Actinobacteriota was observed between T1 and T3 (median = 7.55% vs. 2.35%, post hoc *p* = 0.011, Figure 3c and Supplementary Table S1). The minor phylum, Desulfobacterota, also significantly varied among groups (Figure 3d). No significant changes were detected among the other phyla over the assessed period.

### 3.4. Distribution of Bacterial Genera in the Gut Microbiota of Pediatric Patients with AML

The genera detected with the highest abundance were *Streptococcus* (30.4%), *Lactobacillus* (25.64%), *Bacteroides* (14.53%), and *Enterococcus* (9.73%). Significant changes in the relative abundance of *Enterococcus* and *Streptococcus* were observed between T1 and points T2 and T3 (*p* = 0.03 and *p* < 0.001, respectively). The relative abundance of *Lactobacillus* was higher at T2 and T3 than at the pre-treatment period (T1).

### 3.5. Differential Abundance at Phylum and Genus Levels at the Three Time Points

**Figure 4:**
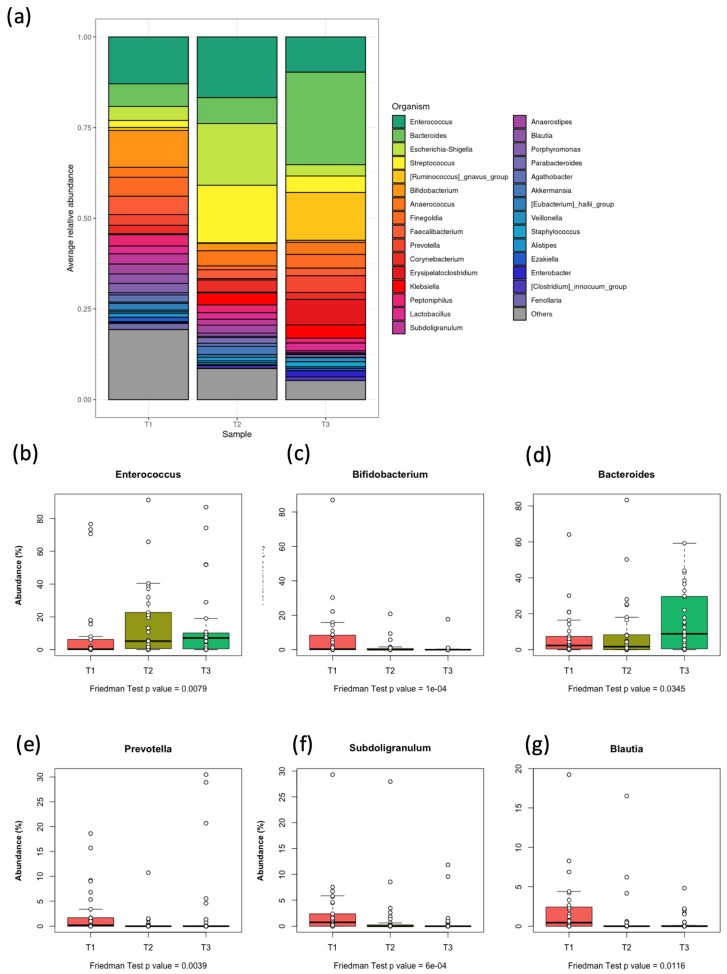
Microbial composition differences at the genus level. (**a**) A stacked bar plot representing the relative abundance and distribution of major genera for each of the three groups (time points). (**b**–**g**): Relative abundance (%) of representative genera whose differences are statistically significant among groups (Friedman test *p*-value ≤ 0.05). Exact *p*-values are shown. The genera (named above each chart) are six of the 10 most abundant and statistically significantly different ones between treatment groups.

Certain bacterial genera were only detected after the start of therapy, such as *Klebsiella*, which appeared after 7 days of treatment and increased until after Day 21. This reflects the marked changes in the relative abundance of certain genera, which may influence the clinical condition of pediatric patients with AML (Figure 4, Figures S1 and S2). Among the statistically significantly altered organisms, the 10 most abundant genera were *Enterococcus*, *Bacteroides*, *Faecalibacterium*, *Bifidobacterium*, *Prevotella*, *Subdoligranulum*, *Ruminococcus gnavus* Group, *Blautia*, *Anaerostipes*, and *Eubacterium coprostanoligenes*.

Although Firmicutes remained the most abundant phylum (Supplementary Table S1), and although there was no significant difference in its abundance between the three time points (*p* = 0.067), the internal composition of this phylum’s members substantially varied. Notably, the genera *Enterococcus* (Figure 4b) and *Streptococcus* increased at T3, in contrast with the slight decrease in the median relative abundance of the entire phylum (55.35% at T3 vs. 68.33 at T1 and 64.52 at T2, Supplementary Table S1). Additionally, the differences between Actinobacteriota among samples seem to have mostly been contributed by the genus *Bifidobacterium* (Figure 4c).

### 3.6. Differential Abundance of Microbial Taxa in Relation to Stool and Blood Cultures

**Figure 5:**
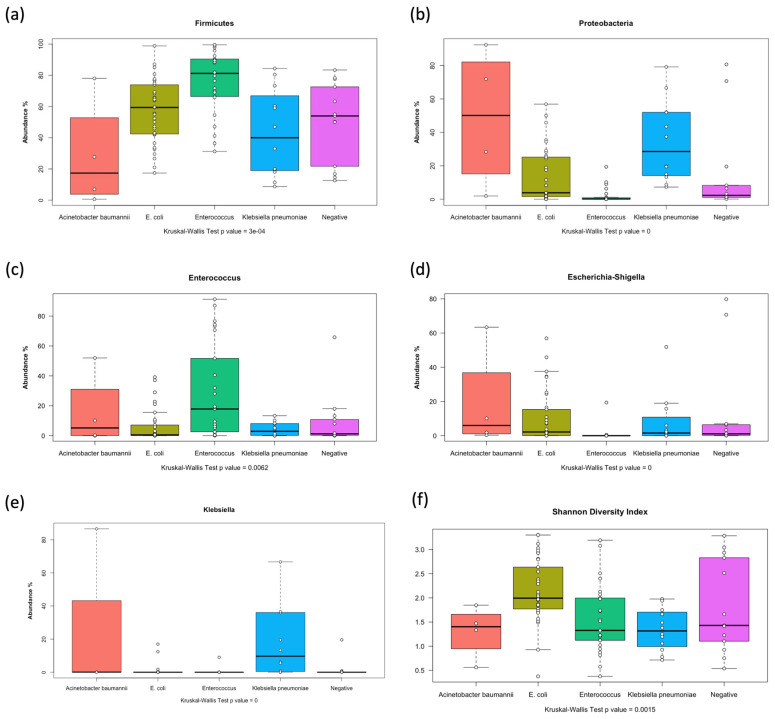
Boxplots representing the relative abundance (%) of major bacterial phyla (**a**,**b**) or genera (**c**–**e**) in the different samples vs. the microorganism isolated from stool in the clinical microbiology laboratory. (**f**) Relation between the Shannon diversity index and stool culture results. All differences shown are statistically significant among different subsets (Kruskal–Wallis *p*-value < 0.05).

One of the goals of this study was to investigate the association between microbiology laboratory results (stool and blood cultures) and the microbiome composition. The results of 16S microbiota profiling were tested against the major bacteria detected in stool and blood cultures. Stool culture results were significantly associated with several attributes of the microbiome. First, all alpha diversity estimates (richness, evenness, and the Shannon diversity index) significantly varied in relation to the stool culture results. Higher alpha diversity was observed in patients with *E. coli* detected in their stool, while the detection of *Enterococcus*, *Klebsiella*, and *Acinetobacter baumannii* was associated with lower genus-level alpha diversity metrics (Figure S3 and Figure 5f). More intriguingly, the relative abundance of the phylum Firmicutes was highest in microbiomes of patients who had positive *Enterococcus* stool cultures (Figure 5a); on the other hand, the relative abundance of the phylum Proteobacteria was higher in microbiomes of patients who had positive stool cultures for *A. baumannii* and *Klebsiella* (Figure 5b). At the genus level, this differential relative abundance was observed with the genera *Enterococcus*, *Escherichia*, and *Klebsiella*. Expectedly, *Enterococcus* relative abundance was highest (Figure 5c), while *Escherichia/Shigella* relative abundance was lowest among the cases of *Enterococcus*-positive stool cultures (Figure 5d). On the other hand, *Klebsiella* relative abundance was highest in the cases of *Klebsiella*-positive stool cultures (Figure 5e).

Conversely, blood culture results were not significantly associated with the relative abundance of the isolated organisms nor with the results of stool culture (Table 3). Only a few gut microbial taxa were significantly differentially abundant in association with the organism isolated from blood cultures (Supplementary Table S2); however, neither of them has a known biological connection to sepsis. There were cases in which the organism isolated from the blood was dominant in the microbiome (e.g., Patient 1/Patient 14 and Patient 16). One patient had *Klebsiella* isolated from the blood in association with a major dominance of the genus *Klebsiella* in the microbiome (~85% abundance). Another two patients had bacteremia with *Klebsiella* before the organism dominated the intestinal microbiome.

**Figure 6:**
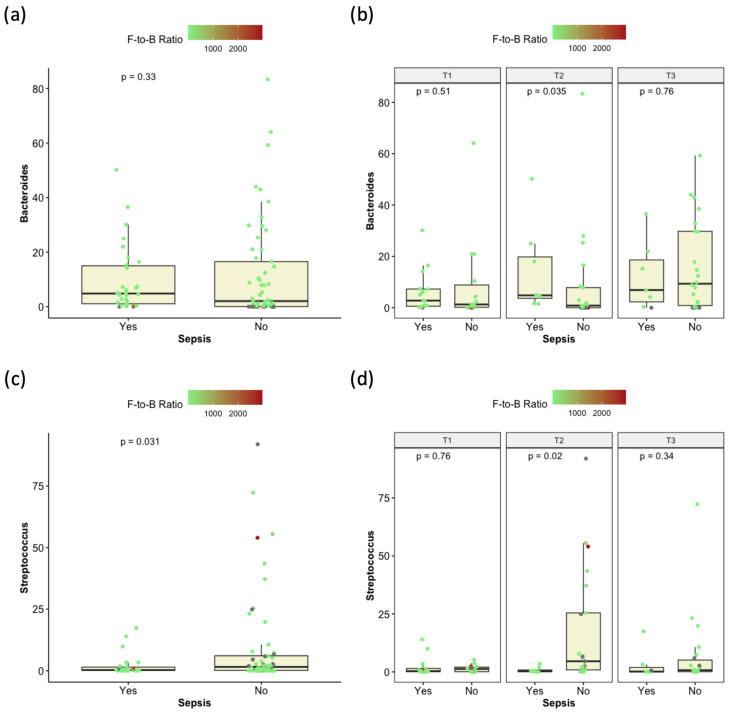
Dot-overlaid boxplots demonstrating the differential abundance of the genera *Bacteroides* (**a**,**b**) and *Streptococcus* (**c**,**d**) relative to sepsis (positive blood culture). (**b**,**d**): Comparisons are conducted for each time point separately. Dots are colored based on the Firmicutes-to-Bacteroidetes ratio. All *p*-values are computed with the Wilcoxon test.

Five genera were significantly differentially abundant in cases diagnosed with sepsis (positive blood culture), the most prominent of which were *Streptococcus* and *Anaerostipes* (Supplementary Table S3). Of interest, when the data were compared for each time point (T1, T2, and T3), genus *Bacteroides* was found to be significantly higher in the microbiomes of patients with sepsis only at T2, and the statistical significance of *Streptococcus* relative abundance in relation to sepsis was also limited to T2 (Figure 6).

### 3.7. Correlation Between Microbiome Composition and Enterocolitis

Enterocolitis is a commonly observed chemotherapy-associated adverse events, and—in this study—nine out of 29 patients presented with enterocolitis. The microbiome composition was compared in patients with and without enterocolitis, and eight genera were differentially abundant (Supplementary Table S4 and Supplementary Figure S4). In addition, microbiome samples from patients with enterocolitis had a significantly lower richness (Supplementary Figure S4). On the other hand, no significant association was found between enterocolitis and stool culture results (chi-square test *p*-value = 0.3823).

### 3.8. Compositional Variation of the Microbiome in Patients Receiving Meropenem and Piperacillin/Tazobactam

In this cohort of patients, two broad-spectrum antibiotics were predominantly used: piperacillin/tazobactam (Tazocin^®^) was prescribed to patients with fever before or during induction, while meropenem was prescribed in response to febrile neutropenia or enterocolitis. To investigate whether these antibiotics may have differentially affected some members of the microbiome, we analyzed the potential association of taxonomic profiles and the administered antibiotic.

Among the major taxa whose relative abundance significantly (*p* < 0.05) increased upon use of Tazocin were *Anaerococcus*, *Corynebacterium*, *Finegoldia*, *Peptoniphilus*, *Campylobacter*, and *Acinetobacter*, while *Intestinibacter* relative abundance was reduced. On the other hand, the use of meropenem had a dramatic effect on microbial richness (median 38.36 vs. 27.6), as well as a decrease in the relative abundance of *Bifidobacterium*, *Ruminococcus*, and *Christensenellaceae*. Patients who received meropenem had significantly higher proportions of *Klebsiella* and *Streptococcus* (Supplementary Table S5).

### 3.9. Other Clinical and Phenotypic Associations

As stated above, while there was an association between some stool-detected bacteria (e.g., *Enterococcus*, *Klebsiella*, and *A. baumannii*) and their abundance in the gut microbiome, there was no such correlation found with blood culture results. This was also highlighted by a lack of association between blood and stool culture results (chi-square test, *p*-value = 0.7214). Thus, stool culture results are not predictive of which organism is found in episodes of bacteremia. The stool- and blood-isolated organisms were the same in only four out of 10 patients who had positive blood culture results.

Of note, although all four patients who died during the observation period had positive blood cultures (Table 2), no common organism was associated with death in all four cases. Yet, an enrichment of the genera *Klebsiella* and, curiously, *Bacteroides* in the fecal microbiome of those four patients was observed (Mann–Whitney *p*-value = 0.0063 and 0.002, respectively).

**Figure 7:**
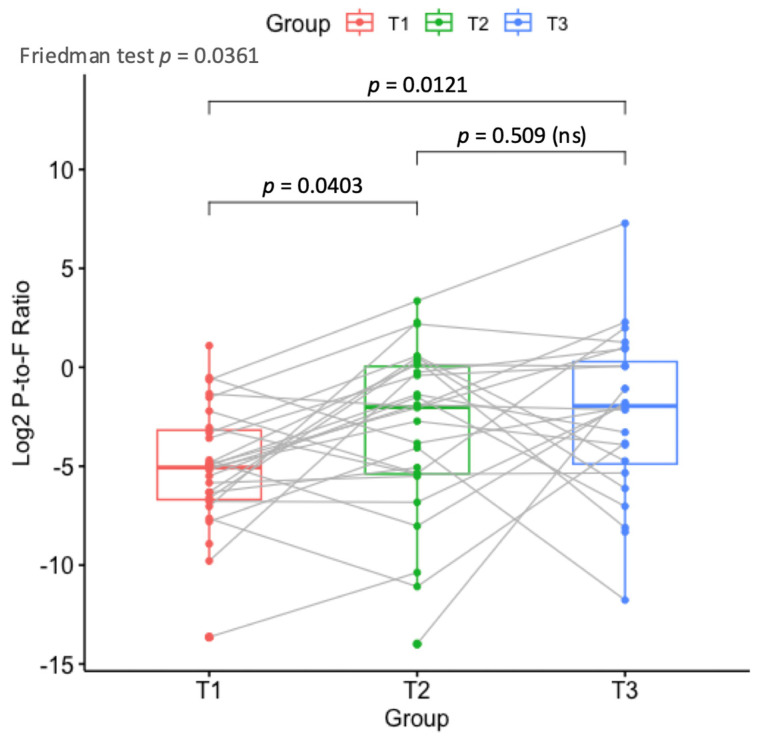
Dot-overlaid boxplots demonstrating the compositional change in the balance between Proteobacteria and Firmicutes (expressed as log2 P-to-F abundance ratio) across time points. The medians of the log2 values are compared with the Friedman test for significance, and the *p*-value is shown. Gray lines connect samples from the same patient to demonstrate inter-patient variability. Post hoc tests for significance were conducted with the Dunn test followed by the Benjamini–Hochberg correction. Corrected *p*-values are shown. ns = non-significant difference.

Subsequently, two commonly used biomarkers in microbiome studies were calculated and correlated with different clinical factors: the Firmicutes-to-Bacteroidetes and Proteobacteria-to-Firmicutes ratios. While the first ratio was not associated with treatment stage, the balance between Proteobacteria and Firmicutes was significantly associated with the stage of treatment. Specifically, 28 out of the 29 patients started (T1) with a higher abundance of Firmicutes than Proteobacteria (Figure 7). However, at T2 and T3, seven and eight patients had higher proteobacterial ratios, respectively, and overall, there was an association between a higher proteobacterial abundance and the treatment stage (Friedman test *p*-value = 0.0361 and chi-square *p*-value = 0.037).

Additionally, when the microbiome profiles were classified into two groups, depending on which phylum was more abundant (F > P or P > F), the Firmicutes-rich samples were highly associated with *Enterococcus* isolation from stool (chi-square *p*-value = 0.00016). All stool cultures that were *Enterococcus*-positive (n = 25) had a lower Proteobacteria-to-Firmicutes ratio. There was no such association with blood culture results.

### 3.10. Accounting for Inter-Patient Variability and Assessment of Significant Factors via Linear Mixed Models

Having tested the univariate effects of multiple factors on the microbiome’s diversity, composition, and key biomarker ratios, we set out to test multiple factors by linear mixed models (LMMs) to assess the most significant predictor among the different factors while accounting and correcting for inter-patient variability.

Multiple LMMs were built, tested, and compared, and the most optimal models were selected (Appendix in Supplementary Material). Among the key findings was that chemotherapy, rather than stage (T1, T2, or T3), was a significant predictor of the microbiome composition and diversity. Additionally, enterocolitis and antibiotic treatment had significant effects on alpha diversity measures, regardless of treatment stage. Patient age had a statistically significant—but mild—effect on diversity.

More specifically, chemotherapy was shown to be associated with lower diversity. The Shannon diversity index was 29% lower in fecal samples before treatment (T1) than in the samples from the same patients after chemotherapy (T2 and T3). Age had a mild effect on Shannon diversity (3% higher diversity per year of age). Enterocolitis had a significant effect as well: with all other factors set aside, including inter-individual variation, patients with enterocolitis had 26% lower Shannon diversity than those with no reported enterocolitis.

Richness, i.e., number of taxa, was even more sensitive to chemotherapy, enterocolitis, and administration of tigecycline. Pre-chemotherapy samples were 50% richer than post-treatment samples (T2 and T3). Patients with no enterocolitis had 40% higher richness.

Finally, stool culture results were highly associated with the Firmicutes-to-Bacteroides ratio. Specifically, the few cases in which *A. baumannii* was detected in stool (n = 4, Table 3) were characterized by a significantly lower Firmicutes-to-Bacteroides ratio (25× lower than when *E. coli* was detected, 26× lower than when *K. pneumoniae* was detected, and 107× lower than when stool was culture negative). All statistics and *p*-values are provided in the Appendix (Supplementary Material).

## 4. Discussion

The intestinal microbial community includes over 300 billion cells, dominated by the phyla Firmicutes, Bacteroidetes, Proteobacteria, Actinobacteria, Fusobacteria, and Verrucomicrobia [19]. Three decades of genomic research have established that the human gut microbiota has a major impact on human health, disease, and response to therapy [7,20,21]. In children with cancer, the gut microbiota, with its rich genomic content and metabolic potential, is of particular interest, not only because of its direct implication in host physiology but also because it represents a reservoir for potential extra-intestinal infections, notably when intestinal barriers are disrupted.

Bacterial and fungal infections due to chemotherapy-induced myelosuppression are an important cause of morbidity and mortality in patients with hematological malignancy, particularly those with AML. The GI tract is a vulnerable site of injury in patients who receive intensive chemotherapy, which may disturb the microbial composition and integrity. Many infections result from bacterial translocation across damaged membranes, including the intestinal mucosa. Therefore, profiling the microbiome right before treatment initiation could reflect microbiome health [22]. The ultimate goal would be to identify patients who are particularly vulnerable to colitis or bacteremia and adjust prophylactic and preemptive strategies. Additionally, chemotherapy-associated dysbiosis can affect the microbiome composition and may affect the outcome. The gut microbiome thus may serve as a biomarker for predicting both chemotherapy-associated toxicity and overall outcome [23,24].

Previous studies on pediatric patients with AML who underwent hematological stem cell transplant (HSCT) and pediatric patients with ALL who received induction chemotherapy observed significant gut microbiota modifications during chemotherapy. These microbiome alterations were attributed to multiple factors, including antibiotic therapy, immunosuppression, dietary changes, and direct drug toxicity [25,26]. Chemotherapy has been reported to affect the intestinal microbiota, which subsequently aggravates mucositis via signaling through toll-like receptors (TLRs), increasing the expression of inflammatory mediators and decreasing epithelial cell differentiation and mucosal regeneration [27]. Chemotherapy may also exert a direct effect on the gut microbiota, among other pharmacomicrobiomic interactions [21,28]. For example, daunorubicin and etoposide were reported to have a negative effect on the growth of anaerobic and aerobic bacteria in vitro [13].

Rattanathammethee et al. [5] reported that the previously detected increase in bacterial abundance in the *Enterococcaceae* and *Streptococcaceae* families of the Firmicutes phylum was a strong predictor of infectious complications in pediatric ALL and adult AML patients [5]. Hakim et al. [13] examined 199 children with ALL who had undergone chemotherapy and allogeneic stem cell transplant and concluded that the gut microbiome composition, but not diversity, was a predictor of infections during chemotherapy. They found that the increase in relative abundance of the phylum Proteobacteria and genus *Enterococcus* during the peri-transplant period was significantly associated with BSI and the likelihood of bacterial translocation.

Several studies highlighted that alterations in the composition of the gut microbiome are associated with GI toxicities, such as typhilitis and colitis. Montassier et al. [29] reported a decrease in relative abundance of *Faecalibacterium* and *Bifidobacterium*; increased *Bacteroides*, Proteobacteria, and *Escherichia*; and, at the genus level, significant decreases in the abundance of *Ruminococcus*, *Oscillospira*, *Blautia*, *Lachnospira*, *Roseburia*, *Dorea*, *Coprococcus*, *Anaerostipes*, *Clostridium*, *Collinsella*, and *Bifidobacterium* in non-Hodgkin’s lymphoma patients [29]. A subsequent study on patients with AML reported that enriched *Staphylococcus*, *Streptococcus*, *Akkermansia*, *Subdilogranulum*, and *Pseudobutyrivibrio* were associated with risk of infections [30]. Another study reported that gut colonization by *Stenotrophomonas* prior to chemotherapy was associated with risk of infection in patients with AML [31]. The same researchers, in another study, compared the diversity and composition of the gut microbiome with fecal samples from patients before and after chemotherapy, and reported significant decreases in the abundance of the genera *Ruminococcus*, *Oscillospira*, *Blautia*, *Lachnospira*, *Roseburia*, *Dorea*, *Coprococcus*, *Anaerostipes*, *Clostridium*, *Collinsella*, *Adlercreutzia*, and *Bifidobacterium*.

Our study describes the changes over time in the gut microbiota in newly diagnosed pediatric patients with AML at a single center in a previously unsurveyed geographical location. This cohort is of interest given the known effect of ethnicity and geographical location, both of which affecting dietary habits, on the gut microbiome [21,28]. The key findings are as follows: (i) a significant decrease in microbial diversity after chemotherapy induction, followed by a partial restoration of diversity through selective rebound of some bacterial taxa (e.g., *Ruminococcus gnavus*) and decline of others (e.g., *Enterococcus* and *Actinomyces*); (ii) a significant association between bacteria cultured from stool and the abundance of some bacterial taxa in the gut microbiome, with a specific association with the Firmicutes-to-Bacteroides ratio; (iii) the lack of clear association between bacteria isolated from blood cultures and their overabundance in the gut; (iv) an association between low microbiota richness and enterocolitis or the use of tigecycline; and finally (v) a strong association between induction of chemotherapy and the Proteobacteria-to-Firmicutes ratio.

Despite a high local prevalence of antibiotic use in the community, the baseline microbiota, before starting chemotherapy, was relatively diverse, and this diversity decreased significantly with treatment when measured with alpha and beta diversity at T2 and T3. Low baseline alpha diversity has been reported as a risk factor for infections, while a decrease in alpha diversity during chemotherapy is often observed [32]. Changes in the gut microbiomes of patients with ALL, AML, and HSCT during induction chemotherapy have been frequently reported. For example, Hakim et al. reported that, during treatment, the relative abundance of some taxa was significantly altered. Members of the Bacteroidetes phylum decreased, whereas other taxa, such as those belonging to the *Clostridiaceae* and *Streptococcaceae* families, increased [13].

In our study, we investigated the association between stool culture results and microbiome composition for each patient. We concluded that they were partly correlated to each other. For example, by calculating the Firmicutes-to-Bacteroidetes and Proteobacteria-to-Firmicutes ratios, we found that the balance between Proteobacteria and Firmicutes was significantly associated with the stage of treatment, while the Firmicutes-to-Bacteroidetes ratio was associated with stool culture results, as singled out by LMMs.

Pathogenic organisms may flourish when commensals decrease. One such organism is the genus *Klebsiella*, which appeared in blood cultures during the neutropenic period in six patients and was correlated with infectious complications, including enterocolitis and even death from sepsis. *Klebsiella* is known to have pathogenic strains and is well documented for multi-drug resistance.

We report that blood cultures were not significantly associated with the relative abundance of isolated organisms, except for three cases in which the organism isolated from the blood was dominant in the microbiome (patient 1, patient 14, and patient 16). Intriguingly, two of those patients died (patient 1 and patient 14). One patient had *Klebsiella* isolated from the blood in association with a major dominance of the genus *Klebsiella* in the microbiome (85% abundance). Another two patients had BSI with *Klebsiella* before the organism dominated in the intestinal microbiome. The lack of correlation between BSI and stool culture results indicates that the source of blood-detected organisms might be of environmental origin, healthcare-associated, or from another microbiome (i.e., skin, oral, and respiratory). Tracing the source of the exact organisms behind BSI is beyond the scope of this study. It requires extensive shotgun metagenomic sequencing, followed by metagenome-assembled genomic analysis, and finally by high-resolution comparative genomics to identify relatedness or clonality.

Finally, further statistical analyses highlighted some possible indicative biomarkers. For example, the positive association between the detection of *Enterococcus* and *Klebsiella* in stool samples and their overabundance in the microbiome might be of potential diagnostic value. Likewise, the lower relative abundance of *Streptococcus* and higher relative abundance of *Bacteroides* in the microbiomes of patients with sepsis may be further investigated and validated for potential clinical and diagnostic applications. As inter-patient variability is known to be remarkable in all microbiome studies, the use of LMMs allowed minimization of this variability and confirmed chemotherapy induction and enterocolitis as key players in affecting the microbiome diversity and composition.

It is worth noting that proteobacterial expansion has been described as a sign of dysbiosis often associated with chemotherapy [33]. This was true in this study as well: microbiomes with higher relative abundance of Firmicutes were also the ones in which *Enterococcus* was more frequently detected. A lower alpha diversity (decrease in genera richness) was associated with enterocolitis. Although causality cannot be established without a relevant animal model, it is plausible to propose that enterocolitis arises because of low microbial diversity or disproportionate expansion of one organism. Another interpretation may be that changes in the intestinal tissues may select for specific bacteria, i.e., that the observed changes are consequential rather than causal. The two interpretations are not mutually exclusive: i.e., a drop in diversity may cause enterocolitis, which in turn causes more dysbiosis. Potential interventions against dysbiosis during chemotherapy range from precision nutrition and supplementation with prebiotics or probiotics to autologous or heterologous fecal microbiome transplantation. Another type of intervention is precise removal of specific taxa by bacteriophage therapy [34].

The common practice of concurrently using antibiotics with chemotherapy, either as prophylaxis empirically in the setting of fever or for treatment of bacteremia, profoundly impacts intestinal homeostasis. Antibiotics lead to gut dysbiosis, which impacts the diversity and richness of the gut microbiota [34]. This consequently impairs the microbiome-mediated colonization resistance and leads to an overgrowth of microbes with antibiotic resistance genes [35]. Here, we focused on two commonly prescribed antibiotics in our center (meropenem and tazobactam/piperacillin), and we found a specific “footprint” for both antibiotics on the microbiome composition. Meropenem was clearly associated with a drop in microbial richness, which is expected given its broad spectrum. One of the bacteria that survived well in the setting of meropenem use is *Klebsiella*, one of the most common members of carbapenem-resistant *Enterobacteriaceae* [36]. On the other hand, some bacteria with probiotic potential were almost completely depleted in patients who received meropenem (e.g., *Bifidobacterium* and *Ruminococcus*). With tazobactam/piperacillin, the bacterial pattern was different: the relative abundance of *Intestinibacter*, a member of the family *Clostridiaceae*, was strongly reduced, while the abundance of a few bacterial genera, including *Campylobacter* and *Acinetobacter* (with known pathogenic species), increased.

Our study has limitations to address in future work. For example, a larger sample size would increase the statistical power, especially for BSI associations, since only 15 out of 29 patients had positive blood cultures. This limitation is due to the nature of the disease, the slow patient enrollment due to parental hesitation to consent, the exclusive recruitment of pediatric patients with a uniform pattern of clinical course, specifically treatment-naive patients undergoing a homogenous course of chemotherapy. Additionally, 16S rRNA amplicon analysis can only confidently predict the bacterial genera and a limited number of species. For source tracking of bloodstream infections, shotgun sequencing or whole-genome analysis is necessary for strain-level resolution [37].

## 5. Conclusions

Gut microbiota diversity significantly decreased after intensive induction protocols, with a slight improvement of dysbiosis in the period after recovery from induction. Specific microbial profiles/patterns that are associated with chemotherapy or antibiotic use were detected. The loss of fecal microbiota diversity can be used to predict and therefore address future potential complications associated with the treatment of these vulnerable pediatric AML patients. Further microbiome research in pediatric patients diagnosed with AML may allow for prediction of anticipated complications as well as antibiotic de-escalation to avoid the emergence of more resistant organisms.

## Figures and Tables

**Table 1:** Patient characteristics.

Characteristics	Categories	Number (%)
**Age (Years)**	≤7	15 (51.7%)
	>7	14 (48.3%)
**FAB Classification**	AML	27 (39.1%)
	AML M3	2 (6.9%)
**Period of Febrile/Neutropenia (Days)**	≤25	14 (48.3%)
	>25	15 (51.7%)
**ICU Admission**	Admission for Septic Shock	4 (13.7%)
	Others	1 (3.5%)
	Not Admitted to ICU	24 (82.8)
**Enterocolitis (Typhlitis + Colitis)**	No	18 (62%)
	Yes	11 (38%)
**CT Abdomen Finding**	No	21 (72.4%)
	Yes	8 (27.5%)
**Died**	No	25 (86.2%)
	Yes	4 (13.8%)
**Cause of Death**	Pulmonary Hemorrhage	1 (25%)
	Septic Shock	3 (75%)
		**Total number = 29**

**Table 2:** Characteristics of 9 AML patients with microbiologically proved bloodstream infection during induction.

Code	Sex	Age (Years)	Time to Neutropenia (Days)	Duration of Neutropenia (Days)	Rectal Swab at Initial Diagnosis (T1)	Microbiological Blood Culture	Fate
**P06**	Male	11	5	16	*E. coli* (carbapenem-resistant)	*E. coli* (carbapenem-resistant)	Alive
**P14**	Male	7	3	13	*Klebsiella pneumoniae* (MDR)	*Klebsiella pneumoniae* (MDR)	Died
**P13**	Male	13	8	15	*E. coli* (ESBL)	*Klebsiella pneumoniae* (MDR)	Alive
**P7**	Male	2	3	20	Normal fecal microbiota	*Klebsiella pneumoniae* (MDR)	Died
**P14**	Male	7	5	17	Normal fecal microbiota	*Klebsiella pneumoniae* (MDR)	Alive
**P16**	Female	3	6	7	*Klebsiella pneumoniae* (MDR)	*Klebsiella pneumoniae* (MDR)	Alive
**P26**	Male	4	7	15	Normal fecal microbiota	*Klebsiella pneumoniae* (MDR)	Alive
**P1**	Female	9	4	20	*Acinetobacter baumannii* (MDR)	*Acinetobacter baumannii* (MDR)	Died
**P28**	Male	10	7	19	*E. coli* (ESBL)	*Acinetobacter baumannii* (MDR)	Died

FN: fever neutropenia; BM: bone marrow; CRE: carbapenem-resistant *Enterobacteriaceae* (*E. coli* or *Klebsiella* or *Enterobacter* organism); MDR: multi-drug-resistant organism (resistant to at least one agent in more than three classes of antibiotics).

**Table 3:** Stool vs. blood culture results.

	Blood Culture	*A. baumannii*	*E. coli*	*K. pneumoniae*	*MRSA*	*Staphylococcus*	*S. mitis*	Negative	Sum
Stool Culture									
*A. baumannii*		1		1				2	4
*E. coli*		1	2	3		4	1	22	33
*Enterococcus*			2	4	1	2		16	25
*K. pneumoniae*				3		1		8	12
Negative				3		1		9	13
Sum		2	4	14	1	8	1	57	87

## Data Availability

Sequencing data were deposited in the National Center for Biotechnology Information (NCBI) under Bioproject accession number PRJNA1158339.

## Supplementary Materials

The following supporting information can be downloaded at https://www.mdpi.com/article/10.3390/children12091176/s1, Supplementary Diagram S1: Treatment Protocol Roadmap adopted from modified COG AAML1031; Table S1: Distribution and statistics of major bacterial phyla at different time points; Table S2: List of genera that are significantly differentially abundant in relation to blood-isolated microorganisms; Table S3: List of genera that are significantly differentially abundant in patients with bloodstream infection (sepsis); Table S4: List of genera that are significantly differentially abundant in patients with enterocolitis; Table S5: List of features that are significantly differentially abundant in patients who received meropenem; Figure S1: Heatmap-colored correlation matrix between all samples based on their microbial composition; Figure S2: Heatmap-colored correlation plots between major bacterial phyla and genera based on their co-occurrence in different samples; Figure S3: Relation between genus-level alpha diversity metrics and stool culture results; Figure S4: Statistically different features distinguishing patients with and without enterocolitis. Appendix: All linear models and LMMs computed and fitted in this study.

## Abbreviations

The following abbreviations are used in this manuscript:

AML	Acute myeloid leukemia
ALL	Acute lymphoblastic leukemia
HSCT	Hematological stem cell transplant
BM	Bone marrow
TLR	Toll-like receptors
GI	Gastro-intestinal
BSI	Bloodstream infection
HRFN	High risk fever neutropenia
CRE	Carbapenem-resistant Enterobacteriaceae
ANC	Absolute neutrophilic count
ASV	Amplicon sequence variant
QIIME2	Quantitative Insights in Microbial Ecology 2
ADE	Adriamycin, Ara-C, and Etoposide
AML-M3	Acute promyelocytic leukemia
ATRA	All-trans retinoic acid
MDR	Multi-drug-resistant
ICU	The intensive care unit
PCoA	Principal coordinate analysis
