# Supplementary material for: Changes in Gut Microbial Diversity and Correlation with Clinical Outcome in Children with Acute Myeloid Leukemia Receiving Induction Chemotherapy
Source: Children (Basel). 2025 Sep 3;12(9):1176. doi: 10.3390/children12091176 (PMC12469140; doi:10.3390/children12091176)
Supplement: Supplementary file 1 [file children-12-01176-s001.zip › children-3570154-supplementary.pdf]

Supplementary Diagram 1: Treatment Protocol Roadmap adopted from modified COG AAML 1031

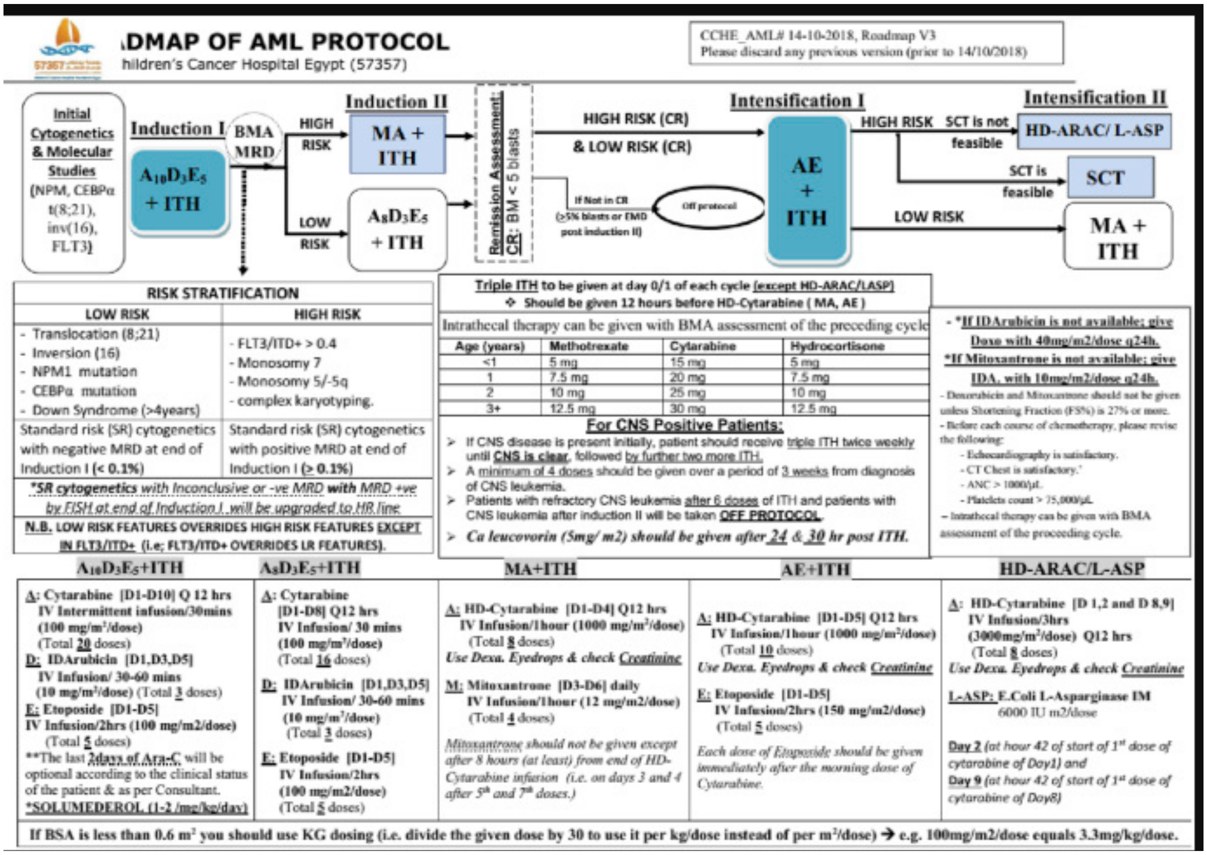

Table S1: Distribution and statistics of the relative abundance (%) of major bacterial phyla at different time points

Phylum	Summary Statistics	T1	T2	T3	overall *p*-value (time effect)	T1 *vs.* T2	T1 *vs.* T3	T2 *vs.* T3
**Actino-bacteriota**	Mean	12.79	**6.63**	**4.90**	**0.002**	1.000	**0.006**	**0.011**
	Median	7.55	**1.45**	**2.35**				
	Std. Deviation	17.45	12.10	7.28				
	Minimum	0.00	0.00	0.00				
	Maximum	84.99	57.17	25.51				
**Bacteroidota**	Mean	19.12	14.09	23.35	0.136	NA	NA	NA
	Median	17.22	6.02	19.90				
	Std. Deviation	21.66	19.92	22.68				
	Minimum	0.00	0.00	0.00				
	Maximum	94.47	82.87	100.00				
**Firmicutes**	Mean	62.43	61.11	52.65	0.067	NA	NA	NA
	Median	68.33	64.52	55.35				
	Std. Deviation	24.13	29.14	27.93				
	Minimum	5.53	7.62	0.00				
	Maximum	99.14	99.70	95.94				
**Proteo-bacteria**	Mean	6.63	21.93	19.62	0.315	NA	NA	NA
	Median	2.08	13.90	9.22				
	Std. Deviation	10.73	24.98	26.66				
	Minimum	0.00	0.00	0.00				
	Maximum	50.65	80.43	92.37				

* The significant p-value is underlined. Percent abundance values that are significantly different from T1 are marked in boldface.

<u>Table S2</u>: List of genera that are significantly differentially abundant in relation to blood-isolated microorganisms

**Genus**	***P* value***
Succiniclasticum	0.014
S5-A14a	0.030
Lawsonella	0.001
UCG-002	0.001
UBA1819	0.016
Peptoniphilus	0.024
Finegoldia	0.036
Finegoldia	0.036
Peptoniphilus	0.024
UBA1819	0.016
UCG-002	0.010
Tyzzerella	0.003
Parasutterella	0.017
Sutterella	0.006
Butyricicoccus	0.037
Lawsonella	0.001
S5-A14a	0.030
Fusicatenibacter	0.004
[Eubacterium] eligens group	0.0001
CAG-352	0.0002
Romboutsia	0.049
UCG-005	0.029
Megasphaera	0.049
Collinsella	0.003
Lachnospiraceae ND3007 group	0.048
Adlercreutzia	0.029
Erysipelotrichaceae UCG-003	0.003
Lachnospira	0.0001
Butyrivibrio	0.002

*Kruskal-Wallis test

<u>Table S3</u>: List of genera that are significantly differentially abundant in patients with blood-stream infection (sepsis)

**Genus**	**Mean in patients with sepsis**	**Mean in patients with negative blood culture**	**Wilcoxon *p value***
Streptococcus	2.04	9.44	0.031
Anaerostipes	3.08	0.17	0.033
[Eubacterium] hallii group	1.53	0.21	0.022
[Clostridium] innocuum group	0.72	0.46	0.026
Dorea	0.54	0.30	0.043

<u>Table S4</u>: List of genera that are significantly differentially abundant in patients with enterocolitis

**Genus**	***P* value***
[*Eubacterium*] *coprostanoligenes*_group	0.0347
Porphyromonas	0.0238
[*Eubacterium*] *hallii* group	0.0296
Christensenellaceae R-7 group	0.0494
Senegalimassilia	0.0364
Barnesiella	0.0364
Collinsella	0.0364
Family_XIII_AD3011_group	0.0152

*Mann-Whitney test

<u>Table S5</u>: List of features that are significantly differentially abundant in patients who received meropenem

**Feature**	***p*-value***
Richness (number of genera)	0.0441
Clostridia_vadinBB60_group	0.0383
Coriobacteriales Incertae Sedis uncultured	0.0363
Romboutsia	0.03
Senegalimassilia	0.0177
Fusicatenibacter	0.0466
Christensenellaceae R-7 group	0.0404
[*Ruminococcus*] *torques* group	0.0190
Ruminococcus	0.0351
[*Eubacterium*] *hallii* group	0.015
Bifidobacterium	0.04
Klebsiella	0.0466
Streptococcus	0.0387

*Mann-Whitney test

Supplementary Figures:

Figure S1: Heatmap-colored correlation matrix between all samples based on their microbial composition by phylum (upper panel) or genus (lower panel)

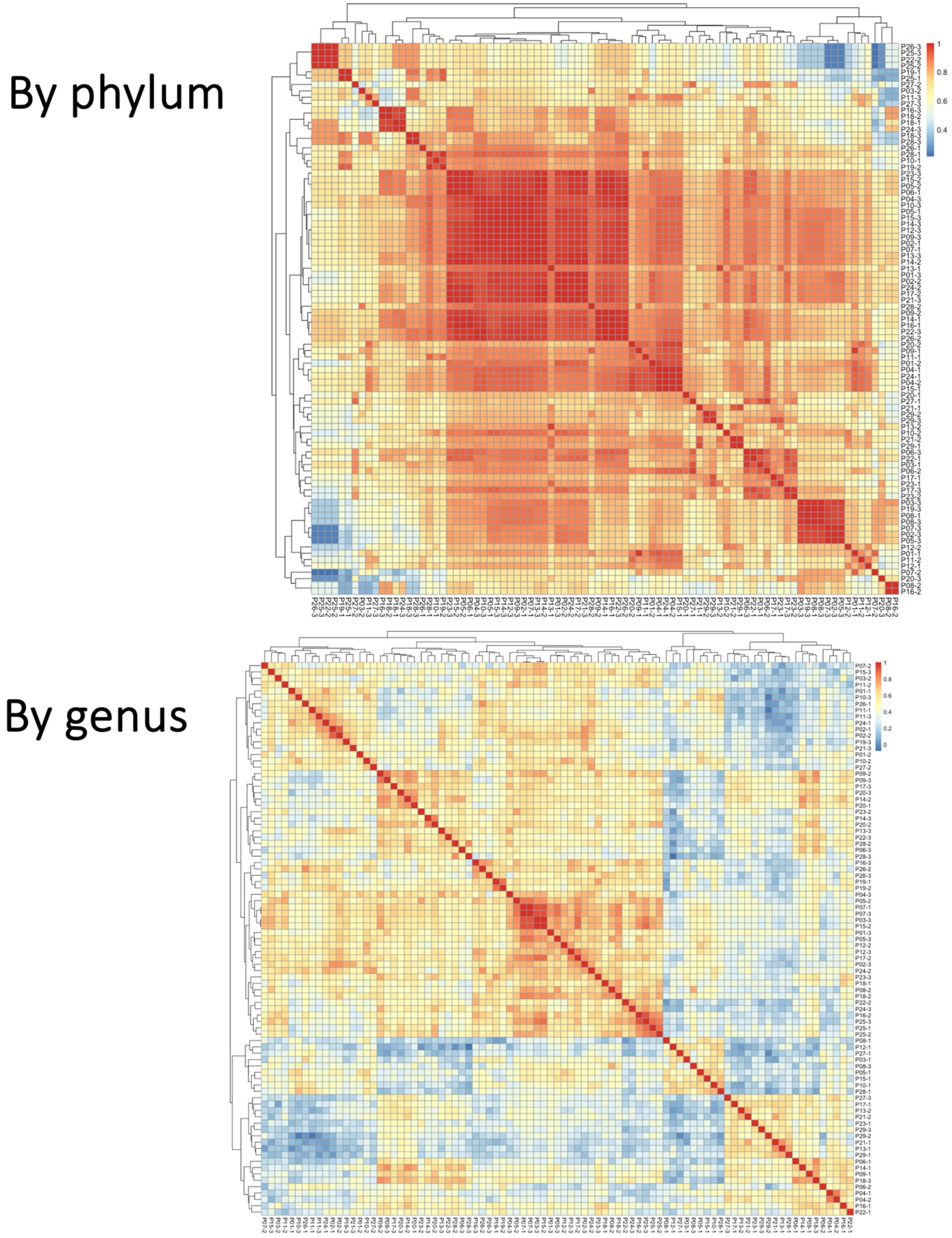

<u>Figure S2</u>: Heatmap-colored correlation plots between major bacterial phyla (upper panel) and genera (lower panel) based on their co-occurrence in different samples.

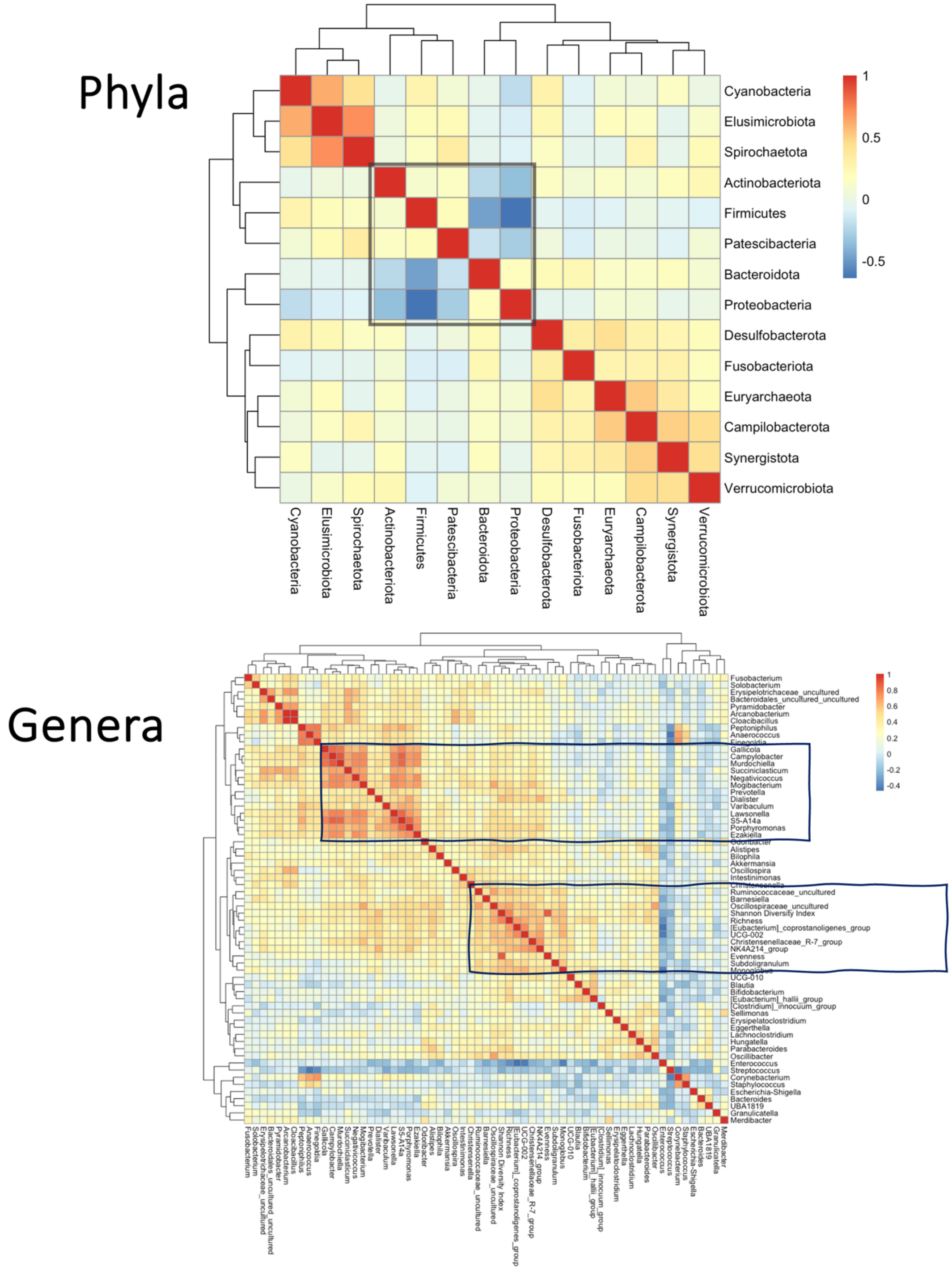

<u>Figure S3</u>: Relation between alpha diversity metrics and stool culture results.

A.

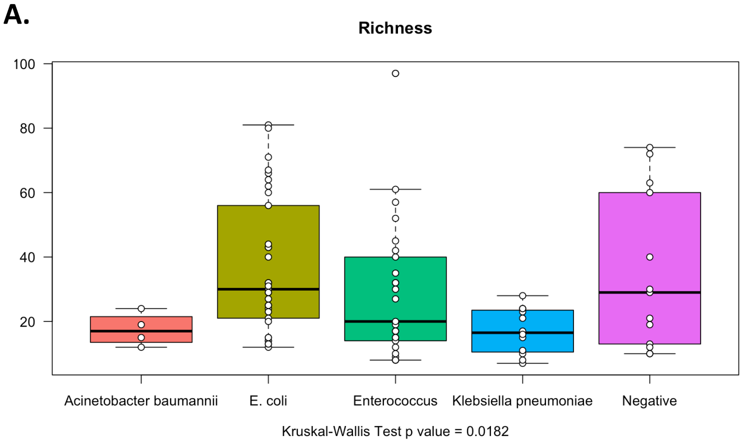

B.

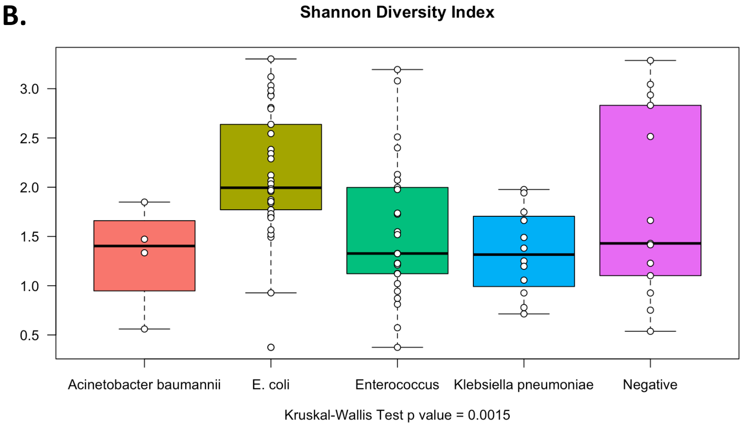

C.

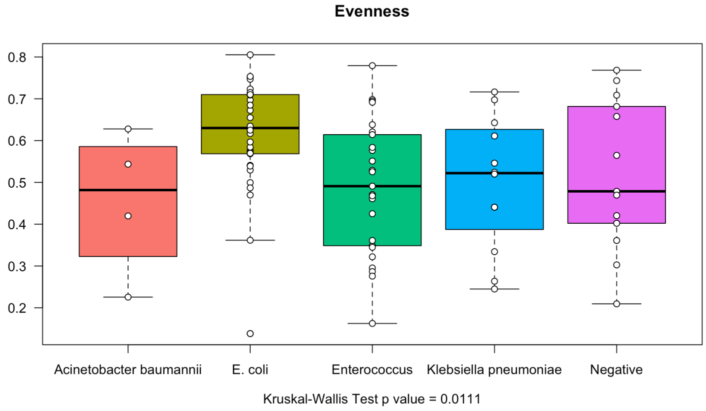

Figure S4: Statistically different features distinguishing patients with and without enterocolitis.

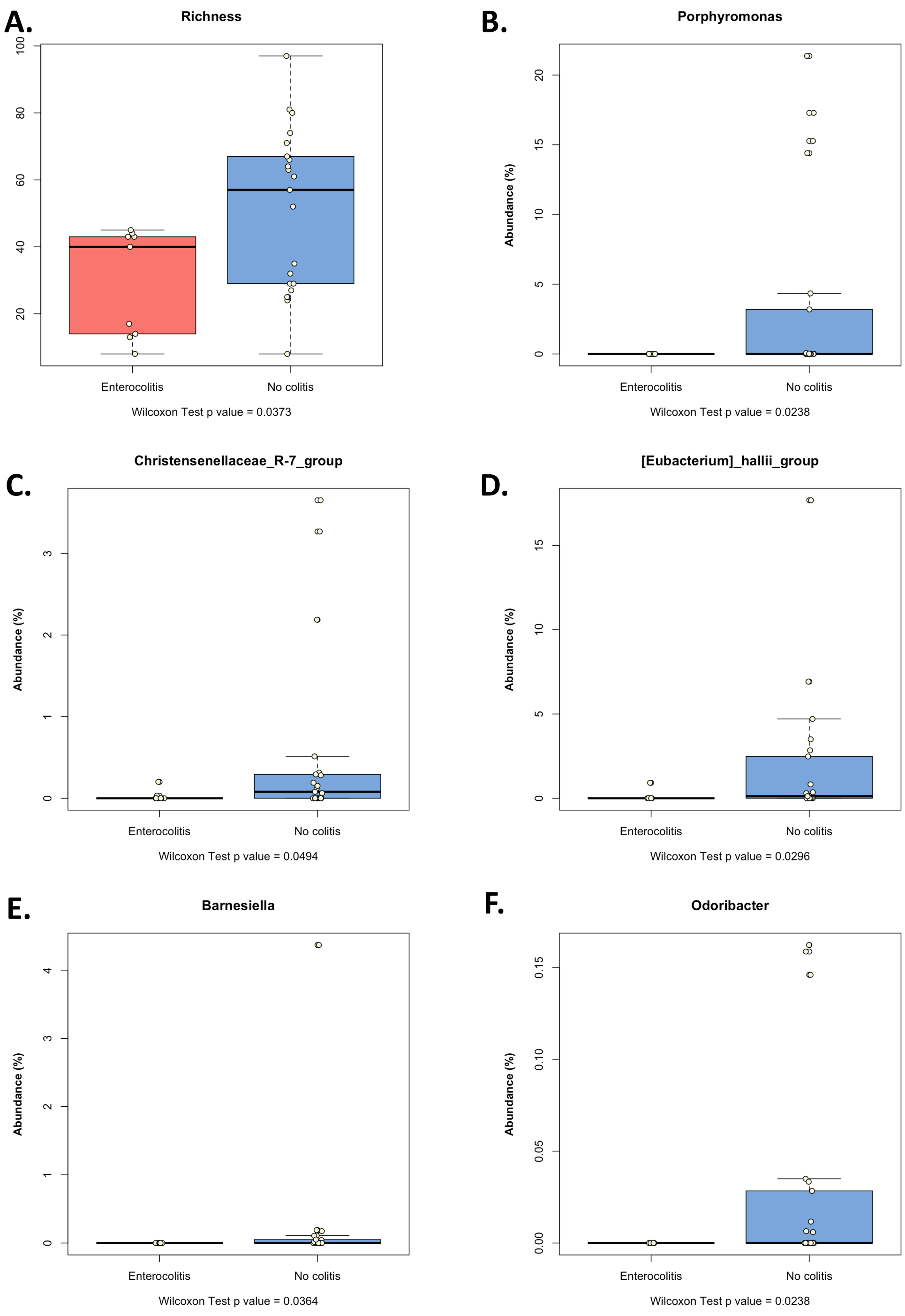

APPENDIX

LINEAR AND LINEAR MIXED MODELS – ANALYSIS IN R

Mai_Adel

- Loading packages and reading the data
  - Select important columns
  - Examine every column
  - Shannon_diversity
  - Linear model
  - Linear mixed model
  - Interaction with age

Loading packages and reading the data

```
library(tidyverse)

library(readxl)

library(flextable)

library(DHARMa)

dat<-read_xlsx("C:/Users/Dr Mohsen/Documents/R/mai_rami/Mai_Adel_Taxa_to_R.xlsx",

               sheet = "Transposed_filtered_genera")

glimpse(dat)
```

```
## Rows: 87
## Columns: 149
## $ Sample
<chr> …
## $ Patient
<chr> …
## $ Group
<chr> …
## $ Chemo
<chr> …
## $ Age
<dbl> …
## $ `Group Description`
<chr> …
## $ Enterocolitis
<chr> …
## $ Stool_Culture
<chr> …
## $ Stool2
<chr> …
## $ Blood
<chr> …
## $ Sepsis
<chr> …
## $ `F-to-B Ratio`
<dbl> …
## $ FvsB
<chr> …
## $ `Shannon Diversity`
<dbl> …
## $ Richness
<dbl> …
## $ Evenness
<dbl> …
## $ P_to_F_Ratio
<dbl> …
## $ `Log2 P-to-F Ratio`
<dbl> …
## $ FvsP
<chr> …
## $ Pair1
<chr> …
## $ Pair2
<chr> …
## $ Pair3
<chr> …
## $ Enterococcus
<dbl> …
## $ Bacteroides
<dbl> …
## $ `Escherichia-Shigella`
<dbl> …
## $ Streptococcus
<dbl> …
## $ Klebsiella
```

```
<dbl> …
## $ Anaerococcus
<dbl> …
## $ Corynebacterium
<dbl> …
## $ Finegoldia
<dbl> …
## $ Faecalibacterium
<dbl> …
## $ Bifidobacterium
<dbl> …
## $ Lactobacillus
<dbl> …
## $ Peptoniphilus
<dbl> …
## $ Prevotella
<dbl> …
## $ Subdoligranulum
<dbl> …
## $ Enterobacter
<dbl> …
## $ Veillonella
<dbl> …
## $ `[Ruminococcus]_gnavus_group`
<dbl> …
## $ Parabacteroides
<dbl> …
## $ Blautia
<dbl> …
## $ Anaerostipes
<dbl> …
## $ `[Eubacterium]_coprostanoligenes_group`
<dbl> …
## $ Akkermansia
<dbl> …
## $ Porphyromonas
<dbl> …
## $ Staphylococcus
<dbl> …
## $ Agathobacter
<dbl> …
## $ Alistipes
<dbl> …
## $ Erysipelatoclostridium
<dbl> …
## $ `[Eubacterium]_hallii_group`
<dbl> …
## $ Ezakiella
<dbl> …
## $ UBA1819
<dbl> …
## $ `[Clostridium]_innocuum_group`
<dbl> …
## $ Fenollaria
<dbl> …
## $ Lachnoclostridium
```

```
<dbl> …
## $ Catabacter
<dbl> …
## $ Dorea
<dbl> …
## $ `UCG-002`
<dbl> …
## $ Dialister
<dbl> …
## $ Acinetobacter
<dbl> …
## $ Megamonas
<dbl> …
## $ Clostridium_sensu_stricto_1
<dbl> …
## $ Tyzzerella
<dbl> …
## $ Parasutterella
<dbl> …
## $ Holdemanella
<dbl> …
## $ Alloprevotella
<dbl> …
## $ Eggerthella
<dbl> …
## $ Ruminococcus
<dbl> …
## $ `[Ruminococcus]_torques_group`
<dbl> …
## $ Campylobacter
<dbl> …
## $ Actinomyces
<dbl> …
## $ Phascolarctobacterium
<dbl> …
## $ Murdochiella
<dbl> …
## $ Haemophilus
<dbl> …
## $ Monoglobus
<dbl> …
## $ Sutterella
<dbl> …
## $ Intestinibacter
<dbl> …
## $ Peptostreptococcus
<dbl> …
## $ Butyricicoccus
<dbl> …
## $ `Christensenellaceae_R-7_group`
<dbl> …
## $ Bilophila
<dbl> …
## $ Lawsonella
<dbl> …
## $ `S5-A14a`
```

```
<dbl> …
## $ Fusicatenibacter
<dbl> …
## $ `[Eubacterium]_eligens_group`
<dbl> …
## $ Granulicatella
<dbl> …
## $ Senegalimassilia
<dbl> …
## $ Roseburia
<dbl> …
## $ Flavonifractor
<dbl> …
## $ Varibaculum
<dbl> …
## $ `[Bacteroides]_pectinophilus_group`
<dbl> …
## $ Mobiluncus
<dbl> …
## $ Muribaculaceae
<dbl> …
## $ `CAG-352`
<dbl> …
## $ Romboutsia
<dbl> …
## $ d__Bacteria_Firmicutes_Clostridia_Oscillospirales_Ruminococcaceae_uncultured
<dbl> …
## $ Coprococcus
<dbl> …
## $ Gallicola
<dbl> …
## $ `Clostridia_UCG-014`
<dbl> …
## $ `UCG-005`
<dbl> …
## $ Merdibacter
<dbl> …
## $ Barnesiella
<dbl> …
## $ Weissella
<dbl> …
## $ Fastidiosipila
<dbl> …
## $ Megasphaera
<dbl> …
## $ Peptococcus
<dbl> …
## $ Methanobrevibacter
<dbl> …
## $ Hungatella
<dbl> …
## $ d__Bacteria_Firmicutes_Clostridia_Oscillospirales_Oscillospiraceae_uncultured
<dbl> …
## $ `[Ruminococcus]_gauvreauii_group`
<dbl> …
## $ Collinsella
```

```
<dbl> …
## $ Fusobacterium
<dbl> …
## $ Clostridioides
<dbl> …
## $ Sellimonas
<dbl> …
## $ Rothia
<dbl> …
## $ Lachnospiraceae_ND3007_group
<dbl> …
## $ Clostridium_sensu_stricto_16
<dbl> …
## $ Negativicoccus
<dbl> …
## $ Adlercreutzia
<dbl> …
## $ d__Bacteria_Proteobacteria_Alphaproteobacteria_Rhodospirillales_uncultured_uncul
tured                   <dbl> …
## $ Succinivibrio
<dbl> …
## $ Family_XIII_AD3011_group
<dbl> …
## $ Oscillibacter
<dbl> …
## $ Pyramidobacter
<dbl> …
## $ `UCG-004`
<dbl> …
## $ Rikenellaceae_RC9_gut_group
<dbl> …
## $ `Erysipelotrichaceae_UCG-003`
<dbl> …
## $ d__Bacteria_Firmicutes_Bacilli_Erysipelotrichales_Erysipelotrichaceae_uncultured
<dbl> …
## $ Lachnospira
<dbl> …
## $ Solobacterium
<dbl> …
## $ Butyrivibrio
<dbl> …
## $ d__Bacteria_Actinobacteriota_Coriobacteriia_Coriobacteriales_Coriobacteriales_In
certae_Sedis_uncultured <dbl> …
## $ Faecalitalea
<dbl> …
## $ Clostridia_vadinBB60_group
<dbl> …
## $ d__Bacteria_Firmicutes_Clostridia_Oscillospirales_Butyricicoccaceae_uncultured
<dbl> …
## $ Helcococcus
<dbl> …
## $ Therapeutic_Antibiotic
<chr> …
## $ Before_Chemo
<chr> …
## $ Tazocin
```

```
<chr> …
## $ Maxipime
<chr> …
## $ Zithromax
<chr> …
## $ Meropenem
<chr> …
## $ Amikacin
<chr> …
## $ Vancomycin
<chr> …
## $ Meropenem_high_dose
<chr> …
## $ Colistin
<chr> …
## $ Tigecycline
<chr> …
## $ Meropenem_Tazocin
<chr> …
## $ Mero_Tazo
<chr> …
```

The data is 87 rows and 149 columns.

Select important columns

```
library(janitor)

dat2<-dat %>% select(2:19,137:149) %>% clean_names()

glimpse(dat2)
```

```
## Rows: 87
## Columns: 31
## $ patient                <chr> "P01", "P01", "P01", "P02", "P02", "P02", "P03"…
## $ group                  <chr> "T1", "T2", "T3", "T1", "T2", "T3", "T1", "T2",…
## $ chemo                  <chr> "No", "Yes", "Yes", "No", "Yes", "Yes", "No", "…
## $ age                    <dbl> 10, 10, 10, 2, 2, 2, 13, 13, 13, 6, 6, 6, 2, 2,…
## $ group_description      <chr> "Before treatment", "Week 1", "Week 4", "Before…
## $ enterocolitis          <chr> "Yes", NA, NA, "No", NA, NA, "No", NA, NA, "No"…
## $ stool_culture          <chr> "E. coli", "Acinetobacter baumannii", "Acinetob…
## $ stool2                 <chr> "E. coli", "Acinetobacter baumannii", "Acinetob…
## $ blood                  <chr> "Staphylococcus", "Acinetobacter baumannii", "K…
## $ sepsis                 <chr> "Yes", "Yes", "Yes", "No", "No", "No", "No", "N…
## $ f_to_b_ratio           <dbl> 5.941105e-01, 3.375436e-01, 8.519637e-02, 2.321…
## $ fvs_b                  <chr> "B>F", "B>F", "B>F", "F>B", "B>F", "B>F", "F>B"…
## $ shannon_diversity      <dbl> 2.3826907, 1.3340824, 0.5605462, 2.1216051, 1.7…
## $ richness               <dbl> 43, 24, 12, 29, 28, 21, 57, 32, 12, 66, 40, 15,…
## $ evenness               <dbl> 0.6334921, 0.4197797, 0.2255804, 0.6300620, 0.5…
## $ p_to_f_ratio           <dbl> 6.424825e-01, 1.024196e+01, 1.551348e+02, 3.475…
## $ log2_p_to_f_ratio      <dbl> -0.6382709, 3.3564201, 7.2773781, -1.5247160, 2…
## $ fvs_p                  <chr> "F>P", "P>F", "P>F", "F>P", "P>F", "P>F", "F>P"…
## $ therapeutic_antibiotic <chr> "yes", "yes", "yes", "yes", "yes", "yes", "yes"…
## $ before_chemo           <chr> "yes", "no", "no", "no", "no", "no", "no", "no"…
## $ tazocin                <chr> "no", "no", "no", "no", "no", "no", "no", "no",…
## $ maxipime               <chr> "yes", "no", "no", "no", "yes", "no", "yes", "n…
## $ zithromax              <chr> "no", "no", "no", "no", "no", "no", "no", "no",…
## $ meropenem              <chr> "yes", "yes", "yes", "yes", "yes", "yes", "no",…
## $ amikacin               <chr> "yes", "no", "no", "yes", "yes", "yes", "no", "…
## $ vancomycin             <chr> "yes", "yes", "yes", "yes", "yes", "yes", "yes"…
## $ meropenem_high_dose    <chr> "yes", "yes", "yes", "no", "no", "no", "no", "n…
## $ colistin               <chr> "yes", "yes", "yes", "no", "no", "no", "no", "n…
## $ tigecycline            <chr> "yes", "yes", "yes", "no", "no", "no", "no", "n…
## $ meropenem_tazocin      <chr> "M", "M", "M", "M", "M", "M", "neither", "M", "…
## $ mero_tazo              <chr> "both", "both", "both", "no", "no", "no", "no",…
```

Examine every column

patient

```
dat2 %>% count(patient, sort = T)
```

```
## # A tibble: 29 × 2
##    patient     n
##    <chr>   <int>
##  1 P01         3
##  2 P02         3
##  3 P03         3
##  4 P04         3
##  5 P05         3
##  6 P06         3
##  7 P07         3
##  8 P08         3
##  9 P09         3
## 10 P10         3
## # ℹ 19 more rows
```

We have 29 patients.

group (time point)

```
dat2 %>% count(group, sort = T)
```

```
## # A tibble: 3 × 2
##   group     n
##   <chr> <int>
## 1 T1       29
## 2 T2       29
## 3 T3       29
```

We have 3 time points.

chemo

```
dat2 %>% count(chemo, sort = T)
```

```
## # A tibble: 2 × 2
##   chemo     n
##   <chr> <int>
## 1 Yes      58
## 2 No       29
```

We have 2 chemotherapy groups.

age

```
dat2 %>% count(age, sort = T)
```

```
## # A tibble: 16 × 2
##      age     n
##    <dbl> <int>
##  1   2      15
##  2   5       9
##  3  10       9
##  4   3       6
##  5   7       6
##  6   9       6
##  7  11       6
##  8  13       6
##  9   1.5     3
## 10   4       3
## 11   6       3
## 12   8       3
## 13  15       3
## 14  16       3
## 15  17       3
## 16  18       3
```

enterocolitis

```
dat2 %>% count(enterocolitis, sort = T)
```

```
## # A tibble: 3 × 2
##   enterocolitis     n
##   <chr>         <int>
## 1 <NA>             57
## 2 No               21
## 3 Yes               9
```

```
dat2 %>% count(patient, enterocolitis) %>% drop_na()
```

```
## # A tibble: 29 × 3
##    patient enterocolitis     n
##    <chr>   <chr>         <int>
##  1 P01     Yes               1
##  2 P02     No                1
##  3 P03     No                1
##  4 P04     No                1
##  5 P05     No                1
##  6 P06     Yes               1
##  7 P07     No                1
##  8 P08     No                1
##  9 P09     No                1
## 10 P10     No                1
## # ℹ 19 more rows
```

```
dat2_entero<-dat2 %>% count(patient, enterocolitis) %>% drop_na() %>%

  select(-n)

dat2_entero
```

```
## # A tibble: 29 × 2
##    patient enterocolitis
##    <chr>   <chr>
##  1 P01     Yes
##  2 P02     No
##  3 P03     No
##  4 P04     No
##  5 P05     No
##  6 P06     Yes
##  7 P07     No
##  8 P08     No
##  9 P09     No
## 10 P10     No
## # ℹ 19 more rows
```

Generate a new data

```
dat3<-dat2 %>% full_join(dat2_entero, by="patient") %>%

  select(-enterocolitis.x,-group_description) %>%

  rename(enterocolitis = enterocolitis.y)

glimpse(dat3)
```

```
## Rows: 87
## Columns: 30
## $ patient                 <chr> "P01", "P01", "P01", "P02", "P02", "P02", "P03"…
## $ group                   <chr> "T1", "T2", "T3", "T1", "T2", "T3", "T1", "T2",…
## $ chemo                   <chr> "No", "Yes", "Yes", "No", "Yes", "Yes", "No", "…
## $ age                     <dbl> 10, 10, 10, 2, 2, 2, 13, 13, 13, 6, 6, 6, 2, 2,…
## $ stool_culture           <chr> "E. coli", "Acinetobacter baumannii", "Acinetob…
## $ stool2                  <chr> "E. coli", "Acinetobacter baumannii", "Acinetob…
## $ blood                   <chr> "Staphylococcus", "Acinetobacter baumannii", "K…
## $ sepsis                  <chr> "Yes", "Yes", "Yes", "No", "No", "No", "No", "N…
## $ f_to_b_ratio            <dbl> 5.941105e-01, 3.375436e-01, 8.519637e-02, 2.321…
## $ fvs_b                   <chr> "B>F", "B>F", "B>F", "F>B", "B>F", "B>F", "F>B"…
## $ shannon_diversity       <dbl> 2.3826907, 1.3340824, 0.5605462, 2.1216051, 1.7…
## $ richness                <dbl> 43, 24, 12, 29, 28, 21, 57, 32, 12, 66, 40, 15,…
## $ evenness                <dbl> 0.6334921, 0.4197797, 0.2255804, 0.6300620, 0.5…
## $ p_to_f_ratio            <dbl> 6.424825e-01, 1.024196e+01, 1.551348e+02, 3.475…
## $ log2_p_to_f_ratio       <dbl> -0.6382709, 3.3564201, 7.2773781, -1.5247160, 2…
## $ fvs_p                   <chr> "F>P", "P>F", "P>F", "F>P", "P>F", "P>F", "F>P"…
## $ therapeutic_antibiotic <chr> "yes", "yes", "yes", "yes", "yes", "yes", "yes"…
## $ before_chemo            <chr> "yes", "no", "no", "no", "no", "no", "no", "no"…
## $ tazocin                 <chr> "no", "no", "no", "no", "no", "no", "no", "no",…
## $ maxipime                <chr> "yes", "no", "no", "no", "yes", "no", "yes", "n…
## $ zithromax               <chr> "no", "no", "no", "no", "no", "no", "no", "no",…
## $ meropenem               <chr> "yes", "yes", "yes", "yes", "yes", "yes", "no",…
## $ amikacin                <chr> "yes", "no", "no", "yes", "yes", "yes", "no", "…
## $ vancomycin              <chr> "yes", "yes", "yes", "yes", "yes", "yes", "yes"…
## $ meropenem_high_dose     <chr> "yes", "yes", "yes", "no", "no", "no", "no", "n…
## $ colistin                <chr> "yes", "yes", "yes", "no", "no", "no", "no", "n…
## $ tigecycline             <chr> "yes", "yes", "yes", "no", "no", "no", "no", "n…
## $ meropenem_tazocin       <chr> "M", "M", "M", "M", "M", "M", "neither", "M", "…
## $ mero_tazo               <chr> "both", "both", "both", "no", "no", "no", "no",…
## $ enterocolitis           <chr> "Yes", "Yes", "Yes", "No", "No", "No", "No", "N…
```

The data is 87 rows and 30 columns.

stool_culture

```
dat3 %>% count(stool_culture, sort = T)
```

```
## # A tibble: 5 × 2
##   stool_culture               n
##   <chr>                   <int>
## 1 E. coli                    33
## 2 Enterococcus               25
## 3 Negative                   13
## 4 Klebsiella pneumoniae      12
## 5 Acinetobacter baumannii     4
```

stool2

```
dat3 %>% count(stool2, sort = T)
```

```
## # A tibble: 4 × 2
##   stool2                       n
##   <chr>                    <int>
## 1 E. coli                     44
## 2 Negative                    26
## 3 Klebsiella pneumoniae       14
## 4 Acinetobacter baumannii      3
```

blood

```
dat3 %>% count(blood, sort = T)
```

```
## # A tibble: 7 × 2
##   blood                        n
##   <chr>                    <int>
## 1 Negative                    57
## 2 Klebsiella pneumoniae       14
## 3 Staphylococcus               8
## 4 E. coli                      4
## 5 Acinetobacter baumannii      2
## 6 MRSA                         1
## 7 Streptococcus mitis          1
```

sepsis

```
dat3 %>% count(sepsis, sort = T)
```

```
## # A tibble: 2 × 2
##   sepsis     n
##   <chr>  <int>
## 1 No        57
## 2 Yes       30
```

```
dat3 %>% count(patient, sepsis)
```

```
## # A tibble: 37 × 3
##    patient sepsis     n
##    <chr>   <chr>  <int>
##  1 P01     Yes        3
##  2 P02     No         3
##  3 P03     No         3
##  4 P04     No         2
##  5 P04     Yes        1
##  6 P05     No         3
##  7 P06     No         2
##  8 P06     Yes        1
##  9 P07     No         2
## 10 P07     Yes        1
## # ℹ 27 more rows
```

Some patients developed sepsis over time.

f_to_b_ratio

```
dat3 %>% count(f_to_b_ratio, sort = T)
```

```
## # A tibble: 78 × 2
##    f_to_b_ratio     n
##           <dbl> <int>
##  1      NA         10
##  2       0.0852     1
##  3       0.105      1
##  4       0.297      1
##  5       0.299      1
##  6       0.338      1
##  7       0.374      1
##  8       0.457      1
##  9       0.472      1
## 10       0.527      1
## # ℹ 68 more rows
```

fvs_b

```
dat3 %>% count(fvs_b)
```

```
## # A tibble: 2 × 2
##   fvs_b     n
##   <chr> <int>
## 1 B>F      13
## 2 F>B      74
```

fvs_p

```
dat3 %>% count(fvs_p)
```

```
## # A tibble: 2 × 2
##   fvs_p     n
##   <chr> <int>
## 1 F>P      71
## 2 P>F      16
```

shannon_diversity

```
dat3 %>% count(shannon_diversity, sort = T)
```

```
## # A tibble: 87 × 2
##    shannon_diversity     n
##                <dbl> <int>
##  1             0.374     1
##  2             0.374     1
##  3             0.537     1
##  4             0.561     1
##  5             0.573     1
##  6             0.714     1
##  7             0.752     1
##  8             0.778     1
##  9             0.812     1
## 10             0.872     1
## # ℹ 77 more rows
```

```
summary(dat3$shannon_diversity)
```

```
##    Min. 1st Qu.  Median    Mean 3rd Qu.    Max. 
##  0.3742  1.2268  1.7310  1.7769  2.2085  3.3017
```

richness

```
dat3 %>% count(richness, sort = T)
```

```
## # A tibble: 45 × 2
##    richness     n
##       <dbl> <int>
##  1       15     6
##  2        8     4
##  3       10     4
##  4       12     4
##  5       24     4
##  6       32     4
##  7       13     3
##  8       14     3
##  9       17     3
## 10       19     3
## # ℹ 35 more rows
```

```
summary(dat3$richness)
```

```
##    Min. 1st Qu.  Median    Mean 3rd Qu.    Max. 
##    7.00   15.00   24.00   30.69   41.00   97.00
```

evenness

```
dat3 %>% count(evenness, sort = T)
```

```
## # A tibble: 87 × 2
##    evenness     n
##       <dbl> <int>
##  1    0.138     1
##  2    0.163     1
##  3    0.209     1
##  4    0.226     1
##  5    0.245     1
##  6    0.264     1
##  7    0.276     1
##  8    0.287     1
##  9    0.295     1
## 10    0.303     1
## # ℹ 77 more rows
```

```
summary(dat3$evenness)
```

```
##    Min. 1st Qu.  Median    Mean 3rd Qu.    Max.
##  0.1383  0.4406  0.5684  0.5403  0.6770  0.8053
```

p_to_f_ratio

```
dat3 %>% count(p_to_f_ratio, sort = T)
```

```
## # A tibble: 78 × 2
##    p_to_f_ratio     n
##           <dbl> <int>
##  1    0            10
##  2    0.0000617     1
##  3    0.0000783     1
##  4    0.000287      1
##  5    0.000461      1
##  6    0.000752      1
##  7    0.00113       1
##  8    0.00206       1
##  9    0.00313       1
## 10    0.00363       1
## # ℹ 68 more rows
```

```
summary(dat3$p_to_f_ratio)
```

```
##      Min.   1st Qu.    Median      Mean   3rd Qu.      Max.
##   0.00000   0.00631   0.03874   2.39132   0.43251 155.13475
```

log2_p_to_f_ratio

```
dat3 %>% count(log2_p_to_f_ratio, sort = T)
```

```
## # A tibble: 78 × 2
##    log2_p_to_f_ratio     n
##                <dbl> <int>
##  1             NA       10
##  2            -14.0      1
##  3            -13.6      1
##  4            -11.8      1
##  5            -11.1      1
##  6            -10.4      1
##  7             -9.79     1
##  8             -8.93     1
##  9             -8.32     1
## 10             -8.11     1
## # ℹ 68 more rows
```

```
summary(dat3$log2_p_to_f_ratio)
```

```
##     Min.  1st Qu.   Median     Mean  3rd Qu.     Max.     NA's 
## -13.9845  -6.1236  -3.5795  -3.5969  -0.6383   7.2774       10
```

The 0 value transformed to NA.

therapeutic_antibiotic

```
dat3 %>% count(therapeutic_antibiotic, sort = T)
```

```
## # A tibble: 2 × 2
##   therapeutic_antibiotic     n
##   <chr>                  <int>
## 1 yes                       86
## 2 no                         1
```

This column is not useful in analysis.

before_chemo

```
dat3 %>% count(before_chemo, sort = T)
```

```
## # A tibble: 2 × 2
##   before_chemo     n
##   <chr>        <int>
## 1 no              83
## 2 yes              4
```

tazocin

```
dat3 %>% count(tazocin, sort = T)
```

```
## # A tibble: 2 × 2
##   tazocin     n
##   <chr>   <int>
## 1 no         71
## 2 yes        16
```

maxipime

```
dat3 %>% count(maxipime, sort = T)
```

```
## # A tibble: 2 × 2
##   maxipime     n
##   <chr>    <int>
## 1 no          51
## 2 yes         36
```

zithromax

```
dat3 %>% count(zithromax, sort = T)
```

```
## # A tibble: 2 × 2
##   zithromax     n
##   <chr>     <int>
## 1 no           85
## 2 yes           2
```

meropenem

```
dat3 %>% count(meropenem)
```

```
## # A tibble: 2 × 2
##   meropenem     n
##   <chr>     <int>
## 1 no           25
## 2 yes          62
```

meropenem_high_dose

```
dat3 %>% count(meropenem, meropenem_high_dose, meropenem_tazocin, mero_tazo)
```

```
## # A tibble: 6 × 5
##   meropenem meropenem_high_dose meropenem_tazocin mero_tazo     n
##   <chr>     <chr>               <chr>             <chr>     <int>
## 1 no        no                  T                 no            5
## 2 no        no                  neither           no           20
## 3 yes       no                  Both              no            7
## 4 yes       no                  M                 no           43
## 5 yes       yes                 Both              both          4
## 6 yes       yes                 M                 both          8
```

Are there some mistakes?

amikacin

```
dat3 %>% count(amikacin)
```

```
## # A tibble: 2 × 2
##   amikacin     n
##   <chr>    <int>
## 1 no          40
## 2 yes         47
```

vancomycin

```
dat3 %>% count(vancomycin)
```

```
## # A tibble: 2 × 2
##   vancomycin     n
##   <chr>      <int>
## 1 no            56
## 2 yes           31
```

colistin

```
dat3 %>% count(colistin)
```

```
## # A tibble: 2 × 2
##   colistin     n
##   <chr>    <int>
## 1 no          81
## 2 yes          6
```

tigecycline

```
dat3 %>% count(tigecycline)
```

```
## # A tibble: 2 × 2
##   tigecycline     n
##   <chr>       <int>
## 1 no             78
## 2 yes             9
```

Shannon_diversity

Linear model

```
mod<-lm(shannon_diversity ~ group+chemo+age, data = dat3)

summary(mod)
```

```
##
## Call:
## lm(formula = shannon_diversity ~ group + chemo + age, data = dat3)
##
## Residuals:
##      Min       1Q   Median       3Q      Max
## -1.40927 -0.49616  0.02943  0.43018  1.85588
##
## Coefficients: (1 not defined because of singularities)
##             Estimate Std. Error t value Pr(>|t|)
## (Intercept)  1.88803    0.17040  11.080  < 2e-16 ***
## groupT2     -0.59232    0.17731  -3.341 0.001255 **
## groupT3     -0.65238    0.17731  -3.679 0.000415 ***
## chemoYes          NA         NA      NA       NA
## age          0.03890    0.01477   2.633 0.010098 *
## ---
## Signif. codes:  0 '***' 0.001 '**' 0.01 '*' 0.05 '.' 0.1 ' ' 1
##
## Residual standard error: 0.6752 on 83 degrees of freedom
## Multiple R-squared:  0.2205, Adjusted R-squared:  0.1923
## F-statistic: 7.824 on 3 and 83 DF,  p-value: 0.0001163
```

- chemo is correlated with time factor so cannot be estimated together, we will use chemo column only.

```
mod<-lm(shannon_diversity ~ chemo+age+enterocolitis+

          stool_culture+ stool2+

          sepsis+ tazocin+ maxipime+ zithromax+meropenem+

          amikacin+vancomycin+colistin+tigecycline, data = dat3)

summary(mod)
```

```
##
## Call:
## lm(formula = shannon_diversity ~ chemo + age + enterocolitis +
##     stool_culture + stool2 + sepsis + tazocin + maxipime + zithromax +
##     meropenem + amikacin + vancomycin + colistin + tigecycline,
##     data = dat3)
##
## Residuals:
##     Min      1Q  Median      3Q     Max
## -1.2958 -0.3713  0.0000  0.3857  1.5543
##
## Coefficients:
##                                     Estimate Std. Error t value Pr(>|t|)
## (Intercept)                          1.58362    0.59219   2.674   0.0094 **
## chemoYes                            -0.46644    0.19353  -2.410   0.0187 *
## age                                  0.02866    0.01747   1.641   0.1056
## enterocolitisYes                    -0.37536    0.18627  -2.015   0.0479 *
## stool_cultureE. coli                -0.10492    0.99626  -0.105   0.9164
## stool_cultureEnterococcus           -0.20944    1.02516  -0.204   0.8387
## stool_cultureKlebsiella pneumoniae -0.25126    0.71918  -0.349   0.7279
## stool_cultureNegative               -0.24194    0.96293  -0.251   0.8024
## stool2E. coli                        0.61394    1.11409   0.551   0.5834
## stool2Klebsiella pneumoniae          0.18281    0.84344   0.217   0.8291
## stool2Negative                       0.34480    1.13235   0.304   0.7617
## sepsisYes                            0.08221    0.17700   0.464   0.6438
## tazocinyes                           0.04915    0.21770   0.226   0.8221
## maxipimeyes                          0.10970    0.17597   0.623   0.5351
## zithromaxyes                         0.43577    0.52110   0.836   0.4060
## meropenemyes                         0.09921    0.22847   0.434   0.6655
## amikacinyes                         -0.03522    0.19108  -0.184   0.8543
## vancomycinyes                       -0.07724    0.16883  -0.458   0.6488
## colistinyes                          0.53383    0.53560   0.997   0.3225
## tigecyclineyes                      -0.40537    0.47506  -0.853   0.3965
## ---
## Signif. codes:  0 '***' 0.001 '**' 0.01 '*' 0.05 '.' 0.1 ' ' 1
##
## Residual standard error: 0.6611 on 67 degrees of freedom
## Multiple R-squared:  0.3968, Adjusted R-squared:  0.2257
## F-statistic:  2.32 on 19 and 67 DF,  p-value: 0.006137
```

Model diagnostics

```
simulationOutput <- simulateResiduals(mod, plot = F)

plot(simulationOutput)
```

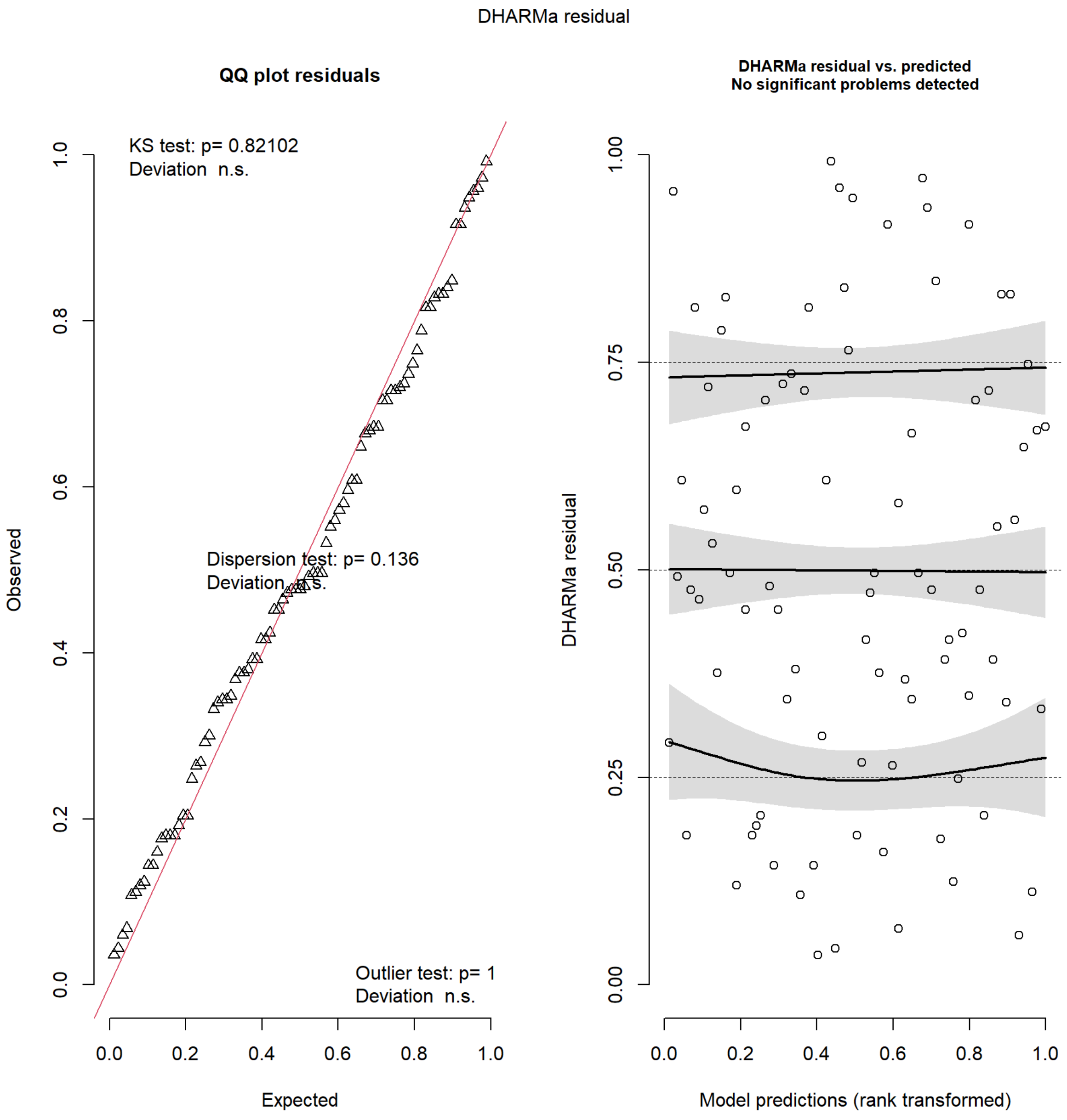

- The model is ok.

Linear mixed model

```
library(lme4)

library(lmerTest)

library(broom.mixed)

mod1<-lmer(shannon_diversity ~ chemo+age+enterocolitis+

          stool_culture+ stool2+

          sepsis+ tazocin+ maxipime+ zithromax+meropenem+

          amikacin+vancomycin+colistin+tigecycline+

            (1|patient), data = dat3)

summary(mod1)
```

```
## Linear mixed model fit by REML. t-tests use Satterthwaite's method [
## lmerModLmerTest]
## Formula: shannon_diversity ~ chemo + age + enterocolitis + stool_culture +
##     stool2 + sepsis + tazocin + maxipime + zithromax + meropenem +
##     amikacin + vancomycin + colistin + tigecycline + (1 | patient)
##    Data: dat3
##
## REML criterion at convergence: 175.3
##
## Scaled residuals:
##     Min      1Q  Median      3Q     Max
## -1.5716 -0.6571  0.0000  0.5065  1.8174
##
## Random effects:
##  Groups   Name        Variance Std.Dev.
##  patient  (Intercept) 0.1735   0.4165
##  Residual             0.2983   0.5462
## Number of obs: 87, groups:  patient, 29
##
## Fixed effects:
##                                 Estimate Std. Error        df t value
## (Intercept)                     1.618620   0.571621 66.711218   2.832
## chemoYes                       -0.505874   0.172955 54.851250  -2.925
## age                             0.028381   0.022457 21.531298   1.264
## enterocolitisYes               -0.468636   0.241791 20.856325  -1.938
## stool_cultureE. coli            0.637216   0.912659 54.961067   0.698
## stool_cultureEnterococcus       0.593909   0.952910 57.891781   0.623
## stool_cultureKlebsiella pneumoniae  0.098925   0.667162 57.284878   0.148
## stool_cultureNegative           0.497278   0.896676 57.500476   0.555
## stool2E. coli                  -0.202719   1.025814 56.178760  -0.198
## stool2Klebsiella pneumoniae    -0.201387   0.778306 56.790736  -0.259
## stool2Negative                 -0.300666   1.050512 57.320075  -0.286
## sepsisYes                      -0.002143   0.202049 46.843102  -0.011
## tazocinyes                      0.105436   0.241480 51.911516   0.437
## maxipimeyes                     0.201626   0.167781 62.565992   1.202
## zithromaxyes                    0.098477   0.498764 63.684682   0.197
## meropenemyes                    0.074578   0.204365 53.024722   0.365
## amikacinyes                    -0.036761   0.171096 52.553743  -0.215
## vancomycinyes                  -0.056855   0.179827 63.912552  -0.316
## colistinyes                     1.132065   0.566928 62.861439   1.997
## tigecyclineyes                 -0.655486   0.467214 66.492265  -1.403
##                                Pr(>|t|)
## (Intercept)                     0.00612 **
## chemoYes                        0.00500 **
## age                             0.21981
## enterocolitisYes                0.06626 .
## stool_cultureE. coli            0.48800
## stool_cultureEnterococcus       0.53556
## stool_cultureKlebsiella pneumoniae  0.88264
## stool_cultureNegative           0.58133
## stool2E. coli                   0.84406
## stool2Klebsiella pneumoniae     0.79676
## stool2Negative                  0.77575
## sepsisYes                       0.99158
## tazocinyes                      0.66420
```

```
## maxipimeyes                        0.23400
## zithromaxyes                       0.84411
## meropenemyes                       0.71662
## amikacinyes                        0.83071
## vancomycinyes                      0.75291
## colistinyes                        0.05018 .
## tigecyclineyes                     0.16528
## ---
## Signif. codes:  0 '***' 0.001 '**' 0.01 '*' 0.05 '.' 0.1 ' ' 1
```

Model diagnostics

```
simulationOutput <- simulateResiduals(mod1, plot = F)

plot(simulationOutput)
```

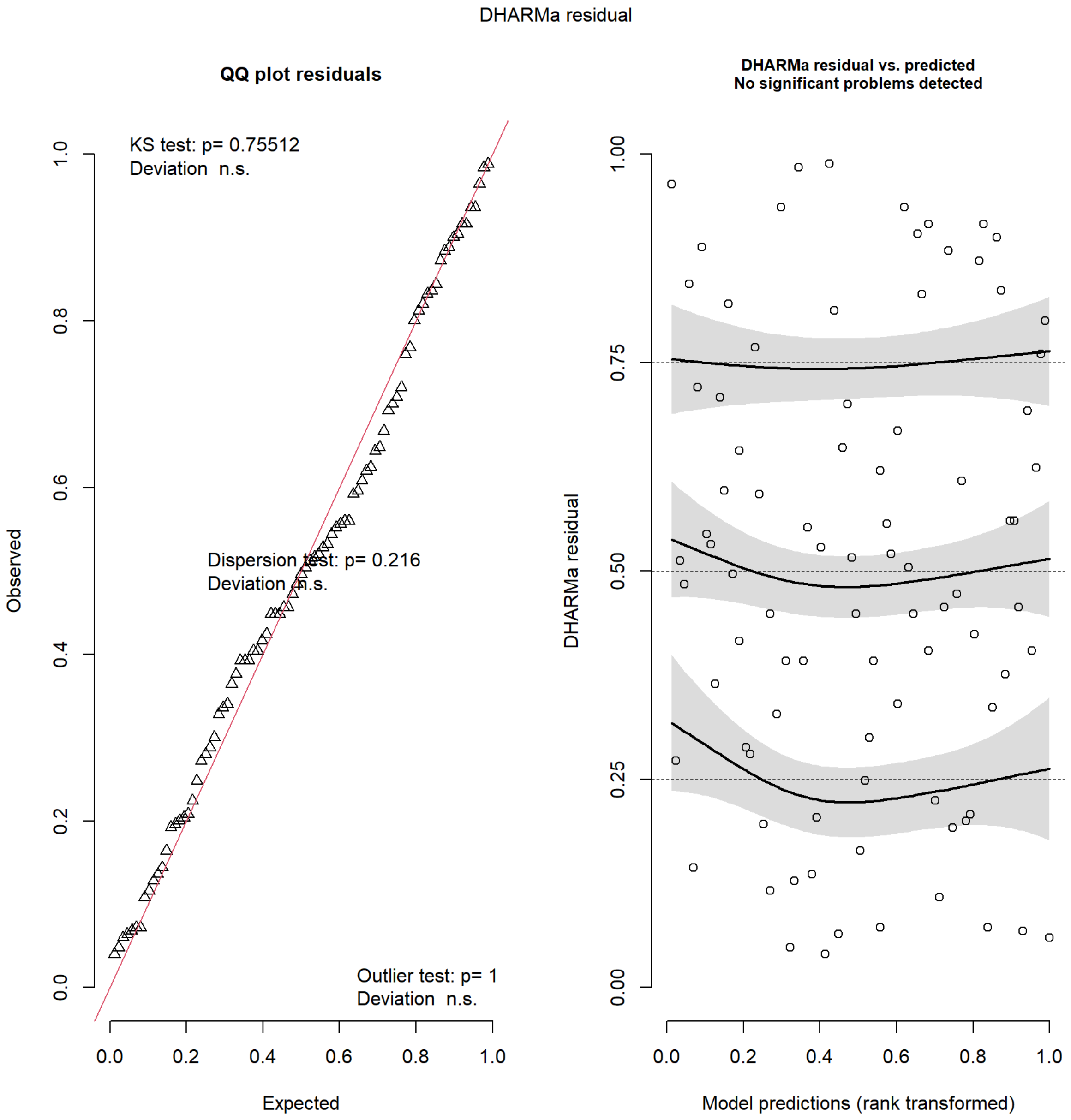

The model is ok.

Trim model

```
step_res<-step(mod1)

mod2<-get_model(step_res)

summary(mod2)
```

```
## Linear mixed model fit by REML. t-tests use Satterthwaite's method [
## lmerModLmerTest]
## Formula: shannon_diversity ~ chemo + enterocolitis + (1 | patient)
##    Data: dat3
##
## REML criterion at convergence: 172.1
##
## Scaled residuals:
##      Min       1Q   Median       3Q      Max
## -1.68232 -0.72369  0.06351  0.67067  2.34923
##
## Random effects:
##  Groups   Name        Variance Std.Dev.
##  patient  (Intercept) 0.1443   0.3799
##  Residual             0.3022   0.5497
## Number of obs: 87, groups:  patient, 29
##
## Fixed effects:
##                  Estimate Std. Error      df t value Pr(>|t|)
## (Intercept)        2.3237     0.1364 58.8368  17.031  < 2e-16 ***
## chemoYes          -0.6223     0.1250 57.0000  -4.978 6.28e-06 ***
## enterocolitisYes  -0.4782     0.2057 27.0000  -2.325   0.0278 *
## ---
## Signif. codes:  0 '***' 0.001 '**' 0.01 '*' 0.05 '.' 0.1 ' ' 1
##
## Correlation of Fixed Effects:
##             (Intr) chemYs
## chemoYes    -0.611
## entercltsYs -0.416  0.000
```

Only chemotherapy and enterocolitis are important.

```
simulationOutput <- simulateResiduals(mod2, plot = F)

plot(simulationOutput)
```

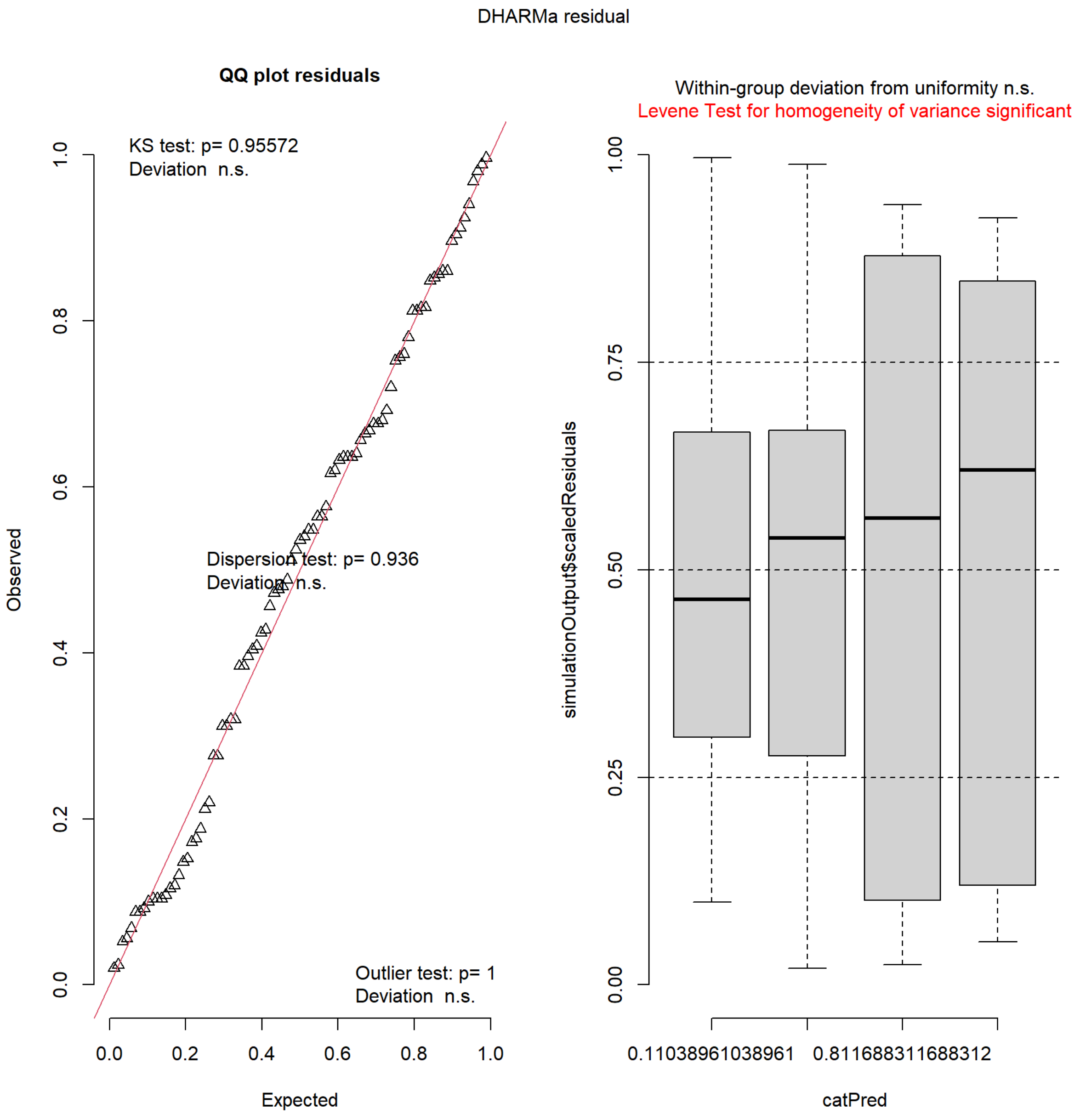

- The model is not ok.

log transform outcome

```
dat4<-dat3 %>% mutate(shannon_diversity = log(shannon_diversity))
```

```
mod3<-lmer(shannon_diversity ~ chemo+age+enterocolitis+

          stool_culture+ stool2+

          sepsis+ tazocin+ maxipime+ zithromax+meropenem+

          amikacin+vancomycin+colistin+tigecycline+

            (1|patient), data = dat4)

summary(mod3)
```

```
## Linear mixed model fit by REML. t-tests use Satterthwaite's method [
## lmerModLmerTest]
## Formula: shannon_diversity ~ chemo + age + enterocolitis + stool_culture +
##     stool2 + sepsis + tazocin + maxipime + zithromax + meropenem +
##     amikacin + vancomycin + colistin + tigecycline + (1 | patient)
##    Data: dat4
##
## REML criterion at convergence: 119.3
##
## Scaled residuals:
##      Min       1Q   Median       3Q      Max
## -2.88576 -0.49801  0.04136  0.55268  1.55451
##
## Random effects:
##  Groups   Name        Variance Std.Dev.
##  patient  (Intercept) 0.07486  0.2736
##  Residual             0.12945  0.3598
## Number of obs: 87, groups:  patient, 29
##
## Fixed effects:
##                                      Estimate Std. Error       df t value
## (Intercept)                           0.25370    0.37636 66.74998   0.674
## chemoYes                             -0.25941    0.11391 56.15947  -2.277
## age                                   0.02476    0.01477 23.54127   1.677
## enterocolitisYes                     -0.30050    0.15903 22.83357  -1.890
## stool_cultureE. coli                  0.31716    0.60106 56.28075   0.528
## stool_cultureEnterococcus             0.27654    0.62754 58.94467   0.441
## stool_cultureKlebsiella pneumoniae -0.08272    0.43936 58.39846  -0.188
## stool_cultureNegative                 0.15979    0.59051 58.59545   0.271
## stool2E. coli                        -0.03751    0.67557 57.39174  -0.056
## stool2Klebsiella pneumoniae           0.03325    0.51256 57.94787   0.065
## stool2Negative                       -0.06442    0.69182 58.42955  -0.093
## sepsisYes                             0.06363    0.13296 48.62459   0.479
## tazocinyes                            0.09331    0.15892 53.37112   0.587
## maxipimeyes                           0.13879    0.11048 63.12807   1.256
## zithromaxyes                         -0.08535    0.32842 64.11272  -0.260
## meropenemyes                         -0.01534    0.13460 54.48836  -0.114
## amikacinyes                           0.04732    0.11268 54.05536   0.420
## vancomycinyes                        -0.02560    0.11837 64.23780  -0.216
## colistinyes                           0.52552    0.37318 63.31414   1.408
## tigecyclineyes                       -0.35458    0.30762 66.56882  -1.153
##                                      Pr(>|t|)
## (Intercept)                            0.5026
## chemoYes                               0.0266 *
## age                                    0.1069
## enterocolitisYes                       0.0716 .
## stool_cultureE. coli                   0.5998
## stool_cultureEnterococcus              0.6611
## stool_cultureKlebsiella pneumoniae   0.8513
## stool_cultureNegative                  0.7876
## stool2E. coli                          0.9559
## stool2Klebsiella pneumoniae            0.9485
## stool2Negative                         0.9261
## sepsisYes                              0.6344
## tazocinyes                             0.5596
```

```
## maxipimeyes                       0.2137
## zithromaxyes                      0.7958
## meropenemyes                      0.9097
## amikacinyes                       0.6762
## vancomycinyes                     0.8295
## colistinyes                       0.1640
## tigecyclineyes                    0.2532
## ---
## Signif. codes:  0 '***' 0.001 '**' 0.01 '*' 0.05 '.' 0.1 ' ' 1
```

Model diagnostics

```
simulationOutput <- simulateResiduals(mod3, plot = F)

plot(simulationOutput)
```

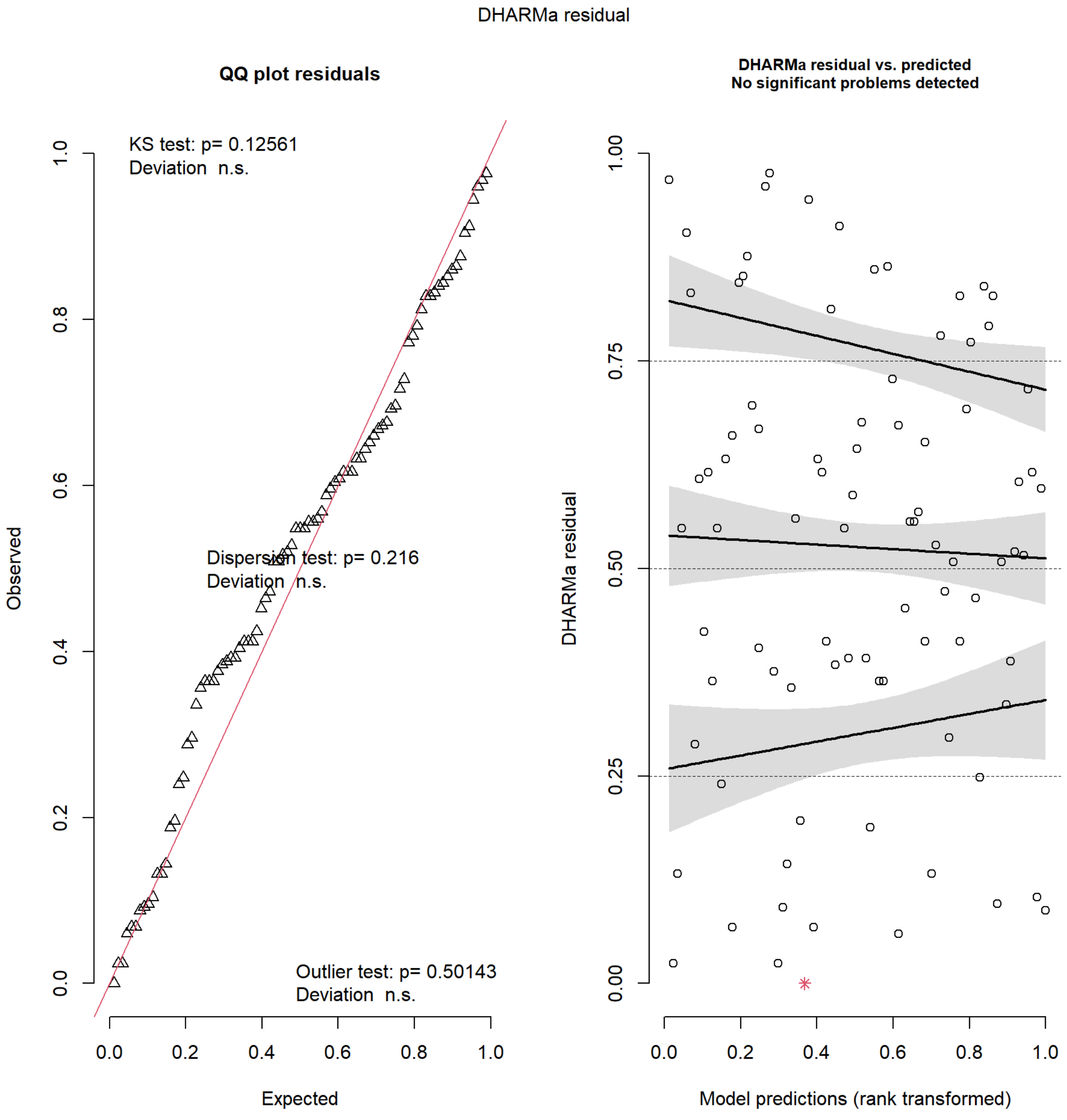

The model is ok.

Trim model

```
step_res<-step(mod3)

mod4<-get_model(step_res)

summary(mod4)
```

```
## Linear mixed model fit by REML. t-tests use Satterthwaite's method [
## lmerModLmerTest]
## Formula: shannon_diversity ~ chemo + age + enterocolitis + (1 | patient)
##    Data: dat4
##
## REML criterion at convergence: 103.5
##
## Scaled residuals:
##     Min      1Q  Median      3Q     Max
## -2.9417 -0.5458  0.1863  0.5811  1.7836
##
## Random effects:
##  Groups   Name        Variance Std.Dev.
##  patient  (Intercept) 0.05783  0.2405
##  Residual             0.12505  0.3536
## Number of obs: 87, groups:  patient, 29
##
## Fixed effects:
##                  Estimate Std. Error       df t value Pr(>|t|)
## (Intercept)       0.58567    0.13426 36.20860   4.362 0.000102 ***
## chemoYes         -0.34650    0.08043 57.00000  -4.308 6.57e-05 ***
## age               0.02544    0.01217 26.00000   2.090 0.046543 *
## enterocolitisYes -0.30135    0.13347 26.00000  -2.258 0.032576 *
## ---
## Signif. codes:  0 '***' 0.001 '**' 0.01 '*' 0.05 '.' 0.1 ' ' 1
##
## Correlation of Fixed Effects:
##             (Intr) chemYs age
## chemoYes    -0.399
## age         -0.760  0.000
## enterclstYs -0.408  0.000  0.189
```

Only chemotherapy, age and enterocolitis are important.

```
simulationOutput <- simulateResiduals(mod4, plot = F)

plot(simulationOutput)
```

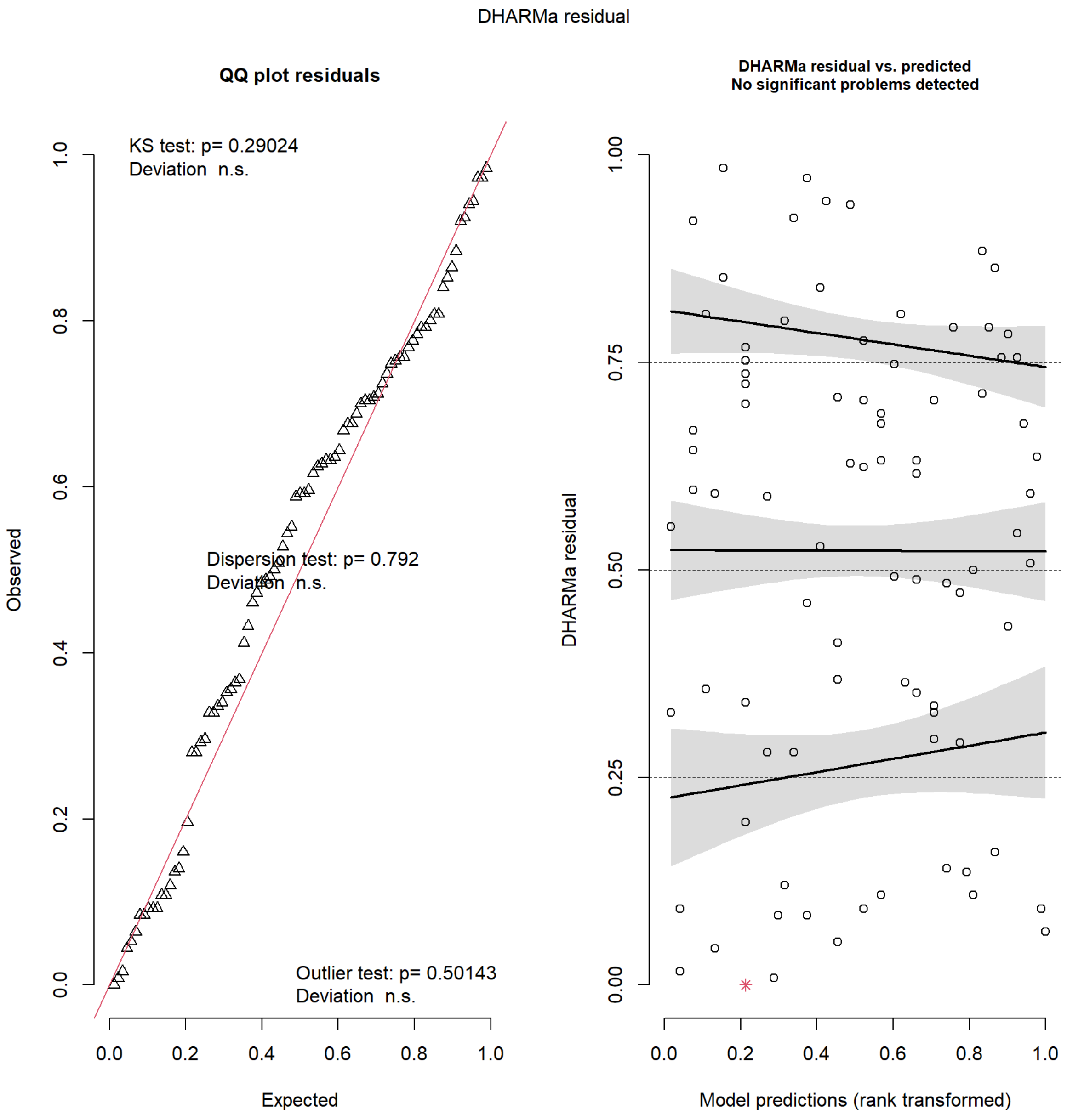

- The model is ok.
- Chemotherapy is associated with 29% decrease in shannon diversity than patients not receiving chemotherapy.
- Each 1 year increase in age is associated with 0.03 or 3% increase in shannon diversity.
- Enterocolitis is associated with 26% decrease in shannon diversity than patients without enterocolitis.

```
library(ggeffects)

df<-ggpredict(mod4, terms = ~ age + chemo+ enterocolitis)

df %>% mutate(chemo_enter = paste(group, facet, sep = "_")) %>%

  ggplot(aes(x = x, y = predicted, color = chemo_enter))+

  geom_point()+ geom_line(aes(group = chemo_enter))
```

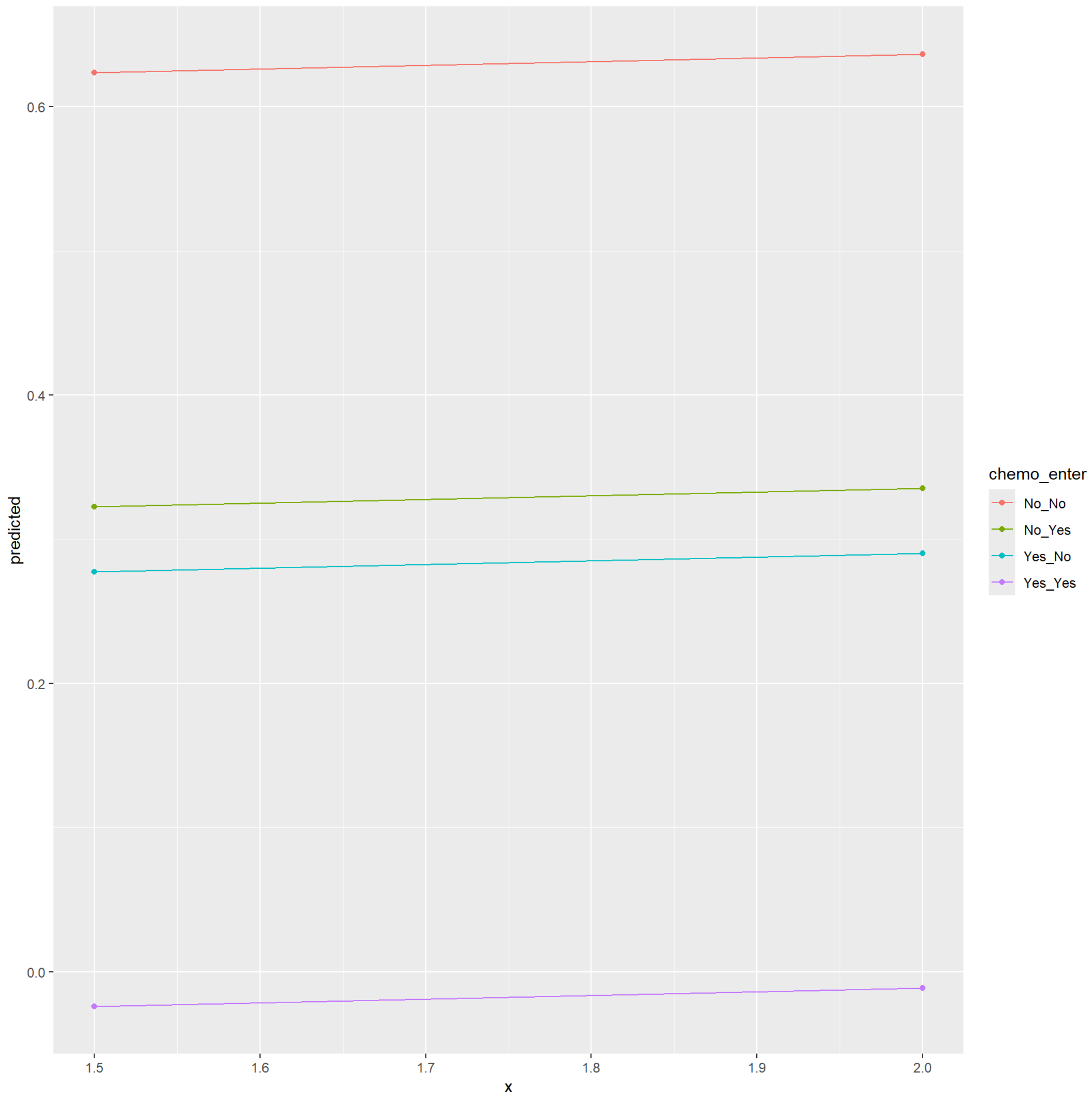

- The highest mean diversity was when chemotherapy and enterocolitis are absent.
- The lowest mean diversity when chemotherapy and enterocolitis are present.

Interaction with age

```
mod5<-lmer(shannon_diversity ~ chemo*age+enterocolitis*age+

            (1|patient), data = dat4)

summary(mod5)
```

```
## Linear mixed model fit by REML. t-tests use Satterthwaite's method [
## lmerModLmerTest]
## Formula: shannon_diversity ~ chemo * age + enterocolitis * age + (1 |
##     patient)
##    Data: dat4
##
## REML criterion at convergence: 113.4
##
## Scaled residuals:
##     Min      1Q  Median      3Q     Max
## -3.0447 -0.5348  0.1883  0.5706  1.6556
##
## Random effects:
##  Groups   Name        Variance Std.Dev.
##  patient  (Intercept) 0.0545   0.2334
##  Residual             0.1270   0.3564
## Number of obs: 87, groups:  patient, 29
##
## Fixed effects:
##                        Estimate Std. Error        df t value Pr(>|t|)
## (Intercept)            0.634325   0.169385 53.517916   3.745 0.000443 ***
## chemoYes              -0.301206   0.152518 56.000000  -1.975 0.053218 .
## age                    0.019903   0.017812 56.070214   1.117 0.268588
## enterocolitisYes      -0.546778   0.228739 24.999999  -2.390 0.024683 *
## chemoYes:age          -0.005799   0.016542 56.000000  -0.351 0.727212
## age:enterocolitisYes  0.035797   0.027283 24.999999   1.312 0.201417
## ---
## Signif. codes:  0 '***' 0.001 '**' 0.01 '*' 0.05 '.' 0.1 ' ' 1
##
## Correlation of Fixed Effects:
##             (Intr) chemYs age    entrcY chmYs:
## chemoYes    -0.600
## age         -0.858  0.524
## entercltsYs -0.474  0.000  0.402
## chemoYes:ag  0.509 -0.847 -0.619  0.000
## ag:ntrcltsY  0.355  0.000 -0.403 -0.818  0.000
```

Model diagnostics

```
simulationOutput <- simulateResiduals(mod5, plot = F)

plot(simulationOutput)
```

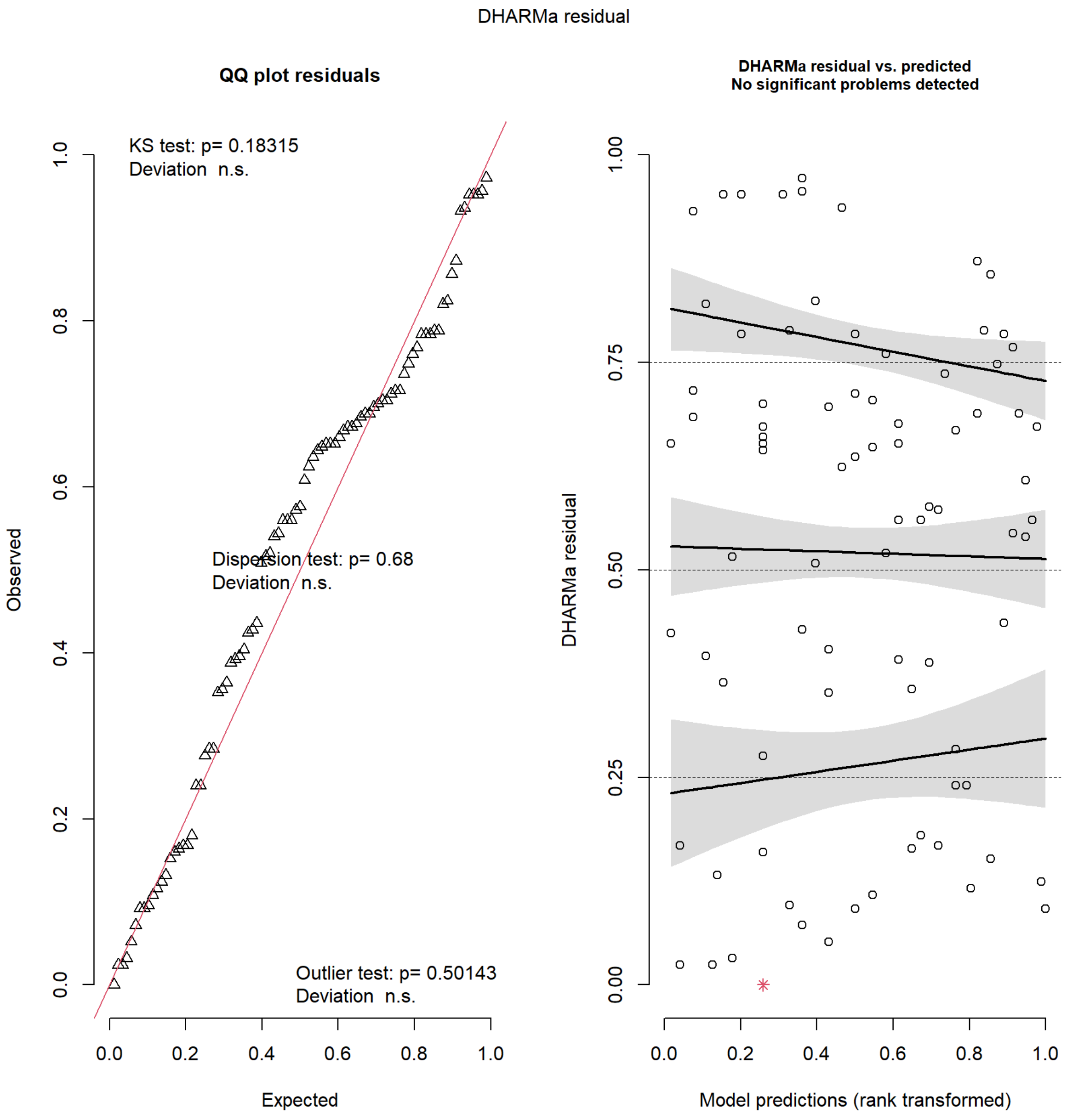

The model is ok.

Compare 2 models

```
anova(mod4, mod5)
```

```
## Data: dat4
## Models:
## mod4: shannon_diversity ~ chemo + age + enterocolitis + (1 | patient)
## mod5: shannon_diversity ~ chemo * age + enterocolitis * age + (1 | patient)
##      npar     AIC    BIC  logLik deviance  Chisq Df Pr(>Chisq)
## mod4    6  99.081 113.88 -43.541   87.081
## mod5    8 101.023 120.75 -42.511   85.023 2.0584  2     0.3573
```

Two models are equivalent.

Write the cleaned data

```
write_csv(dat4, file = "C:/Users/Dr Mohsen/Documents/R/mai_rami/dat_cleaned.csv")
```

mai_richness

- Loading packages and reading the data
- Richness
  - Multiple linear model
  - Linear mixed model
- Evenness
  - Multiple linear model
  - Linear mixed model
  - Trim the model
- f_to_b_ratio
  - Multiple linear model
  - Linear mixed model
- p_to_f_ratio
  - Multiple linear model
  - Linear mixed model

Loading packages and reading the data

```
library(tidyverse)

library(flextable)

library(DHARMa)

dat<-read_csv("C:/Users/Dr Mohsen/Documents/R/mai_rami/dat_cleaned.csv")

glimpse(dat)
```

```
## Rows: 87
## Columns: 30
## $ patient               <chr> "P01", "P01", "P01", "P02", "P02", "P02", "P03"…
## $ group                 <chr> "T1", "T2", "T3", "T1", "T2", "T3", "T1", "T2",…
## $ chemo                 <chr> "No", "Yes", "Yes", "No", "Yes", "Yes", "No", "…
## $ age                   <dbl> 10, 10, 10, 2, 2, 2, 13, 13, 13, 6, 6, 6, 2, 2,…
## $ stool_culture         <chr> "E. coli", "Acinetobacter baumannii", "Acinetob…
## $ stool2                <chr> "E. coli", "Acinetobacter baumannii", "Acinetob…
## $ blood                 <chr> "Staphylococcus", "Acinetobacter baumannii", "K…
## $ sepsis                <chr> "Yes", "Yes", "Yes", "No", "No", "No", "No", "N…
## $ f_to_b_ratio          <dbl> 5.941105e-01, 3.375436e-01, 8.519637e-02, 2.321…
## $ fvs_b                 <chr> "B>F", "B>F", "B>F", "F>B", "B>F", "B>F", "F>B"…
## $ shannon_diversity     <dbl> 0.86823041, 0.28824372, -0.57884361, 0.75217292…
## $ richness              <dbl> 43, 24, 12, 29, 28, 21, 57, 32, 12, 66, 40, 15,…
## $ evenness              <dbl> 0.6334921, 0.4197797, 0.2255804, 0.6300620, 0.5…
## $ p_to_f_ratio          <dbl> 6.424825e-01, 1.024196e+01, 1.551348e+02, 3.475…
## $ log2_p_to_f_ratio     <dbl> -0.6382709, 3.3564201, 7.2773781, -1.5247160, 2…
## $ fvs_p                 <chr> "F>P", "P>F", "P>F", "F>P", "P>F", "P>F", "F>P"…
## $ therapeutic_antibiotic <chr> "yes", "yes", "yes", "yes", "yes", "yes", "yes"…
## $ before_chemo          <chr> "yes", "no", "no", "no", "no", "no", "no", "no"…
## $ tazocin               <chr> "no", "no", "no", "no", "no", "no", "no", "no",…
## $ maxipime              <chr> "yes", "no", "no", "no", "yes", "no", "yes", "n…
## $ zithromax             <chr> "no", "no", "no", "no", "no", "no", "no", "no",…
## $ meropenem             <chr> "yes", "yes", "yes", "yes", "yes", "yes", "no",…
## $ amikacin              <chr> "yes", "no", "no", "yes", "yes", "yes", "no", "…
## $ vancomycin            <chr> "yes", "yes", "yes", "yes", "yes", "yes", "yes"…
## $ meropenem_high_dose   <chr> "yes", "yes", "yes", "no", "no", "no", "no", "n…
## $ colistin              <chr> "yes", "yes", "yes", "no", "no", "no", "no", "n…
## $ tigecycline           <chr> "yes", "yes", "yes", "no", "no", "no", "no", "n…
## $ meropenem_tazocin     <chr> "M", "M", "M", "M", "M", "M", "neither", "M", "…
## $ mero_tazo             <chr> "both", "both", "both", "no", "no", "no", "no",…
## $ enterocolitis         <chr> "Yes", "Yes", "Yes", "No", "No", "No", "No", "N…
```

The data is 87 rows and 30 columns.

Richness

Multiple linear model

```
mod<-lm(richness ~ chemo+age+enterocolitis+

          stool_culture+ stool2+

          sepsis+ tazocin+ maxipime+ zithromax+meropenem+

          amikacin+vancomycin+colistin+tigecycline, data = dat)

summary(mod)
```

```
##
## Call:
## lm(formula = richness ~ chemo + age + enterocolitis + stool_culture +
##     stool2 + sepsis + tazocin + maxipime + zithromax + meropenem +
##     amikacin + vancomycin + colistin + tigecycline, data = dat)
##
## Residuals:
##     Min      1Q  Median      3Q     Max
## -27.383 -10.933  -0.106   7.499  47.801
##
## Coefficients:
##                                    Estimate Std. Error t value Pr(>|t|)
## (Intercept)                         40.8781    15.3295   2.667 0.009595 **
## chemoYes                           -19.3032     5.0098  -3.853 0.000264 ***
## age                                  0.3676     0.4522   0.813 0.419152
## enterocolitisYes                   -12.9620     4.8217  -2.688 0.009053 **
## stool_cultureE. coli                -3.9896    25.7895  -0.155 0.877524
## stool_cultureEnterococcus            1.1687    26.5376   0.044 0.965003
## stool_cultureKlebsiella pneumoniae   4.1281    18.6168   0.222 0.825192
## stool_cultureNegative                0.2059    24.9266   0.008 0.993433
## stool2E. coli                        8.0552    28.8395   0.279 0.780867
## stool2Klebsiella pneumoniae         -8.7804    21.8334  -0.402 0.688850
## stool2Negative                       1.1751    29.3122   0.040 0.968141
## sepsisYes                           -3.9749     4.5818  -0.868 0.388738
## tazocinyes                           4.7976     5.6356   0.851 0.397627
## maxipimeyes                          3.4038     4.5551   0.747 0.457530
## zithromaxyes                        28.6187    13.4892   2.122 0.037574 *
## meropenemyes                         5.8826     5.9142   0.995 0.323483
## amikacinyes                         -7.4051     4.9463  -1.497 0.139069
## vancomycinyes                       -2.2131     4.3704  -0.506 0.614252
## colistinyes                         32.1020    13.8646   2.315 0.023662 *
## tigecyclineyes                     -21.7001    12.2975  -1.765 0.082190 .
## ---
## Signif. codes:  0 '***' 0.001 '**' 0.01 '*' 0.05 '.' 0.1 ' ' 1
##
## Residual standard error: 17.11 on 67 degrees of freedom
## Multiple R-squared:   0.48,  Adjusted R-squared:  0.3326
## F-statistic: 3.256 on 19 and 67 DF,  p-value: 0.0001869
```

Model diagnostics

```
simulationOutput <- simulateResiduals(mod, plot = F)

plot(simulationOutput)
```

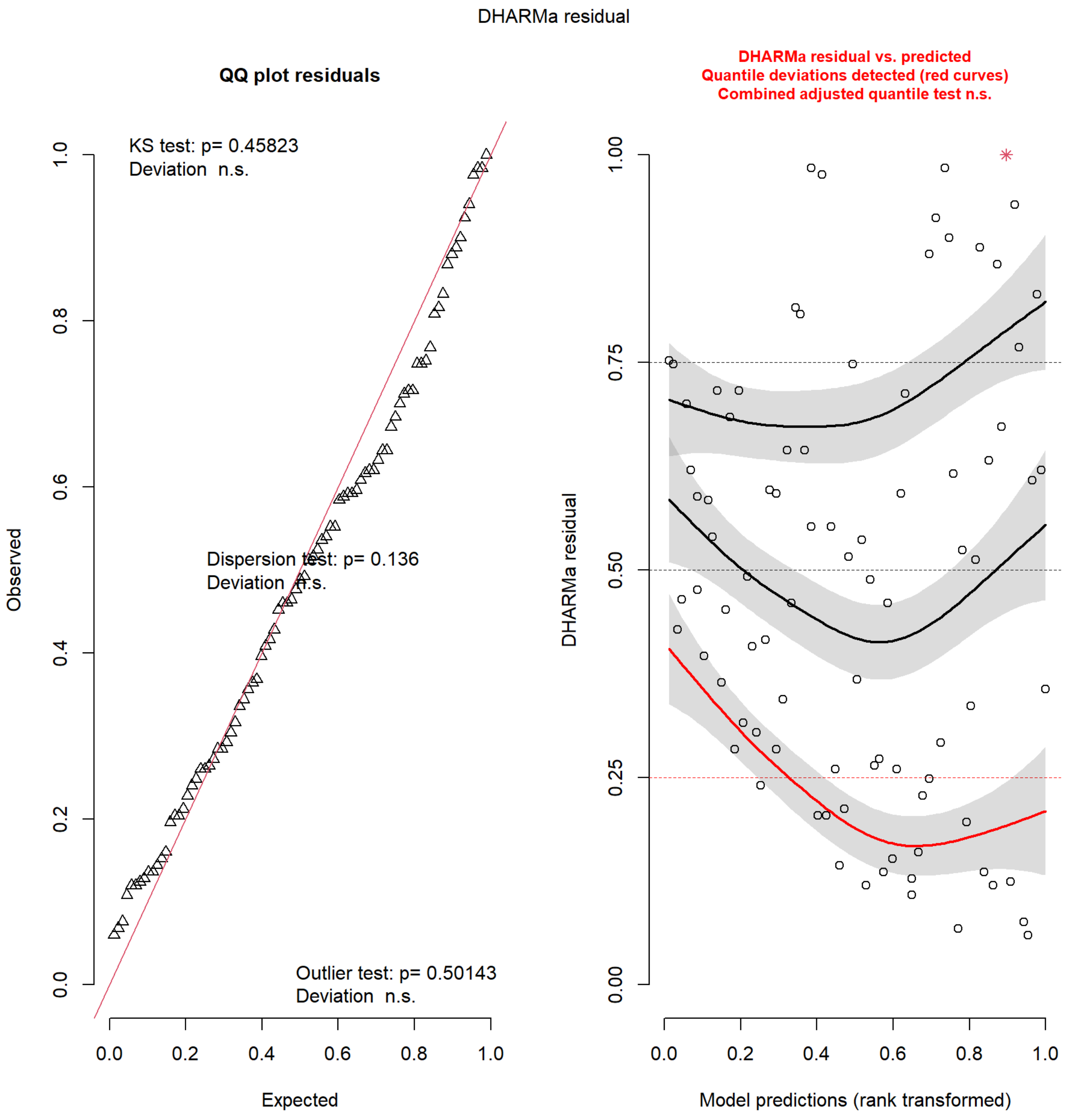

The model is not ok.

Linear mixed model

```
library(lme4)

library(lmerTest)

library(broom.mixed)

mod1<-lmer(richness ~ chemo+age+enterocolitis+

          stool_culture+ stool2+

          sepsis+ tazocin+ maxipime+ zithromax+meropenem+

          amikacin+vancomycin+colistin+tigecycline+

            (1|patient), data = dat)

summary(mod1)
```

```
## Linear mixed model fit by REML. t-tests use Satterthwaite's method [
## lmerModLmerTest]
## Formula: richness ~ chemo + age + enterocolitis + stool_culture + stool2 +
##     sepsis + tazocin + maxipime + zithromax + meropenem + amikacin +
##     vancomycin + colistin + tigecycline + (1 | patient)
##    Data: dat
##
## REML criterion at convergence: 605.4
##
## Scaled residuals:
##      Min       1Q   Median       3Q      Max
## -1.46885 -0.52828 -0.06433  0.42692  2.60929
##
## Random effects:
##  Groups   Name        Variance Std.Dev.
##  patient  (Intercept) 171.1    13.08
##  Residual             158.6    12.60
## Number of obs: 87, groups:  patient, 29
##
## Fixed effects:
##                                    Estimate Std. Error       df t value
## (Intercept)                         39.0533    14.0646  66.5450   2.777
## chemoYes                           -21.1286     4.1013  51.9389  -5.152
## age                                  0.3254     0.6291  22.7708   0.517
## enterocolitisYes                   -16.6506     6.7911  22.1804  -2.452
## stool_cultureE. coli                23.9040    21.5990  50.1389   1.107
## stool_cultureEnterococcus           31.5227    22.6866  53.0357   1.389
## stool_cultureKlebsiella pneumoniae  19.0744    15.8563  51.9634   1.203
## stool_cultureNegative               27.7988    21.3169  52.0470   1.304
## stool2E. coli                      -20.5816    24.3336  51.1945  -0.846
## stool2Klebsiella pneumoniae        -20.9778    18.4845  51.7325  -1.135
## stool2Negative                     -21.7728    24.9724  52.1360  -0.872
## sepsisYes                           -6.0365     5.2478  56.0509  -1.150
## tazocinyes                           6.9681     6.2144  59.9493   1.121
## maxipimeyes                          6.8447     4.0368  57.7183   1.696
## zithromaxyes                        21.0435    12.0480  59.7842   1.747
## meropenemyes                         7.0950     4.8262  49.7980   1.470
## amikacinyes                         -8.0691     4.0366  49.3313  -1.999
## vancomycinyes                       -1.6483     4.4919  66.9759  -0.367
## colistinyes                         56.9834    14.2436  66.6150   4.001
## tigecyclineyes                     -32.8660    11.4113  63.5049  -2.880
##                                    Pr(>|t|)
## (Intercept)                        0.007125 **
## chemoYes                           4.05e-06 ***
## age                                0.609991
## enterocolitisYes                   0.022545 *
## stool_cultureE. coli               0.273699
## stool_cultureEnterococcus          0.170491
## stool_cultureKlebsiella pneumoniae 0.234447
## stool_cultureNegative              0.197947
## stool2E. coli                      0.401596
## stool2Klebsiella pneumoniae        0.261655
## stool2Negative                     0.387276
## sepsisYes                          0.254907
## tazocinyes                         0.266641
```

```
## maxipimeyes                       0.095354 .
## zithromaxyes                      0.085836 .
## meropenemyes                      0.147826
## amikacinyes                       0.051134 .
## vancomycinyes                     0.714807
## colistinyes                       0.000161 ***
## tigecyclineyes                    0.005413 **
## ---
## Signif. codes:  0 '***' 0.001 '**' 0.01 '*' 0.05 '.' 0.1 ' ' 1
```

Model diagnostics

```
simulationOutput <- simulateResiduals(mod1, plot = F)

plot(simulationOutput)
```

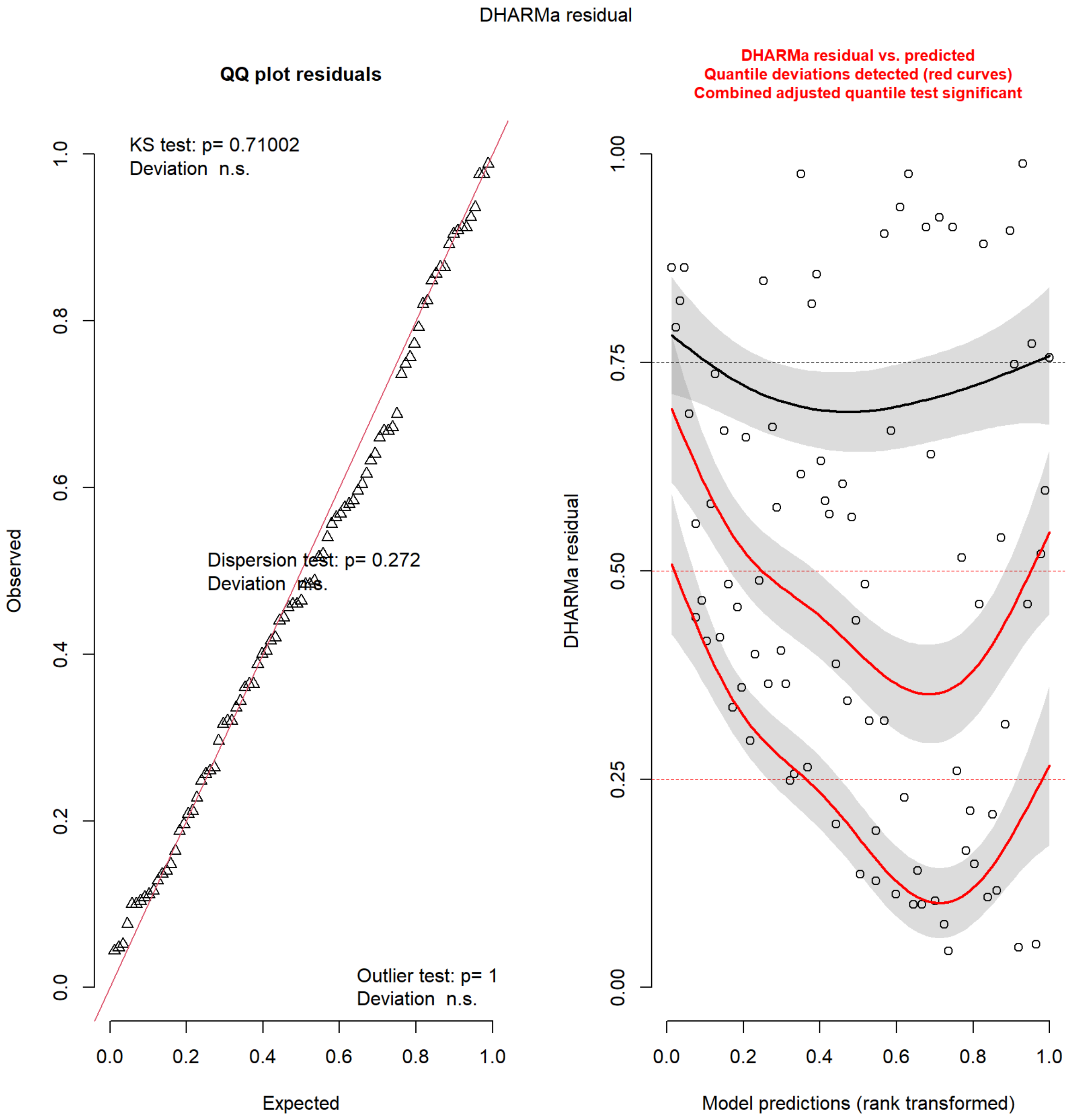

The model is not ok.

Log transformation

```
dat2<-dat %>% mutate(richness = log(richness))
```

```
mod2<-lmer(richness ~ chemo+age+enterocolitis+

          stool_culture+ stool2+

          sepsis+ tazocin+ maxipime+ zithromax+meropenem+

          amikacin+vancomycin+colistin+tigecycline+

            (1|patient), data = dat2)

summary(mod2)
```

```
## Linear mixed model fit by REML. t-tests use Satterthwaite's method [
## lmerModLmerTest]
## Formula: richness ~ chemo + age + enterocolitis + stool_culture + stool2 +
##     sepsis + tazocin + maxipime + zithromax + meropenem + amikacin +
##     vancomycin + colistin + tigecycline + (1 | patient)
##    Data: dat2
##
## REML criterion at convergence: 141.7
##
## Scaled residuals:
##      Min       1Q   Median       3Q      Max
## -1.56548 -0.56854  0.09548  0.48231  1.64662
##
## Random effects:
##  Groups   Name        Variance Std.Dev.
##  patient  (Intercept) 0.1625   0.4031
##  Residual             0.1588   0.3985
## Number of obs: 87, groups:  patient, 29
##
## Fixed effects:
##                                    Estimate Std. Error        df t value
## (Intercept)                        3.481277   0.442331 66.604523   7.870
## chemoYes                          -0.597587   0.129459 53.859125  -4.616
## age                                0.006661   0.019549 24.960737   0.341
## enterocolitisYes                  -0.522067   0.210998 24.339355  -2.474
## stool_cultureE. coli               0.532886   0.681996 52.300225   0.781
## stool_cultureEnterococcus          0.652285   0.715950 54.969999   0.911
## stool_cultureKlebsiella pneumoniae  0.528424   0.500495 54.028846   1.056
## stool_cultureNegative              0.625022   0.672847 54.109135   0.929
## stool2E. coli                     -0.439762   0.768190 53.294643  -0.572
## stool2Klebsiella pneumoniae       -0.679296   0.583480 53.800369  -1.164
## stool2Negative                    -0.515159   0.788216 54.174378  -0.654
## sepsisYes                         -0.054148   0.164332 56.623239  -0.330
## tazocinyes                         0.318560   0.194757 60.229605   1.636
## maxipimeyes                        0.276602   0.127278 59.240046   2.173
## zithromaxyes                       0.225838   0.379705 61.005016   0.595
## meropenemyes                       0.127912   0.152401 51.882768   0.839
## amikacinyes                       -0.192935   0.127477 51.439398  -1.513
## vancomycinyes                     -0.013904   0.141185 66.999898  -0.098
## colistinyes                        1.725192   0.447358 66.519921   3.856
## tigecyclineyes                    -1.124436   0.359306 64.272815  -3.129
##                                   Pr(>|t|)
## (Intercept)                       4.27e-11 ***
## chemoYes                          2.46e-05 ***
## age                               0.736170
## enterocolitisYes                  0.020699 *
## stool_cultureE. coli              0.438112
## stool_cultureEnterococcus         0.366234
## stool_cultureKlebsiella pneumoniae 0.295757
## stool_cultureNegative             0.357055
## stool2E. coli                     0.569414
## stool2Klebsiella pneumoniae       0.249474
## stool2Negative                    0.516150
## sepsisYes                         0.742990
## tazocinyes                        0.107122
```

```
## maxipimeyes                      0.033775 *
## zithromaxyes                     0.554195
## meropenemyes                     0.405144
## amikacinyes                      0.136274
## vancomycinyes                    0.921845
## colistinyes                      0.000262 ***
## tigecyclineyes                   0.002633 **
## ---
## Signif. codes:  0 '***' 0.001 '**' 0.01 '*' 0.05 '.' 0.1 ' ' 1
```

Model diagnostics

```
simulationOutput <- simulateResiduals(mod2, plot = F)

plot(simulationOutput)
```

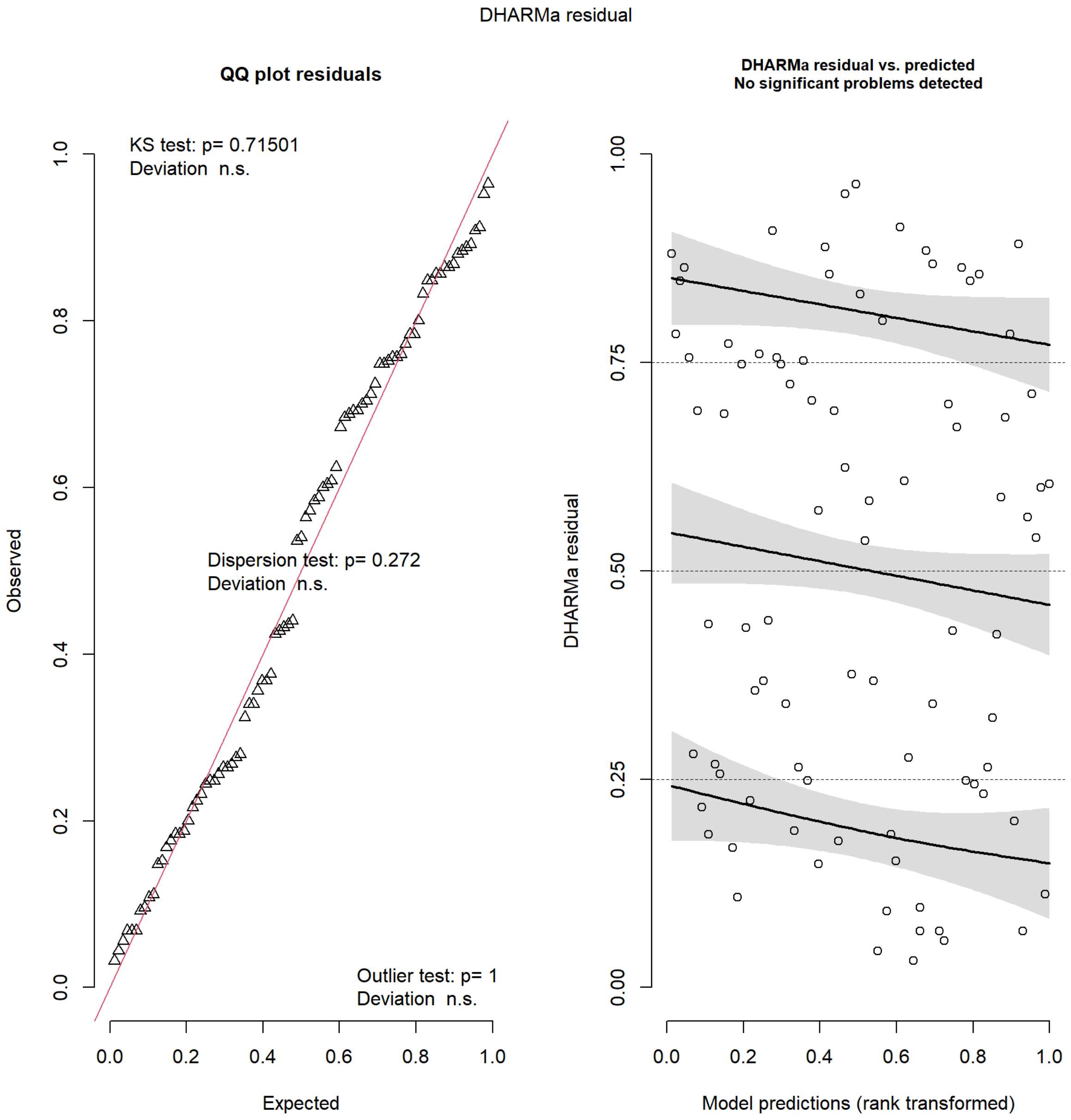

The model is ok.

Trim model

```
step_res<-step(mod2)

mod3<-get_model(step_res)

summary(mod3)
```

```
## Linear mixed model fit by REML. t-tests use Satterthwaite's method [
## lmerModLmerTest]
## Formula:
## richness ~ chemo + enterocolitis + maxipime + colistin + tigecycline +
##     (1 | patient)
##    Data: dat2
##
## REML criterion at convergence: 129.4
##
## Scaled residuals:
##     Min      1Q  Median      3Q     Max
## -1.8846 -0.6706  0.1025  0.4915  1.8434
##
## Random effects:
##  Groups   Name        Variance Std.Dev.
##  patient  (Intercept) 0.1623   0.4029
##  Residual             0.1502   0.3875
## Number of obs: 87, groups:  patient, 29
##
## Fixed effects:
##                  Estimate Std. Error       df t value Pr(>|t|)
## (Intercept)       3.70250    0.12870 53.87322  28.768  < 2e-16 ***
## chemoYes         -0.69182    0.09008 55.50459  -7.680 2.75e-10 ***
## enterocolitisYes -0.49721    0.19555 26.25007  -2.543  0.01723 *
## maxipimeyes       0.23913    0.10988 74.58199   2.176  0.03271 *
## colistinyes       1.43770    0.39637 80.76393   3.627  0.00050 ***
## tigecyclineyes   -0.93745    0.30731 76.98206  -3.050  0.00313 **
## ---
## Signif. codes:  0 '***' 0.001 '**' 0.01 '*' 0.05 '.' 0.1 ' ' 1
##
## Correlation of Fixed Effects:
##             (Intr) chemYs entrcY mxpmys clstny
## chemoYes    -0.506
## entercltsYs -0.437  0.036
## maxipimeyes -0.411  0.143  0.064
## colistinyes  0.037 -0.080 -0.163  0.118
## tigecyclnys -0.006 -0.037  0.053 -0.246 -0.750
```

Only chemotherapy, enterocolitis, maxipime, colistin, tigecycline are important.

```
simulationOutput <- simulateResiduals(mod3, plot = F)

plot(simulationOutput)
```

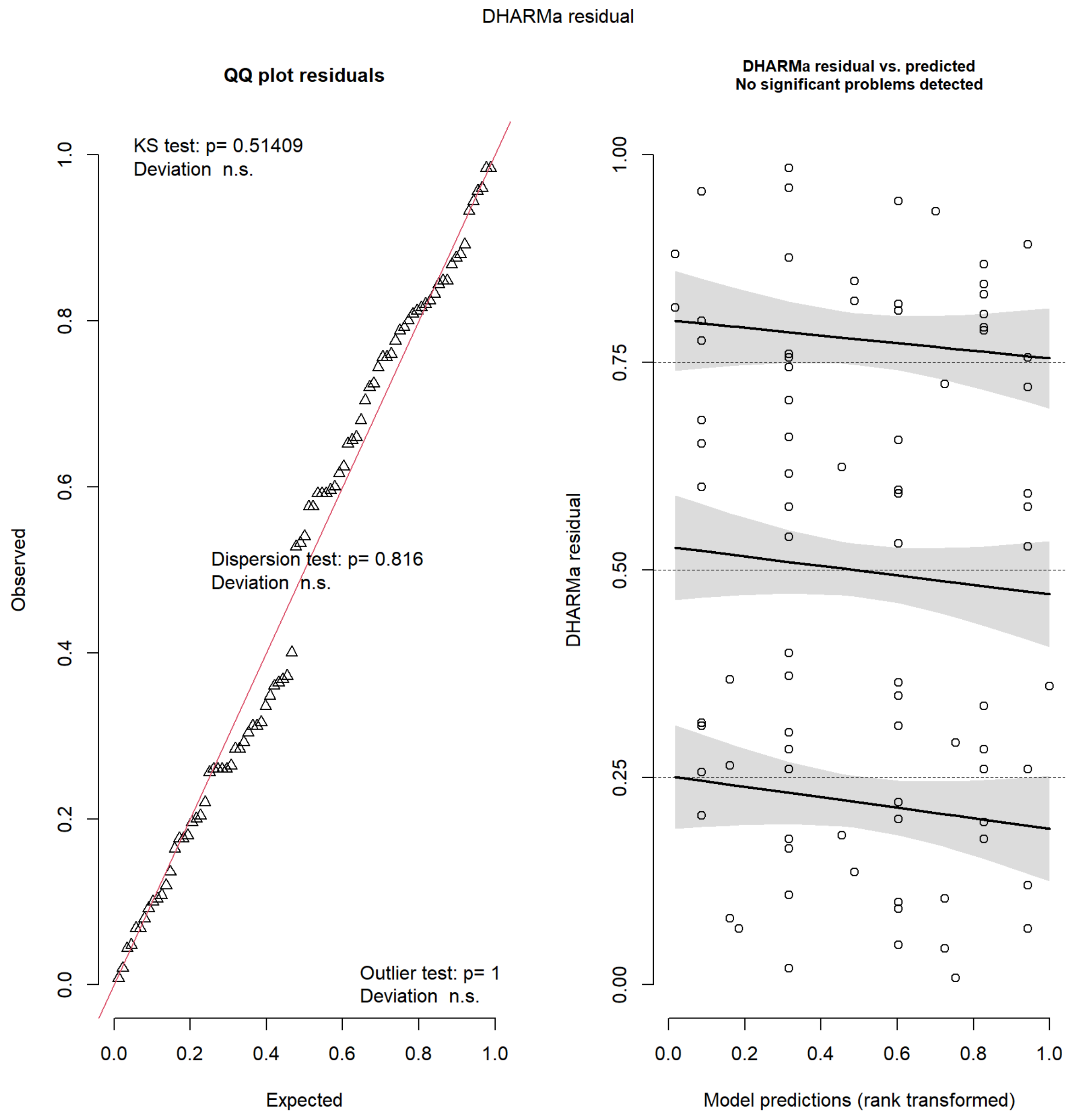

The model is ok.

- Chemotherapy is associated with 1-exp(-0.69182) 50% decrease in richness than patients not receiving chemotherapy.
- Enterocolitis is associated with 1-exp(-0.49721) 40% decrease in richness than patients without enterocolitis.
- Maxipime is associated with exp(0.23913)-1 = 27% increase in richness than patients not using Maxipime.
- Colistin is associated with exp(1.43770)-1 = 321% increase in richness than patients not using Colistin.
- Tigecycline is associated with 1-exp(-0.93745)= 61% decrease in richness than patients not using Tigecycline.

```r
library(ggeffects)

df<-ggpredict(mod3, terms = ~ chemo + enterocolitis + maxipime + colistin +

                tigecycline)

df %>% dplyr::rename(chemo = x, entero = group, max = facet, col = panel,

              tig = grid) %>%

  mutate(chemo_entero_max_col_tig = paste( chemo,entero,max,col,tig,

                                          sep = "_")) %>%

  arrange(predicted) %>%

  mutate(chemo_entero_max_col_tig = fct_inorder(chemo_entero_max_col_tig)) %>%

  ggplot(aes(x = predicted, y = chemo_entero_max_col_tig))+

  geom_point()+ geom_line(aes(group = 1))
```

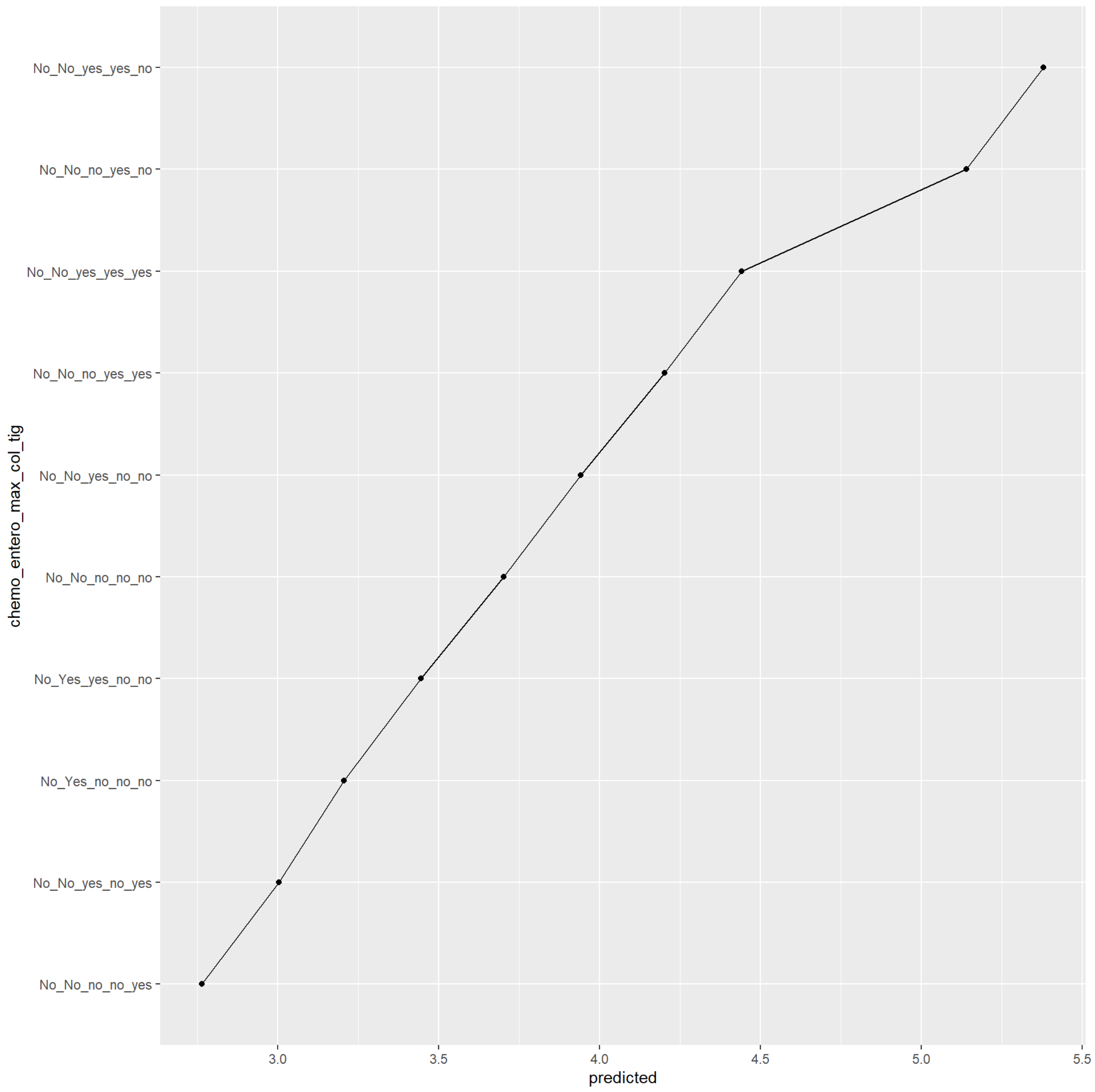

- The highest mean predicted richness was when chemotherapy and enterocolitis are absent and maxipime and colistin are administered.
- The lowest mean richness when only tigecycline was administered in the absence of chemotherapy and enterocolitis.

Evenness

Multiple linear model

```
mod4<-lm(evenness ~ chemo+age+enterocolitis+

          stool_culture+ stool2+

          sepsis+ tazocin+ maxipime+ zithromax+meropenem+

          amikacin+vancomycin+colistin+tigecycline, data = dat)

summary(mod4)
```

```
##
## Call:
## lm(formula = evenness ~ chemo + age + enterocolitis + stool_culture +
##     stool2 + sepsis + tazocin + maxipime + zithromax + meropenem +
##     amikacin + vancomycin + colistin + tigecycline, data = dat)
##
## Residuals:
##      Min       1Q   Median       3Q      Max
## -0.40115 -0.08279  0.00533  0.08276  0.30143
##
## Coefficients:
##                                     Estimate Std. Error t value Pr(>|t|)
## (Intercept)                         0.375773   0.137765   2.728  0.00814 **
## chemoYes                           -0.035464   0.045022  -0.788  0.43366
## age                                 0.009019   0.004064   2.219  0.02986 *
## enterocolitisYes                   -0.039987   0.043332  -0.923  0.35943
## stool_cultureE. coli                0.013972   0.231768   0.060  0.95211
## stool_cultureEnterococcus          -0.029940   0.238491  -0.126  0.90047
## stool_cultureKlebsiella pneumoniae -0.109413   0.167307  -0.654  0.51537
## stool_cultureNegative              -0.056527   0.224013  -0.252  0.80155
## stool2E. coli                       0.139412   0.259178   0.538  0.59243
## stool2Klebsiella pneumoniae         0.149118   0.196215   0.760  0.44994
## stool2Negative                      0.094314   0.263426   0.358  0.72145
## sepsisYes                           0.045928   0.041176   1.115  0.26866
## tazocinyes                         -0.015214   0.050646  -0.300  0.76480
## maxipimeyes                         0.012644   0.040936   0.309  0.75838
## zithromaxyes                        0.021596   0.121227   0.178  0.85914
## meropenemyes                       -0.001353   0.053150  -0.025  0.97977
## amikacinyes                         0.034472   0.044452   0.775  0.44079
## vancomycinyes                      -0.005417   0.039276  -0.138  0.89072
## colistinyes                        -0.065215   0.124600  -0.523  0.60243
## tigecyclineyes                      0.027750   0.110517   0.251  0.80251
## ---
## Signif. codes:  0 '***' 0.001 '**' 0.01 '*' 0.05 '.' 0.1 ' ' 1
##
## Residual standard error: 0.1538 on 67 degrees of freedom
## Multiple R-squared:  0.2811, Adjusted R-squared:  0.07719
## F-statistic: 1.379 on 19 and 67 DF,  p-value: 0.1682
```

Model diagnostics

```
simulationOutput <- simulateResiduals(mod4, plot = F)

plot(simulationOutput)
```

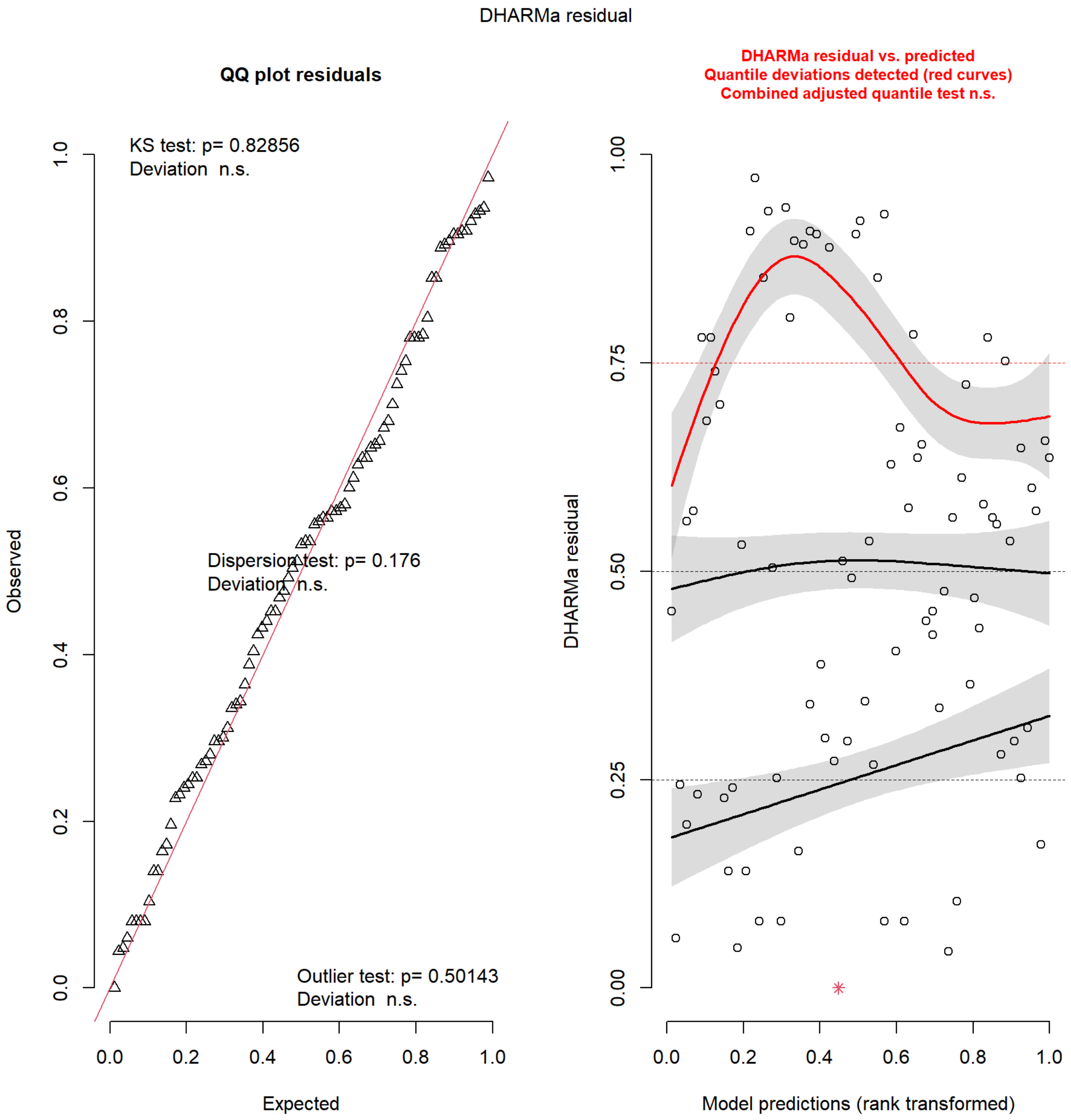

The model is not ok.

Linear mixed model

```
mod5<-lmer(evenness ~ chemo+age+enterocolitis+

          stool_culture+ stool2+

          sepsis+ tazocin+ maxipime+ zithromax+meropenem+

          amikacin+vancomycin+colistin+tigecycline+

            (1|patient), data = dat)

summary(mod5)
```

```
## Linear mixed model fit by REML. t-tests use Satterthwaite's method [
## lmerModLmerTest]
## Formula: evenness ~ chemo + age + enterocolitis + stool_culture + stool2 +
##     sepsis + tazocin + maxipime + zithromax + meropenem + amikacin +
##     vancomycin + colistin + tigecycline + (1 | patient)
##    Data: dat
##
## REML criterion at convergence: -15.5
##
## Scaled residuals:
##     Min      1Q  Median      3Q     Max
## -2.4559 -0.5246  0.0388  0.5055  1.7784
##
## Random effects:
##  Groups   Name        Variance Std.Dev.
##  patient  (Intercept) 0.002088 0.04569
##  Residual             0.021877 0.14791
## Number of obs: 87, groups:  patient, 29
##
## Fixed effects:
##                                      Estimate Std. Error         df t value
## (Intercept)                         0.3868860  0.1379598 66.8204731   2.804
## chemoYes                           -0.0381162  0.0442031 60.1850888  -0.862
## age                                 0.0090306  0.0043588 21.4403115   2.072
## enterocolitisYes                   -0.0451542  0.0466214 20.3283408  -0.969
## stool_cultureE. coli                0.0443690  0.2303037 65.6748885   0.193
## stool_cultureEnterococcus           0.0011291  0.2378712 66.6972720   0.005
## stool_cultureKlebsiella pneumoniae -0.0964236  0.1667895 66.6155149  -0.578
## stool_cultureNegative              -0.0276613  0.2236872 66.8412750  -0.124
## stool2E. coli                       0.1010021  0.2577972 66.0643549   0.392
## stool2Klebsiella pneumoniae         0.1277735  0.1952235 66.1879063   0.654
## stool2Negative                      0.0667707  0.2626318 66.6202540   0.254
## sepsisYes                           0.0395878  0.0431551 35.1295730   0.917
## tazocinyes                         -0.0153404  0.0526456 41.1538002  -0.291
## maxipimeyes                         0.0143377  0.0410759 66.7751546   0.349
## zithromaxyes                       -0.0003723  0.1216764 66.6887350  -0.003
## meropenemyes                       -0.0035545  0.0523258 61.3471848  -0.068
## amikacinyes                         0.0341526  0.0438146 61.8600052   0.779
## vancomycinyes                      -0.0069148  0.0405987 47.1492585  -0.170
## colistinyes                        -0.0421784  0.1283074 49.9264363  -0.329
## tigecyclineyes                      0.0201751  0.1116067 64.4366775   0.181
##                                    Pr(>|t|)
## (Intercept)                          0.0066 **
## chemoYes                             0.3919
## age                                  0.0505 .
## enterocolitisYes                     0.3442
## stool_cultureE. coli                 0.8478
## stool_cultureEnterococcus            0.9962
## stool_cultureKlebsiella pneumoniae   0.5651
## stool_cultureNegative                0.9020
## stool2E. coli                        0.6965
## stool2Klebsiella pneumoniae          0.5151
## stool2Negative                       0.8001
## sepsisYes                            0.3652
## tazocinyes                           0.7722
```

```
## maxipimeyes                      0.7281
## zithromaxyes                     0.9976
## meropenemyes                     0.9461
## amikacinyes                      0.4387
## vancomycinyes                    0.8655
## colistinyes                      0.7437
## tigecyclineyes                   0.8571
## ---
## Signif. codes:  0 '***' 0.001 '**' 0.01 '*' 0.05 '.' 0.1 ' ' 1
```

Model diagnostics

```
simulationOutput <- simulateResiduals(mod5, plot = F)

plot(simulationOutput)
```

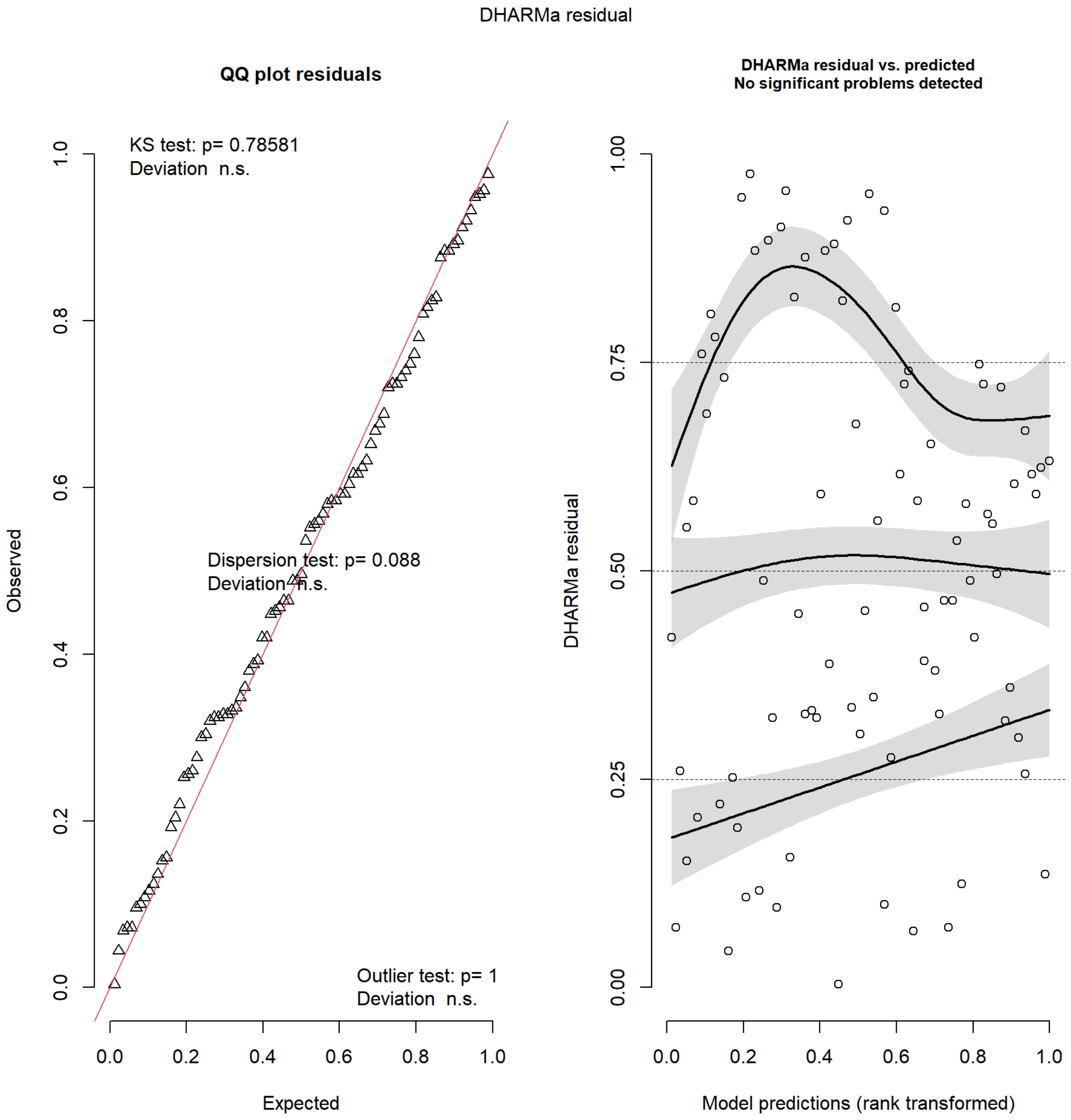

The model is ok.

Trim the model

```
step_res<-step(mod5)

mod6<-get_model(step_res)

summary(mod6)
```

```
##
## Call:
## lm(formula = evenness ~ age + stool_culture, data = dat)
##
## Residuals:
##      Min       1Q   Median       3Q      Max
## -0.41093 -0.08787  0.01512  0.08871  0.29126
##
## Coefficients:
##                                    Estimate Std. Error t value Pr(>|t|)
## (Intercept)                        0.371751   0.080102   4.641 1.32e-05 ***
## age                                0.009159   0.003493   2.622   0.0104 *
## stool_cultureE. coli               0.159146   0.078015   2.040   0.0446 *
## stool_cultureEnterococcus          0.070462   0.080537   0.875   0.3842
## stool_cultureKlebsiella pneumoniae 0.032840   0.085187   0.386   0.7009
## stool_cultureNegative              0.079125   0.084391   0.938   0.3512
## ---
## Signif. codes:  0 '***' 0.001 '**' 0.01 '*' 0.05 '.' 0.1 ' ' 1
##
## Residual standard error: 0.1474 on 81 degrees of freedom
## Multiple R-squared:  0.202,  Adjusted R-squared:  0.1528
## F-statistic: 4.101 on 5 and 81 DF,  p-value: 0.002272
```

- Only age and E.coli in stool culture are important.

```
simulationOutput <- simulateResiduals(mod6, plot = F)

plot(simulationOutput)
```

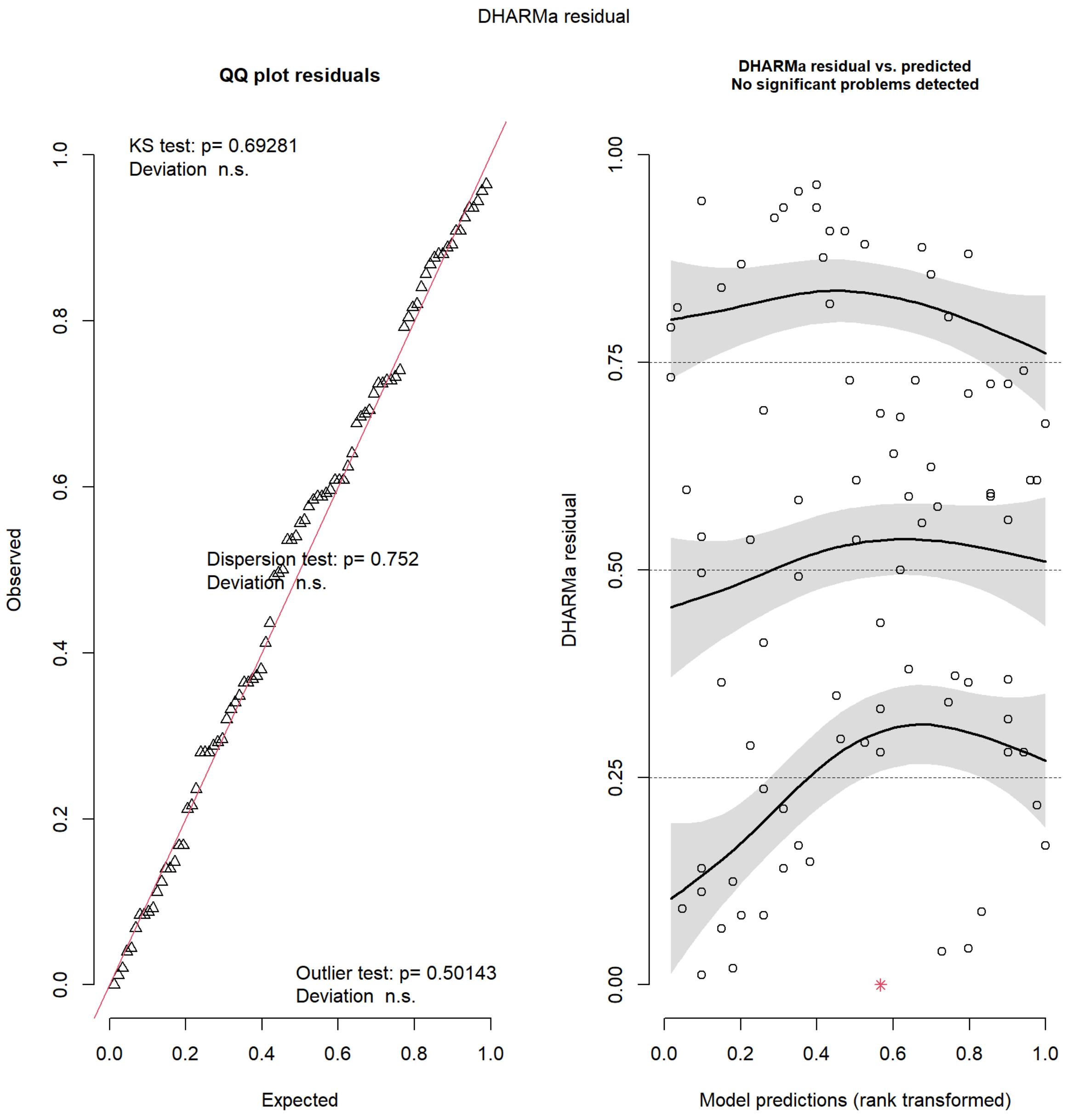

- The model is ok.
- Each 1 year increase in age is associated with 0.009 increase in evenness when all other factors are constant.
- Patients having E.coli in their stool culture have 0.16 increase in evenness than patients having Acinetobacter baumannii (reference group).

```
library(ggeffects)

df<-ggpredict(mod6, terms = ~ age + stool_culture)

df %>% dplyr::rename(age = x, stool_culture = group) %>%


  ggplot(aes(x = age, y = predicted, color = stool_culture))+

  geom_point()+ geom_line(aes(group = stool_culture))
```

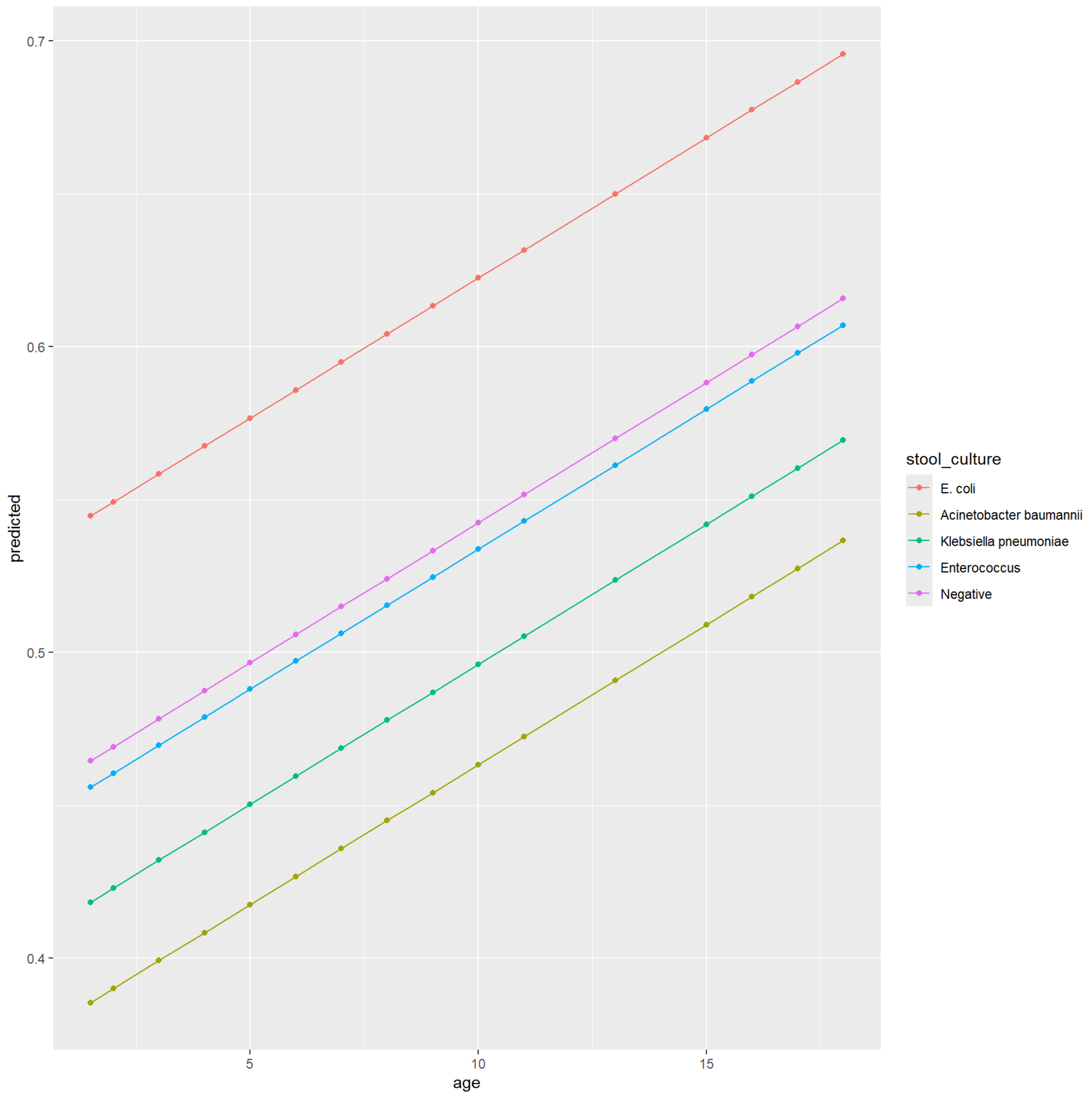

- The highest predicted evenness is when E.coli is present in stool culture.
- The lowest predicted evenness is when A.baumannii is present in stool culture.

---

f_to_b_ratio

Multiple linear model

```
mod7<-lm(f_to_b_ratio ~ chemo+age+enterocolitis+

          stool_culture+ stool2+

          sepsis+ tazocin+ maxipime+ zithromax+meropenem+

          amikacin+vancomycin+colistin+tigecycline, data = dat)
```

Model diagnostics

```
simulationOutput <- simulateResiduals(mod7, plot = F)

plot(simulationOutput)
```

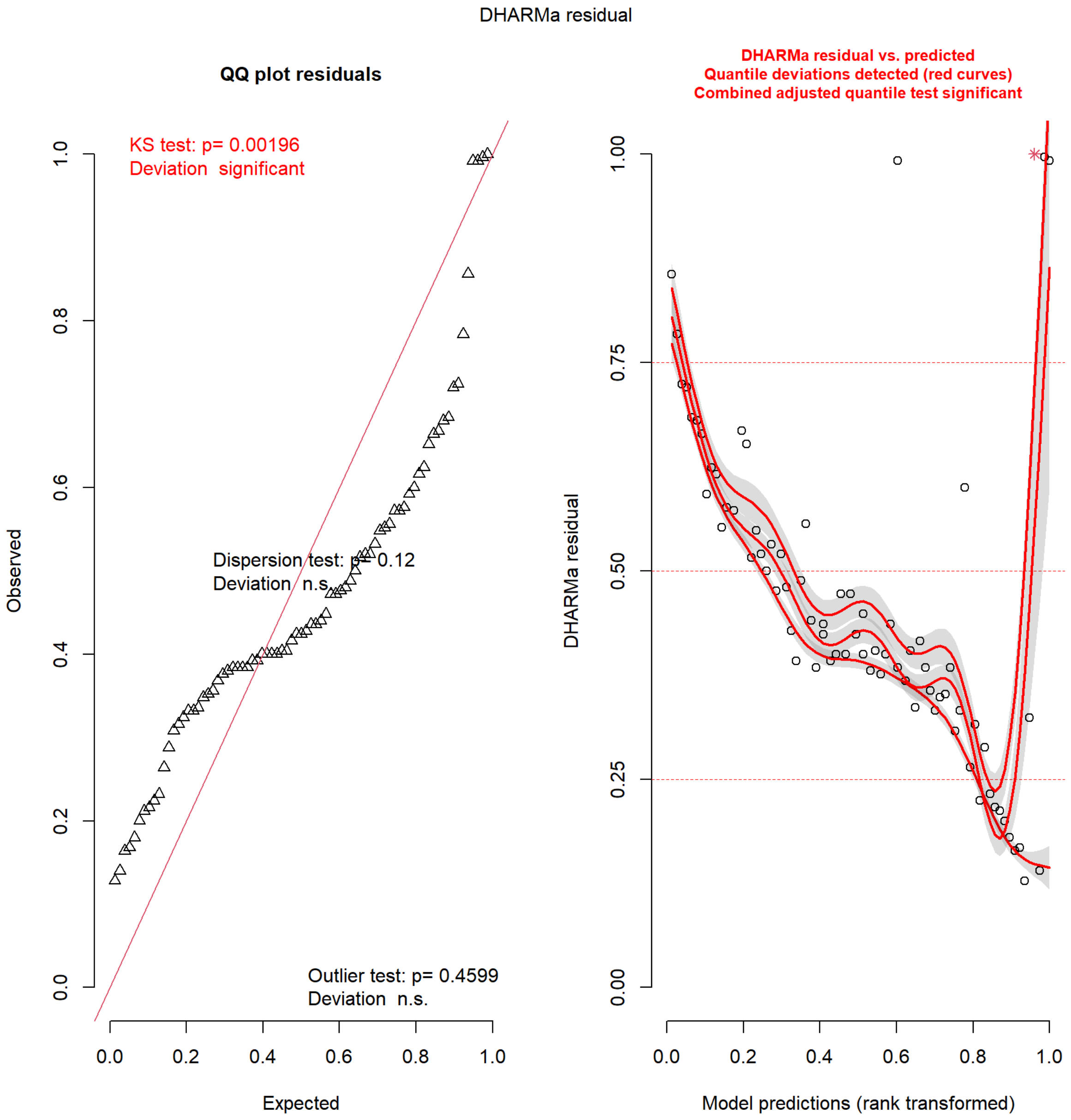

The model is not ok.

Linear mixed model

```
mod8<-lmer(f_to_b_ratio ~ chemo+age+enterocolitis+

          stool_culture+ stool2+

          sepsis+ tazocin+ maxipime+ zithromax+meropenem+

          amikacin+vancomycin+colistin+tigecycline+

            (1|patient), data = dat)
```

Model diagnostics

```
simulationOutput <- simulateResiduals(mod8, plot = F)

plot(simulationOutput)
```

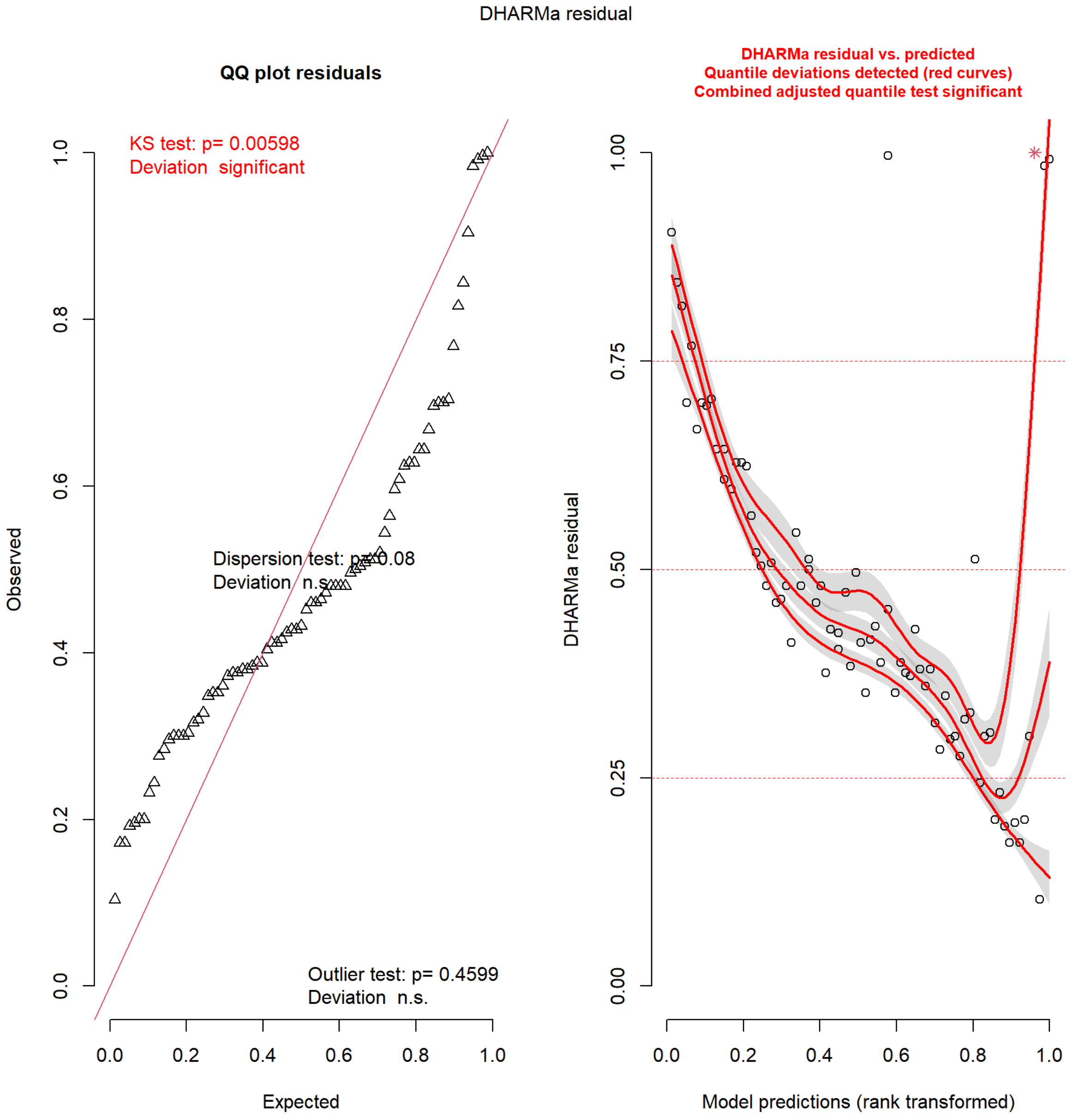

The model is not ok.

Log transformation

```
dat3<-dat %>% mutate(f_to_b_ratio = log(f_to_b_ratio))
```

```
mod9<-lmer(f_to_b_ratio ~ chemo+age+enterocolitis+

          stool_culture+ stool2+

          sepsis+ tazocin+ maxipime+ zithromax+meropenem+

          amikacin+vancomycin+colistin+tigecycline+

            (1|patient), data = dat3)

summary(mod9)
```

```
## Linear mixed model fit by REML. t-tests use Satterthwaite's method [
## lmerModLmerTest]
## Formula: f_to_b_ratio ~ chemo + age + enterocolitis + stool_culture +
##     stool2 + sepsis + tazocin + maxipime + zithromax + meropenem +
##     amikacin + vancomycin + colistin + tigecycline + (1 | patient)
##    Data: dat3
##
## REML criterion at convergence: 290.5
##
## Scaled residuals:
##      Min       1Q   Median       3Q      Max
## -1.95869 -0.61063 -0.03087  0.46720  2.26853
##
## Random effects:
##  Groups   Name        Variance Std.Dev.
##  patient  (Intercept) 0.5332   0.7302
##  Residual             4.0352   2.0088
## Number of obs: 77, groups:  patient, 29
##
## Fixed effects:
##                                   Estimate Std. Error       df t value
## (Intercept)                       -0.52900    1.91797 56.96795  -0.276
## chemoYes                          -0.37891    0.62597 50.02456  -0.605
## age                                0.02003    0.06757 19.84671   0.296
## enterocolitisYes                   1.41805    0.78108 25.67280   1.815
## stool_cultureE. coli               3.92043    3.17536 53.42532   1.235
## stool_cultureEnterococcus          3.11757    3.29452 55.66930   0.946
## stool_cultureKlebsiella pneumoniae  0.19536    2.31151 55.70512   0.085
## stool_cultureNegative              3.54237    3.08502 56.08424   1.148
## stool2E. coli                     -1.50856    3.56606 53.93892  -0.423
## stool2Klebsiella pneumoniae        2.48488    2.68350 54.93179   0.926
## stool2Negative                     0.48435    3.63108 55.22934   0.133
## sepsisYes                          0.12480    0.62749 27.01706   0.199
## tazocinyes                         0.67805    0.75388 36.62908   0.899
## maxipimeyes                       -0.47794    0.62964 56.79342  -0.759
## zithromaxyes                      -1.07280    1.68719 56.78045  -0.636
## meropenemyes                      -0.92229    0.78408 52.00738  -1.176
## amikacinyes                        0.76694    0.64769 54.69495   1.184
## vancomycinyes                     -0.58129    0.58058 40.62824  -1.001
## colistinyes                       -1.88362    1.79378 42.63801  -1.050
## tigecyclineyes                     0.69275    1.56550 56.58128   0.443
##                                   Pr(>|t|)
## (Intercept)                         0.7837
## chemoYes                            0.5477
## age                                 0.7700
## enterocolitisYes                    0.0811 .
## stool_cultureE. coli                0.2224
## stool_cultureEnterococcus           0.3481
## stool_cultureKlebsiella pneumoniae   0.9329
## stool_cultureNegative               0.2557
## stool2E. coli                       0.6740
## stool2Klebsiella pneumoniae         0.3585
## stool2Negative                      0.8944
## sepsisYes                           0.8438
## tazocinyes                          0.3743
```

```
## maxipimeyes                          0.4510
## zithromaxyes                         0.5274
## meropenemyes                         0.2448
## amikacinyes                          0.2415
## vancomycinyes                        0.3226
## colistinyes                          0.2996
## tigecyclineyes                       0.6598
## ---
## Signif. codes:  0 '***' 0.001 '**' 0.01 '*' 0.05 '.' 0.1 ' ' 1
```

Model diagnostics

```
simulationOutput <- simulateResiduals(mod9, plot = F)

plot(simulationOutput)
```

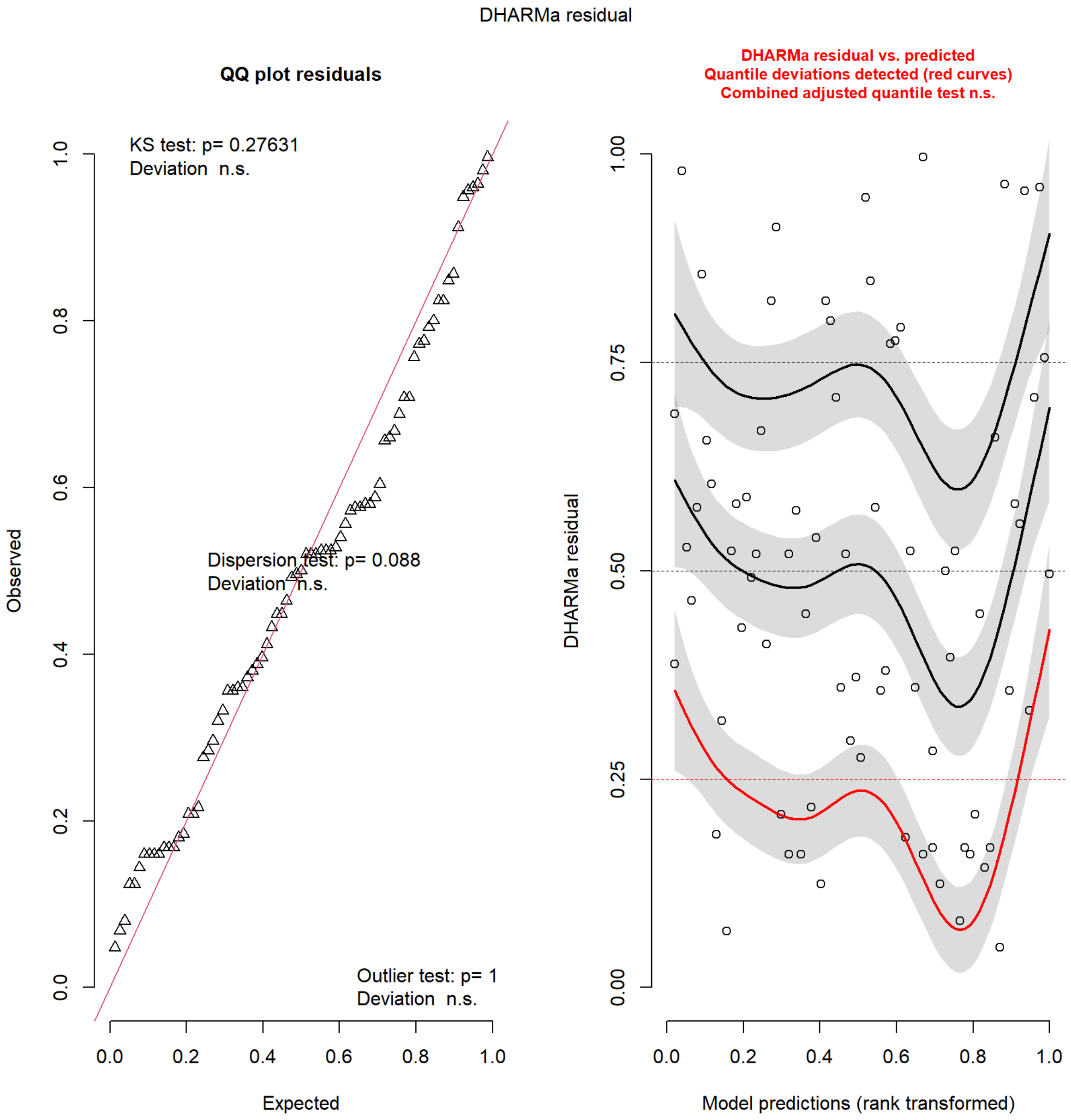

The model is nearly ok.

Trim model

```
step_res<-step(mod9, reduce.random = F)

mod10<-get_model(step_res)

summary(mod10)
```

```
## Linear mixed model fit by REML. t-tests use Satterthwaite's method [
## lmerModLmerTest]
## Formula: f_to_b_ratio ~ stool2 + (1 | patient)
##    Data: dat3
##
## REML criterion at convergence: 323.6
##
## Scaled residuals:
##     Min      1Q  Median      3Q     Max
## -1.7992 -0.5792 -0.1599  0.5427  2.7321
##
## Random effects:
##  Groups   Name        Variance Std.Dev.
##  patient  (Intercept) 0.000    0.000
##  Residual             4.275    2.068
## Number of obs: 77, groups:  patient, 29
##
## Fixed effects:
##                              Estimate Std. Error     df t value Pr(>|t|)
## (Intercept)                    -1.310      1.194 73.000  -1.097   0.2762
## stool2E. coli                   2.824      1.238 73.000   2.282   0.0254 *
## stool2Klebsiella pneumoniae     2.772      1.335 73.000   2.077   0.0413 *
## stool2Negative                  4.362      1.273 73.000   3.428   0.0010 **
## ---
## Signif. codes:  0 '***' 0.001 '**' 0.01 '*' 0.05 '.' 0.1 ' ' 1
##
## Correlation of Fixed Effects:
##             (Intr) st2E.c stl2Kp
## stool2E.col -0.964
## stl2Klbsllp -0.894  0.863
## stool2Negtv -0.938  0.905  0.839
## optimizer (nloptwrap) convergence code: 0 (OK)
## boundary (singular) fit: see help('isSingular')
```

Only stool2 samples are important.

```
simulationOutput <- simulateResiduals(mod10, plot = F)

plot(simulationOutput)
```

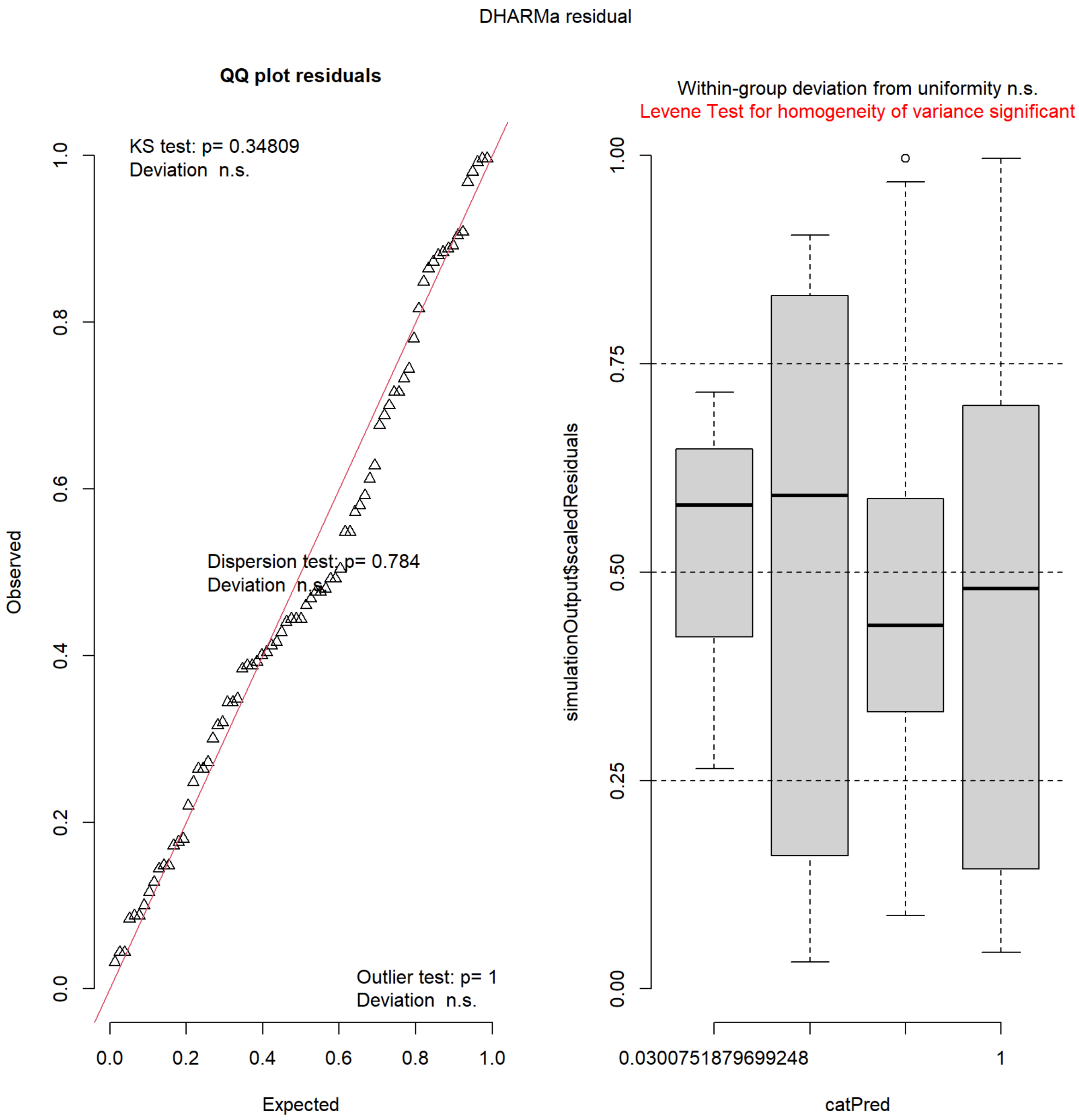

The model is not ok.

```
step_res
```

```
## Backward reduced random-effect table:
##
##                Eliminated npar  logLik    AIC     LRT Df Pr(>Chisq)
## <none>                      22 -145.23 334.46
## (1 | patient)          0   21 -145.42 332.84 0.38696  1     0.5339
##
## Backward reduced fixed-effect table:
## Degrees of freedom method: Satterthwaite
##
##               Eliminated Sum Sq Mean Sq NumDF  DenDF F value   Pr(>F)
## sepsis                 1  0.160  0.1596     1 27.017  0.0396 0.843835
## age                    2  0.408  0.4077     1 21.073  0.1017 0.752891
## tigecycline            3  0.858  0.8583     1 58.948  0.2169 0.643128
## stool_culture          4 10.858  2.7145     4 59.546  0.6850 0.605166
## zithromax              5  1.510  1.5096     1 63.634  0.3759 0.542016
## chemo                  6  1.499  1.4986     1 53.314  0.3784 0.541085
## maxipime               7  2.701  2.7010     1 65.983  0.6813 0.412106
## vancomycin             8  2.200  2.2004     1 43.285  0.5486 0.462877
## amikacin               9  3.866  3.8662     1 67.840  0.9622 0.330118
## colistin              10  4.131  4.1315     1 69.000  1.0184 0.316429
## meropenem             11  8.782  8.7816     1 70.000  2.1640 0.145756
## tazocin               12  9.297  9.2966     1 71.000  2.2540 0.137704
## enterocolitis         13  9.936  9.9362     1 72.000  2.3678 0.128243
## stool2                 0 68.065 22.6883     3 73.000  5.3072 0.002299 **
## ---
## Signif. codes:  0 '***' 0.001 '**' 0.01 '*' 0.05 '.' 0.1 ' ' 1
##
## Model found:
## f_to_b_ratio ~ stool2 + (1 | patient)
```

Add enterocolitis

```
mod11<-lmer(f_to_b_ratio ~ stool2 + enterocolitis+

              (1|patient), data = dat3)

summary(mod11)
```

```
## Linear mixed model fit by REML. t-tests use Satterthwaite's method [
## lmerModLmerTest]
## Formula: f_to_b_ratio ~ stool2 + enterocolitis + (1 | patient)
##    Data: dat3
##
## REML criterion at convergence: 320.4
##
## Scaled residuals:
##     Min      1Q  Median      3Q     Max
## -2.1043 -0.6548 -0.1359  0.6275  2.8349
##
## Random effects:
##  Groups   Name        Variance Std.Dev.
##  patient  (Intercept) 0.000    0.000
##  Residual             4.196    2.048
## Number of obs: 77, groups:  patient, 29
##
## Fixed effects:
##                             Estimate Std. Error      df t value Pr(>|t|)
## (Intercept)                  -1.9130     1.2460 72.0000  -1.535 0.129092
## stool2E. coli                 3.2688     1.2599 72.0000   2.595 0.011470 *
## stool2Klebsiella pneumoniae   3.3002     1.3661 72.0000   2.416 0.018240 *
## stool2Negative                4.6777     1.2773 72.0000   3.662 0.000474 ***
## enterocolitisYes              0.9050     0.5881 72.0000   1.539 0.128243
## ---
## Signif. codes:  0 '***' 0.001 '**' 0.01 '*' 0.05 '.' 0.1 ' ' 1
##
## Correlation of Fixed Effects:
##             (Intr) st2E.c stl2Kp stl2Ng
## stool2E.col -0.963
## stl2Klbsllp -0.901  0.870
## stool2Negtv -0.929  0.906  0.842
## entercltsYs -0.315  0.230  0.251  0.160
## optimizer (nloptwrap) convergence code: 0 (OK)
## boundary (singular) fit: see help('isSingular')
```

Model diagnostics

```
simulationOutput <- simulateResiduals(mod11, plot = F)

plot(simulationOutput)
```

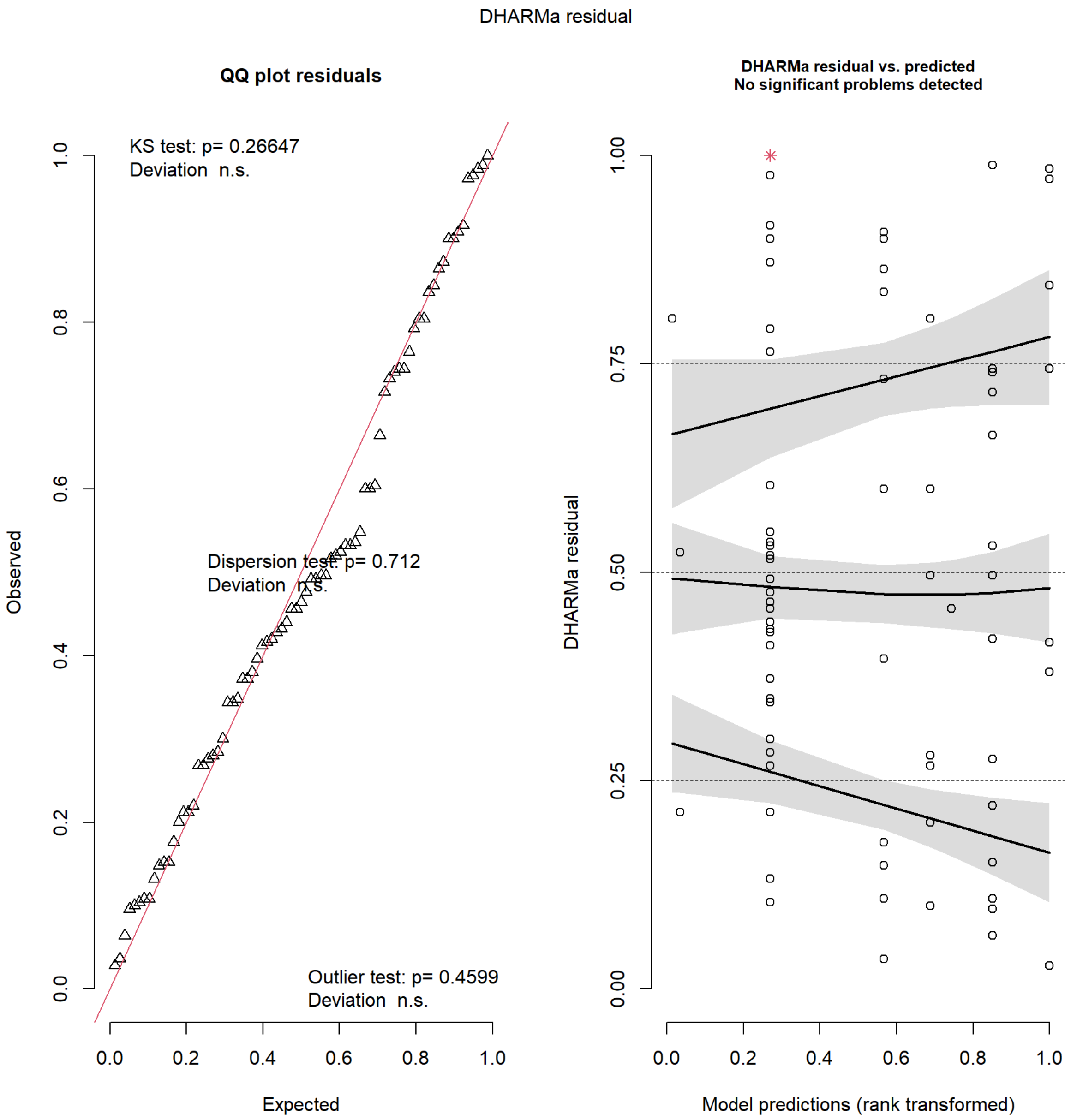

The model is ok.

- The presence of E.coli in stool2 is associated with exp(3.2688)-1 = 2528% increase in f_to_b_ratio than patients having A.baumannii in stool2.
- The presence of K.pneumonia in stool2 is associated with exp(3.3002)-1 = 2611% increase in f_to_b_ratio than patients having A.baumannii in stool2.
- The negative stool2 sample is associated with exp(4.6777)-1 = 10652% increase in f_to_b_ratio than patients having A.baumannii on stool2.

```
df<-ggpredict(mod11, terms = ~ stool2+ enterocolitis)

df %>% dplyr::rename(stool2 = x, enterocolitis = group) %>%

  mutate(stool_entero = paste(stool2, enterocolitis, sep = "_")) %>%


  ggplot(aes(x = predicted, y = stool_entero))+

  geom_point()+ geom_line(aes(group = 1))
```

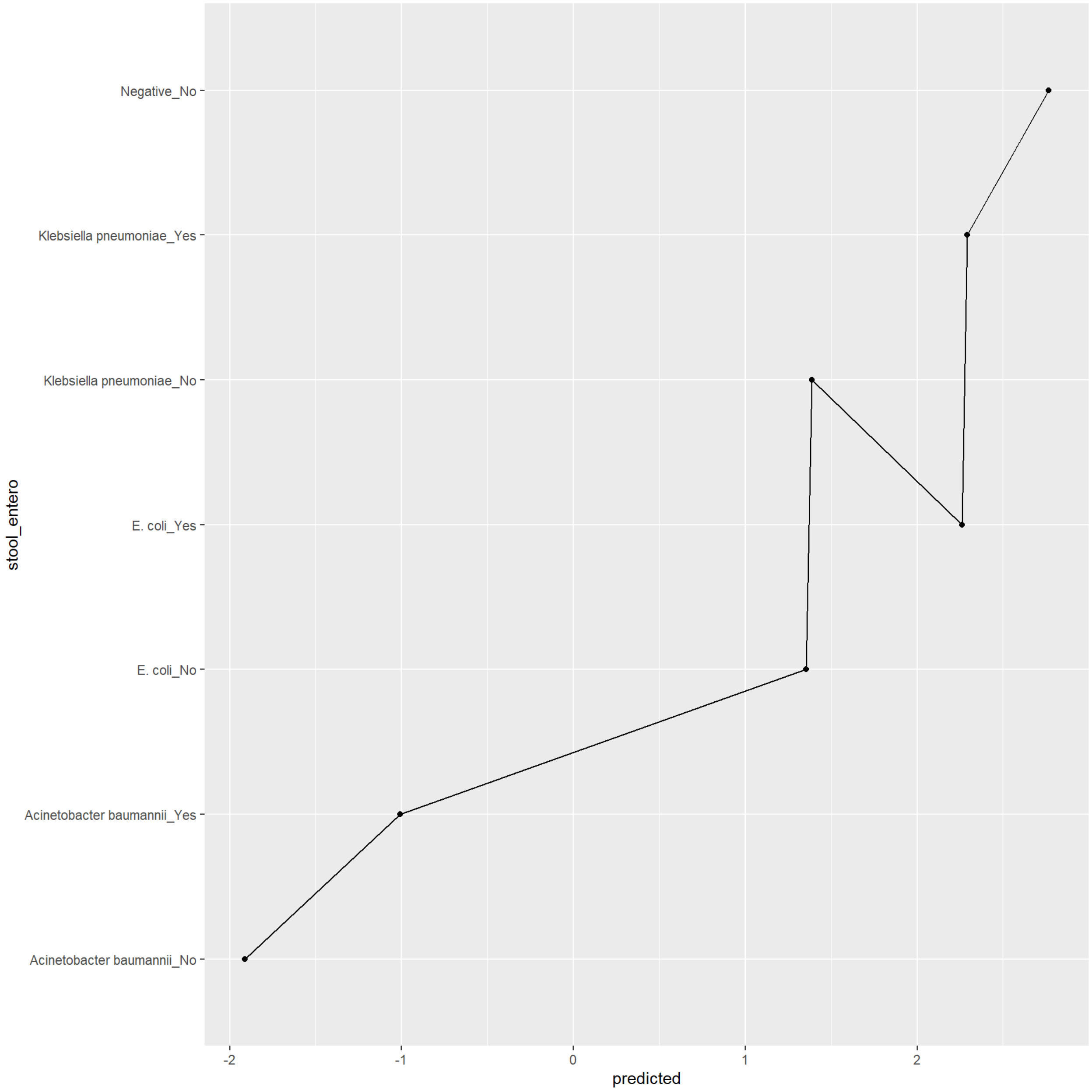

- There are some negative f_to_b_ratio. Is that ok?
- The highest predicted f_to_b_ratio when stool2 is neagtive and no enterocolitis.
- The lowest predicted f_to_b_ratio when stool2 has A.baumanni and no enterocolitis.

p_to_f_ratio

Multiple linear model

```
mod12<-lm(p_to_f_ratio ~ chemo+age+enterocolitis+

          stool_culture+ stool2+

          sepsis+ tazocin+ maxipime+ zithromax+meropenem+

          amikacin+vancomycin+colistin+tigecycline, data = dat)
```

Model diagnostics

```
simulationOutput <- simulateResiduals(mod12, plot = F)

plot(simulationOutput)
```

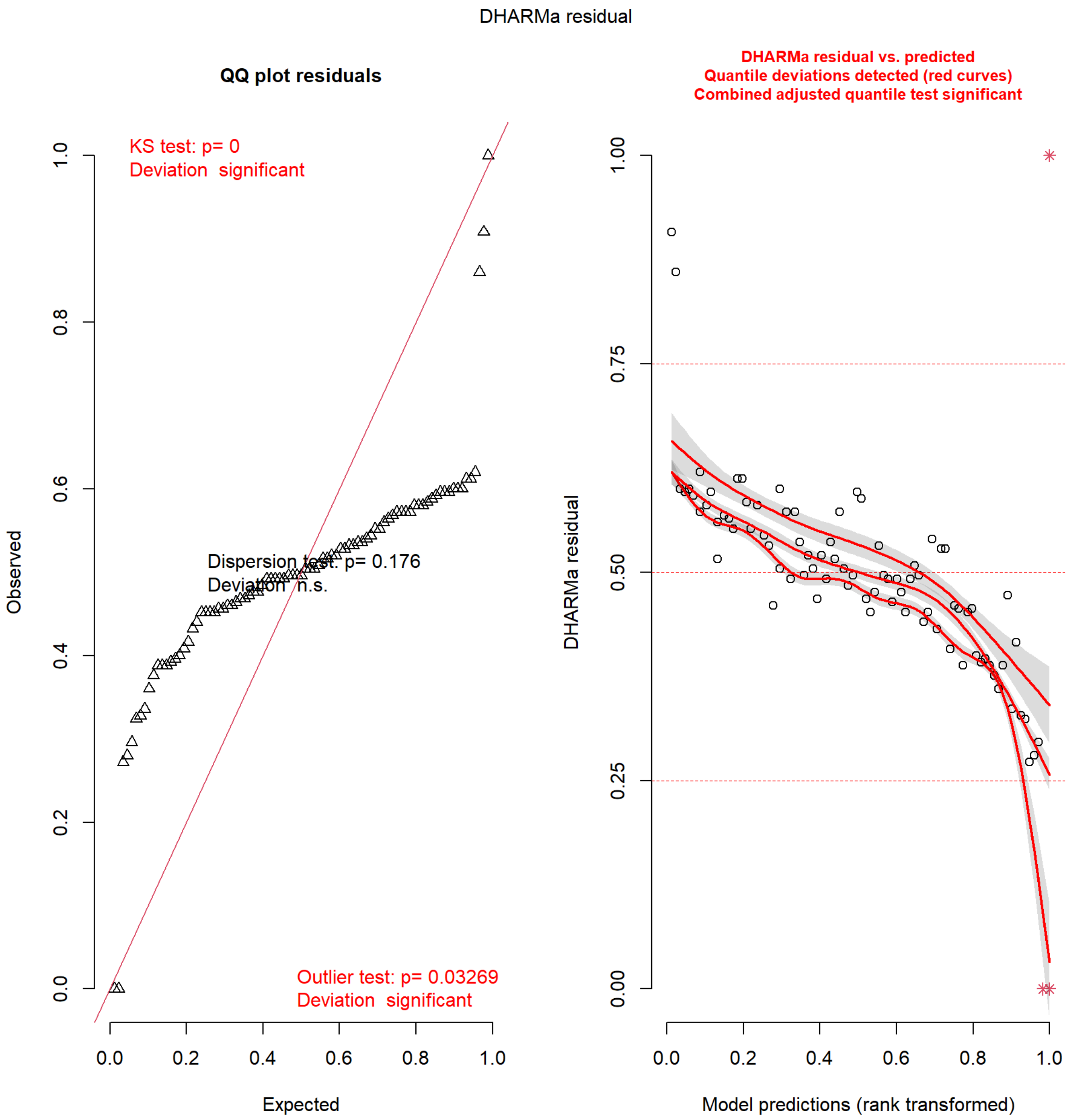

The model is not ok.

Linear mixed model

```
mod13<-lmer(p_to_f_ratio ~ chemo+age+enterocolitis+

          stool_culture+ stool2+

          sepsis+ tazocin+ maxipime+ zithromax+meropenem+

          amikacin+vancomycin+colistin+tigecycline+

            (1|patient), data = dat)
```

Model diagnostics

```
simulationOutput <- simulateResiduals(mod13, plot = F)

plot(simulationOutput)
```

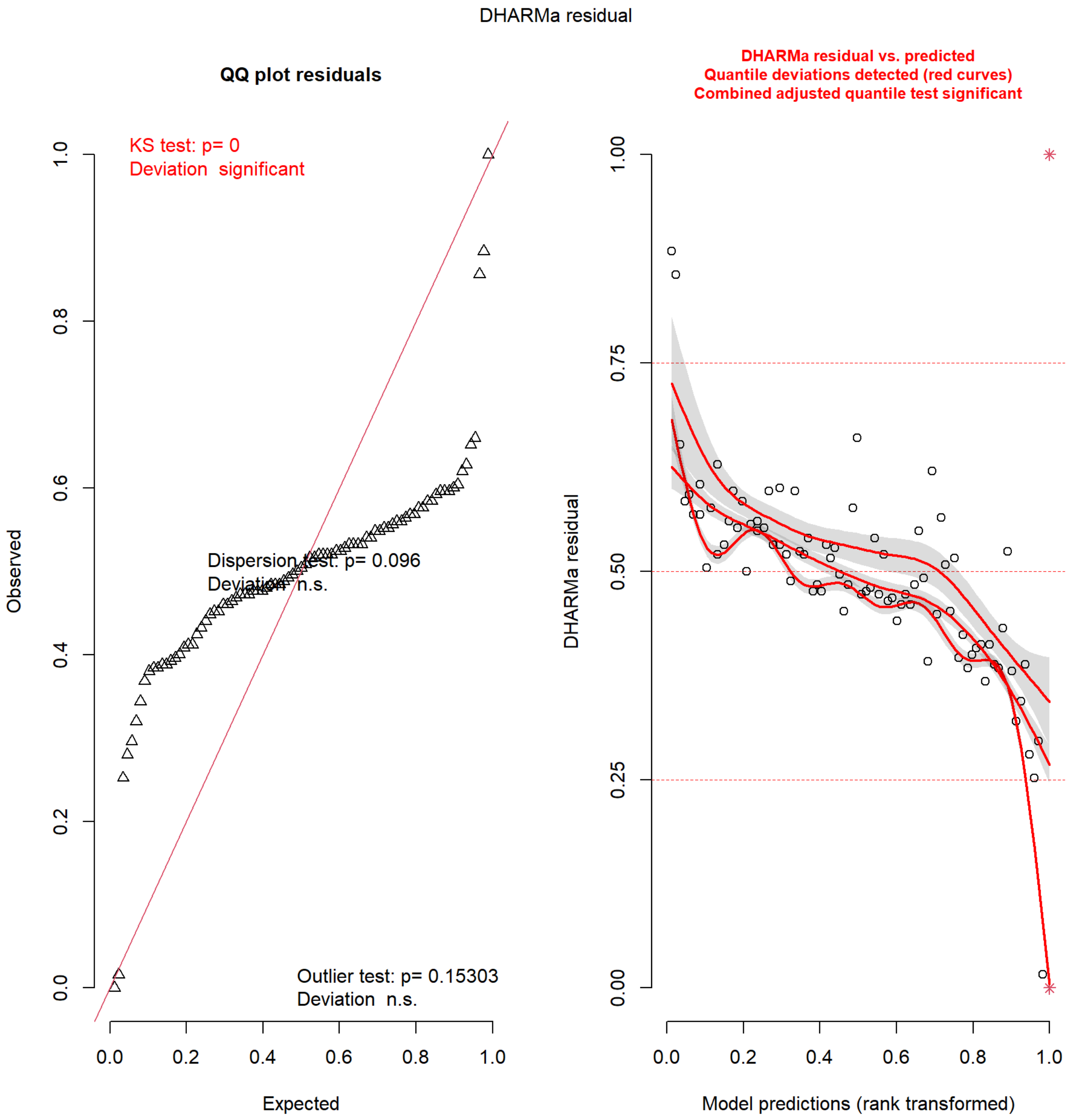

The model is not ok.

Log transformation

```
dat4<-dat %>% mutate(p_to_f_ratio = log1p(p_to_f_ratio))
```

```
mod14<-lmer(p_to_f_ratio ~ chemo+age+enterocolitis+

          stool_culture+ stool2+

          sepsis+ tazocin+ maxipime+ zithromax+meropenem+

          amikacin+vancomycin+colistin+tigecycline+

            (1|patient), data = dat4)
```

Model diagnostics

```
simulationOutput <- simulateResiduals(mod14, plot = F)

plot(simulationOutput)
```

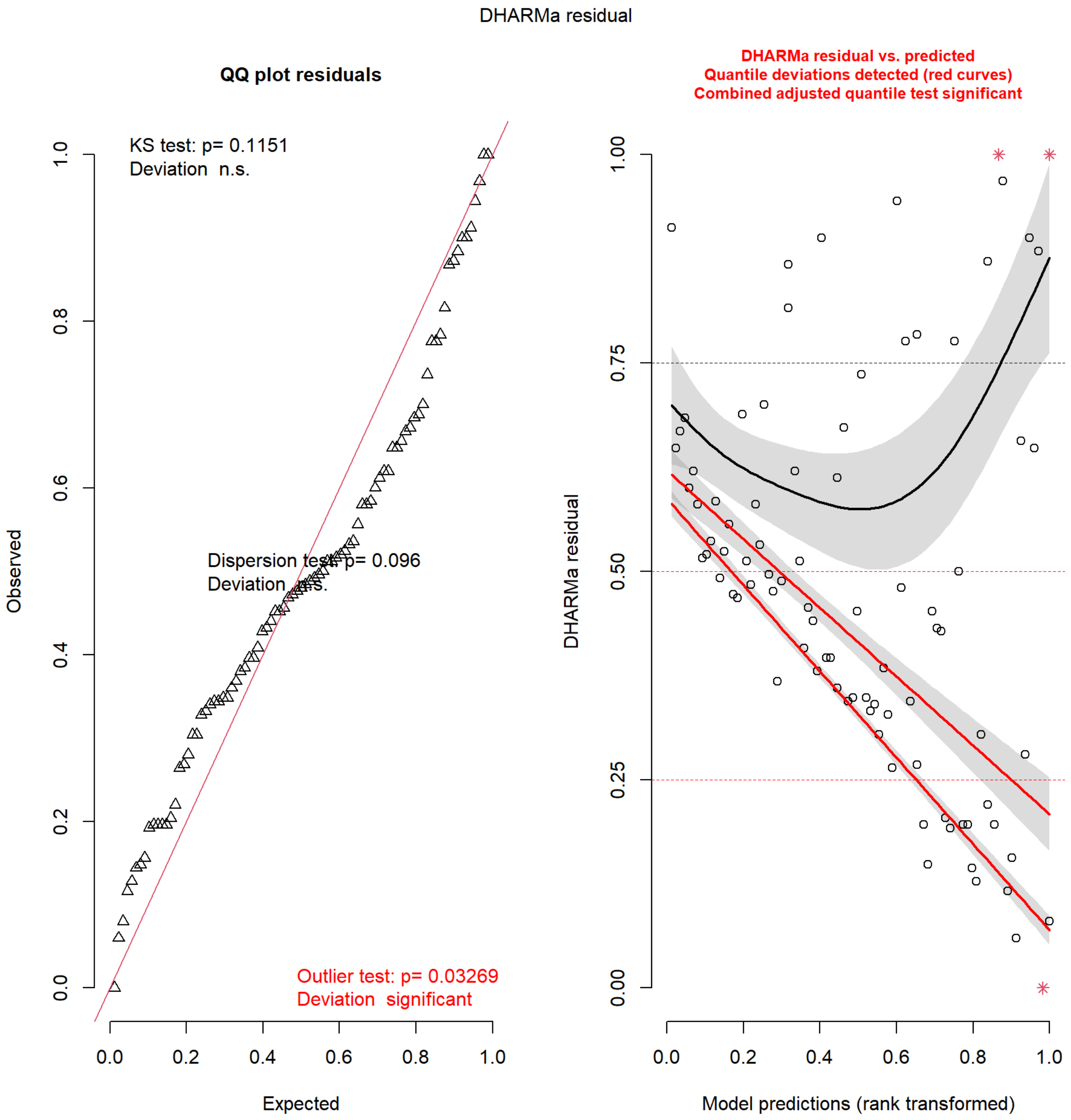

The model is not ok.

Trim model

```
step_res<-step(mod14, reduce.random = F)

mod15<-get_model(step_res)

summary(mod15)
```

```
## Linear mixed model fit by REML. t-tests use Satterthwaite's method [
## lmerModLmerTest]
## Formula: p_to_f_ratio ~ age + stool2 + colistin + tigecycline + (1 | patient)
##    Data: dat4
##
## REML criterion at convergence: 133.7
##
## Scaled residuals:
##     Min      1Q  Median      3Q     Max
## -2.8252 -0.4910 -0.2417  0.4114  4.0943
##
## Random effects:
##  Groups   Name        Variance Std.Dev.
##  patient  (Intercept) 0.0000   0.0000
##  Residual             0.2406   0.4906
## Number of obs: 87, groups:  patient, 29
##
## Fixed effects:
##                              Estimate Std. Error       df t value Pr(>|t|)
## (Intercept)                   3.05307    0.36100 80.00000   8.457 1.02e-12 ***
## age                          -0.03023    0.01157 80.00000  -2.613  0.01070 *
## stool2E. coli                -2.53116    0.35854 80.00000  -7.060 5.45e-10 ***
## stool2Klebsiella pneumoniae -2.10116    0.35627 80.00000  -5.898 8.42e-08 ***
## stool2Negative               -2.81281    0.36348 80.00000  -7.739 2.63e-11 ***
## colistinyes                   0.95080    0.35101 80.00000   2.709  0.00826 **
## tigecyclineyes               -0.65929    0.31767 80.00000  -2.075  0.04116 *
## ---
## Signif. codes:  0 '***' 0.001 '**' 0.01 '*' 0.05 '.' 0.1 ' ' 1
##
## Correlation of Fixed Effects:
##             (Intr) age    st2E.c stl2Kp stl2Ng clstny
## age         -0.177
## stool2E.col -0.944 -0.084
## stl2Klbsllp -0.888 -0.093  0.906
## stool2Negtv -0.948 -0.006  0.940  0.889
## colistinyes  0.017 -0.100 -0.006  0.010  0.026
## tigecyclnys -0.385 -0.090  0.403  0.327  0.365 -0.719
## optimizer (nloptwrap) convergence code: 0 (OK)
## boundary (singular) fit: see help('isSingular')
```

Only age, stool2 samples, colistin, and tigecycline are important.

```
simulationOutput <- simulateResiduals(mod15, plot = F)

plot(simulationOutput)
```

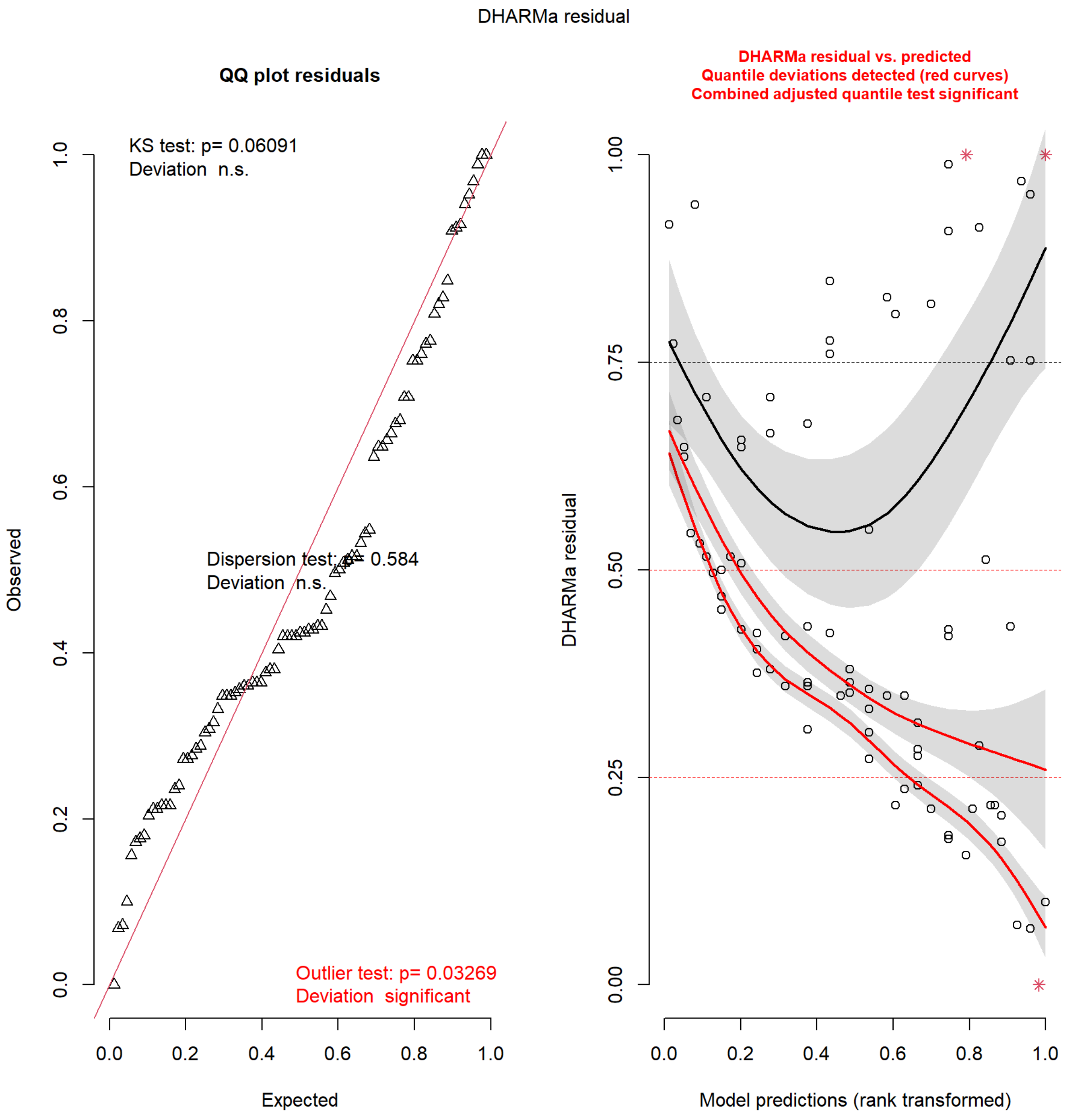

The model is not ok. It is better not use this outcome.
